# Malignant Transformation of Normal Oral Tissue to Dysplasia and Early Oral Squamous Cell Carcinoma: An *In Silico* Transcriptomics Approach

**DOI:** 10.1155/2024/6260651

**Published:** 2024-09-18

**Authors:** Shokoofeh Jamshidi, Matina Tavangar, Setareh Shojaei, Amir Taherkhani

**Affiliations:** ^1^Department of Oral and Maxillofacial Pathology, School of Dentistry, Dental Research Center, Hamadan University of Medical Sciences, Hamadan, Iran; ^2^Department of Oral and Maxillofacial Pathology, School of Dentistry, Hamadan University of Medical Sciences, Hamadan, Iran; ^3^Research Center for Molecular Medicine, Hamadan University of Medical Sciences, Hamadan, Iran

**Keywords:** biomarkers, dysplasia, oral squamous cell carcinoma, pathogenesis, prognosis

## Abstract

**Background:** Oral squamous cell carcinoma (OSCC) is a prevalent and aggressive form of head and neck cancer, often diagnosed at advanced stages. Elucidating the molecular mechanisms involved in the malignant transformation from normal oral tissue to oral preinvasive lesions (OPL) and primary OSCC could facilitate early diagnosis and improve therapeutic strategies.

**Methods:** Differentially expressed genes (DEGs) were identified from the GSE30784 dataset by comparing normal oral tissue, oral dysplasia, and primary OSCC samples. Cross-validation was performed using an independent RNA-seq dataset, GSE186775. Protein–protein interaction (PPI) network analysis, gene ontology annotation, and pathway enrichment analysis were conducted on the common DEGs. Hub genes were identified, and their prognostic significance was evaluated using survival analysis. Transcription factor (TF) enrichment analysis, cross-validation, and immunohistochemistry analyses were also performed.

**Results:** A total of 226 proteins and 677 interactions were identified in the PPI network, with 34 hub genes, including FN1, SERPINE1, PLAUR, THBS1, and ITGA6. Pathways such as “Formation of the cornified envelope,” “Keratinization,” and “Developmental biology” were enriched. Overexpression of SERPINE1, PLAUR, THBS1, and ITGA6 correlated with poor prognosis, while upregulation of CALML5 and SPINK5 was associated with favorable outcomes. NFIB emerged as the most significant TF-regulating hub genes. Immunohistochemistry validated ITGA6 overexpression in primary OSCC. Cross-validation using the RNA-seq dataset supported the involvement of critical genes in the malignant transformation process.

**Conclusion:** This study identified vital genes, pathways, and prognostic markers involved in the malignant transformation from normal oral tissue to OPL and primary OSCC, providing insights for early diagnosis and targeted therapy development. Cross-validation with an independent RNA-seq dataset and immunohistochemistry reinforced the findings, supporting the robustness of the identified molecular signatures.

## 1. Introduction

Head and neck squamous cell carcinoma (HNSCC) is characterized by malignancy originating from the upper aerodigestive epithelium [1]. Among HNSCC cases, oral squamous cell carcinoma (OSCC) stands as the sixth most prevalent cancer globally. In 2018, OSCC affected over 350,000 individuals worldwide, resulting in an estimated 170,000 deaths [2, 3]. Individuals who consume alcohol or use tobacco products face a heightened risk of OSCC [2]. The primary approaches for addressing OSCC include surgical removal of the tumor, the combination of cetuximab and PD-1 inhibitors, radiotherapy, and chemoradiation therapy. Despite these approaches, the 5-year survival rate for OSCC patients remains low due to drug resistance and high recurrence [4–10]. Moreover, OSCC patients are often diagnosed at advanced stages, contributing to unfavorable outcomes [11]. Hence, the elucidation of underlying pathways, biological processes (PBs), and essential genes involved in the malignant transformation from normal oral tissue to oral preinvasive lesions (OPL) and primary OSCC could facilitate early diagnosis and enhance the effectiveness of therapeutic strategies [12].

**Figure 1:**
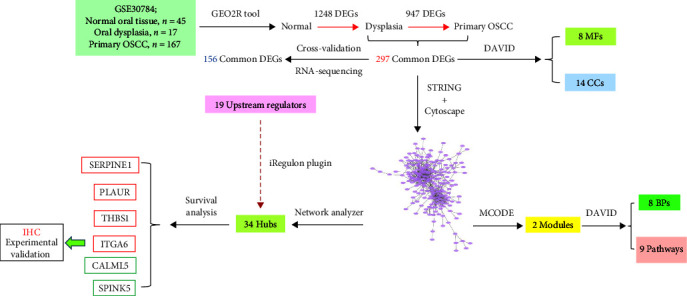
The visual summary of the current study.

It has been documented that 20% of cases presenting with oral dysplasia progress to OSCC [13]. Consequently, it was proposed that common genes may play a role in the malignant transformation of normal oral tissue to dysplasia and primary OSCC. Furthermore, it was posited that these genes could be correlated with the survival rates of OSCC patients. These prognostic markers may identify high-risk patients with dysplasia or early OSCC, paving the way for more efficacious treatment. To this end, the following sequential steps were undertaken: (1) identification of differentially expressed genes (DEGs) in dysplasia in comparison to normal oral tissues, (2) determination of DEGs in primary OSCC as opposed to dysplasia, (3) identification of common DEGs in the two stages of malignant transformation through Venn diagram analysis, (4) construction of a protein–protein interaction (PPI) network based on the common DEGs, (5) identification of hub genes within the PPI network, and (6) execution of Kaplan–Meier analysis to explore the prognostic impact of the hub genes.  Figure 1 provides a schematic overview of the entire study.

To assess our hypothesis, we chose to reanalyze the dataset GSE30784, as curated by Chen et al. [14]. This dataset was explicitly designed to unveil gene expression profiles in early OSCC, oral dysplasia, and normal oral tissue devoid of oral cancer or preneoplastic oral lesions (controls). The tissue samples were procured from patients and individuals at the University of Washington Medical Center, Harborview Medical Center, and the VA Puget Sound Health Care System in Seattle, Washington, between December 1, 2003, and April 17, 2007. Healthy control samples were derived from individuals who underwent tonsillectomy or oral surgery for noncancer disorders. All volunteers included in the study were over 18 years old. Ethical approval for the present study was obtained from the Ethics Committee of Hamadan University of Medical Sciences, Hamadan, Iran (ethics no. IR.UMSHA.REC.1400.734).

## 2. Methods

### 2.1. Data Processing of DEGs

The gene expression dataset GSE30784 was sourced from the GPL570 platform ([HG-U133_Plus_2] Affymetrix Human Genome U133 Plus 2.0 Array), accessible at https://www.ncbi.nlm.nih.gov/geo/. Notably, the present study did not involve human or animal samples. The dataset comprised oral normal tissues (*n* = 45), oral dysplasia lesions (*n* = 17), and primary OSCC samples (*n* = 167). DEGs in oral dysplasia versus normal oral tissue and early OSCC versus oral dysplasia were identified using the GEO2R online tool [15, 16]. Probes associated with unannotated genes were excluded, and the average expression value was considered for genes represented by multiple probes.

The microarray data were normalized using the Benjamini and Hochberg method [17], a well-established statistical technique for controlling false discovery rates (FDR) in microarray studies. This approach is reliable for managing multiple comparisons without excessively sacrificing statistical power. While there are inherent limitations related to assumptions about test independence and correlation structures among SNPs, these limitations do not significantly detract from its overall reliability compared to more conservative alternatives like the Bonferroni correction [18–20].

Genes meeting the FDR < 0.05 and |log_2_FC| > 1 criteria were designated DEGs. The common DEGs orchestrating the malignant transformation from normal oral tissue to oral dysplasia and primary OSCC were discerned using the Venn diagram tool, accessible at https://bioinformatics.psb.ugent.be/webtools/Venn/.

### 2.2. PPI Network Construction

The Search Tool for the Retrieval of Interacting Genes (STRING) database [21], accessible at http://string-db.org/, is a valuable resource for exploring protein connections. This study utilized the STRING database to elucidate physical or functional interactions among the common DEGs. A combined score of 0.4 was set as the threshold to consider edges between two proteins as significant [22]. To enhance clarity, single nodes within the PPI network were excluded from the graph [23]. Subsequently, the protein interaction map (PIM) was imported into Cytoscape 3.9.1 software [24], accessible at www.cytoscape.org/, for conducting topological analyses on the network. Nodes meeting the degree and betweenness centrality criteria above the average value of the nodes within the PIM were identified as hub genes using the network analyzer tool [23]. Additionally, the graph's most significant modules (clusters) were discerned using the MCODE plugin [25], as previously described in our study [26].

### 2.3. Gene Ontology (GO) Annotation and Pathway Analysis

The Database for Annotation, Visualization, and Integrated Discovery (DAVID 6.8) tools [27], accessible at https://david.ncifcrf.gov/, were employed to discern signaling pathways and GO annotations, encompassing BPs, cellular components (CCs), and molecular functions (MFs) significantly enriched in the malignant transformation from normal oral mucosa to oral dysplasia and primary OSCC. The cutoff condition was set at an adjusted *p*-value < 0.05. Within the DAVID database, the Kyoto Encyclopedia of Genes and Genomes [28] and Reactome [29] databases were selected to identify deregulated pathways. The common DEGs were employed for MF and CC annotation analysis, while the clustered genes were allocated for BP and pathway enrichment analysis [30].

### 2.4. Transcription Factor (TF) Enrichment Analysis

Building on our prior report [31], the iRegulon plugin [32] within the Cytoscape tool was employed to identify upstream TFs governing the regulation of hub genes. A mounting body of evidence indicates that aberrant expression of TFs is implicated in various human disorders, including cancers. Consequently, elucidating the upstream regulators involved in cancer onset and progression holds the potential to unveil new drug targets for cancer therapy [33].

### 2.5. Survival Analysis of Hub Genes

The Gene Expression Profiling Interactive Analysis 2 (GEPIA2) database [34, 35] was utilized to assess the prognostic significance of the hub genes in patients with HNSCC. The GEPIA2 database facilitates the generation of Kaplan–Meier curves for survival analysis. A *p*-value < 0.05 was considered indicative of statistical significance for both the log-rank test and hazard ratio (HR), determining the prognostic impact of the hub genes.

### 2.6. Gene Expression Patterns of Prognostic Markers in the TCGA and GTEx Databases

The expression patterns of prognostic markers in this study were examined at the mRNA levels in HNSCC tissues (*n* = 519) and normal specimens (*n* = 44). This analysis was conducted using boxplot visualization through the GEPIA2 web server [34, 35].

### 2.7. Cross-Validation Analysis Using an Independent Dataset

To further solidify our findings, an independent dataset for DEGs was reanalyzed. This provided corroborative evidence to support a more robust conclusion. For this analysis, we opted for the RNA-sequencing (RNA-seq) dataset GSE186775 [36] deposited within the GEO database. This dataset is built upon the GPL21290 platform (Illumina HiSeq 3000) and encompasses 16 OSCC samples alongside 15 normal oral tissue samples.

The RNA-seq dataset was also normalized using the Benjamini and Hochberg [17] approach. DEGs were identified by comparing cancerous samples to their healthy counterparts, with a stringent FDR threshold of less than 0.05 and |log_2_FC| exceeding 1.0. These criteria ensured that the identified DEGs possess statistical significance.

### 2.8. Immunohistochemistry Analysis

In this study, immunohistochemical (IHC) analysis was utilized to evaluate the protein expression of a specific biomarker. The selection of this biomarker was based on several stringent criteria:

1. Enhanced expression in oral dysplastic lesions compared to normal oral mucosa, as substantiated by data from the gene expression dataset GSE30784.

2. Upregulated levels in primary OSCC specimens in contrast to dysplastic tissues, also corroborated by the GSE30784 dataset.

3. Overexpression in OSCC tissues relative to adjacent nonneoplastic tissues, validated using the RNA-sequencing dataset GSE186775.

4. Elevated mRNA expression levels in HNSCC compared to noncancerous tissues, as evidenced by data from the GEPIA2 database.

5. Correlation with poor prognostic outcomes in HNSCC, ensuring the biomarker's clinical relevance in assessing disease severity.

A total of 32 tissue specimens were subjected to IHC staining: 16 primary OSCC samples and 16 adjacent nonneoplastic oral mucosa tissues. These formalin-fixed, paraffin-embedded blocks were procured from Hamadan University of Medical Sciences (Iran), with patient demographics previously detailed [37]. Two independent pathologists authenticated the blocks. An anti-ITGA6 antibody (ab20142, Abcam, USA) was applied at a 1:100 dilution following the IHC protocol delineated by Yang et al. [38]. Immunostained cells were examined under a light microscope (Olympus BX41, Japan) utilizing the AnalySIS LS Starter software. Two pathologists autonomously quantified immunopositive cells employing the scoring system described by Agarwal et al. [39]:

Negative: <10% immunopositive cells;

+1:10%–30% immunopositive cells;

+2:30%–50% immunopositive cells;

+3: > 50% immunopositive cells.

Immunostaining intensity was further classified using a modified quick score system [40, 41, and 42]: negative, weak, moderate, and strong. Statistical analyses were conducted in R programing using the Mann–Whitney *U* test to compare results across groups. A *p*-value < 0.05 was considered statistically significant.

## 3. Results

### 3.1. Identification of DEGs

**Figure 2:**
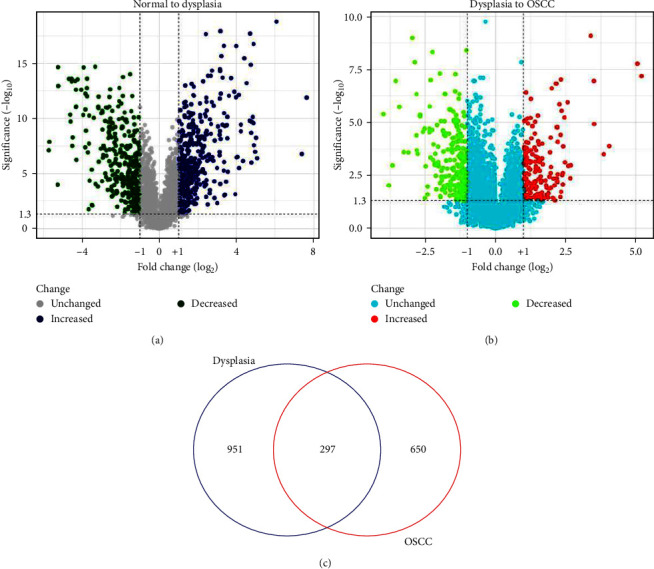
Volcano plot of the genes in (a) dysplasia compared to the normal oral tissues and (b) OSCC samples compared with the dysplasia specimens. (c) Common DEGs between dataset (a) and dataset (b). OSCC, oral squamous cell carcinoma; DEG, differentially expressed gene.

A total of 1248 DEGs, meeting the criteria of FDR < 0.05 and |log_2_FC| > 1, were identified, comprising 668 upregulated and 580 downregulated genes, in the comparison between oral dysplasia and normal oral specimens (Supporting Information S1: Table 1).  Figure 2a depicts the volcano plot of genes in the GSE30783 dataset associated with oral dysplasia (*n* = 17) and normal tissues (*n* = 45), generated using the Shiny apps web server, accessible at https://huygens.science.uva.nl/ [43].

Additionally, the GEO2R tool identified 947 genes, including 388 upregulated and 559 downregulated genes, as differentially expressed in early OSCC compared to oral dysplasia (Supporting Information S1: Table 2). The Shiny applications also facilitated the creation of a volcano plot for genes in the GSE30783 dataset associated with early OSCC (*n* = 167) and oral dysplasia (*n* = 17) (Figure 2b). The common DEGs from these analyses were identified using the Venn diagram, available at https://bioinformatics.psb.ugent.be/webtools/Venn/ (Figure 2c).

### 3.2. PPI Network Construction and Topological Analysis

**Figure 3:**
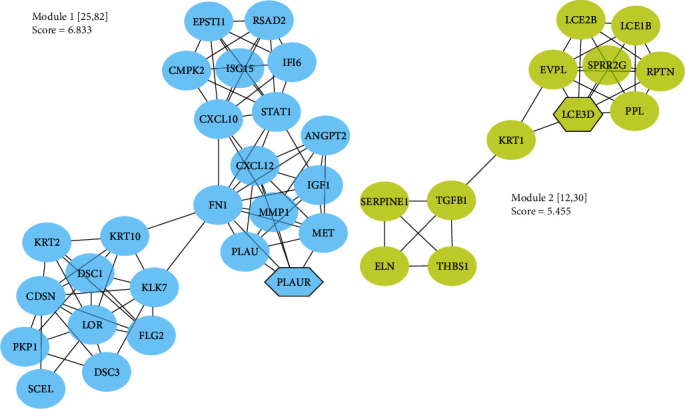
MCODE analysis revealed two clusters in the PPI network mediating the malignant transformation of normal oral mucosa to dysplasia and OSCC. Seed nodes are shown as hexagons. MCODE, molecular complex detection; PPI, protein–protein interaction.

The interconnected PIM based on the common DEGs identified 226 proteins and 677 edges. The MCODE plugin highlighted two condensed regions in the graph, namely module no. 1 and module no. 2 (Figure 3). The network analyzer tool computed the centralities of the nodes, revealing an average value for degree and betweenness centrality in the PIM as 5.99 and 0.02155, respectively. Consequently, 34 nodes exhibited degree and betweenness values surpassing the average of the nodes in the PPI network and were designated as hub proteins (Table 1). FN1 emerged as the most central node in the graph, with degree and betweenness values of 54 and 0.3, respectively.

### 3.3. GO Annotation and Pathway Analysis

**Figure 4:**
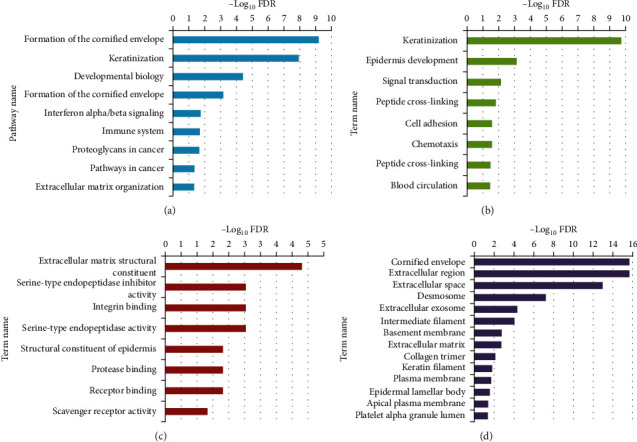
Gene set enrichment analysis revealed (a) pathways, (b) biological processes, (c) molecular functions, and (d) cellular components significantly involved in the malignant transformation of normal oral mucosa to dysplasia and OSCC.

According to the DAVID database analysis, nine pathways, eight BPs, eight MFs, and 14 CCs are implicated in initiating oral dysplasia and primary OSCC. Notably, “Formation of the cornified envelope” (R-HSA-6809371), “Keratinization” (R-HSA−6805567), and “Developmental biology” (R-HSA-1266738) emerged as the most significant pathways enriched in the malignant transformation from normal oral tissue to dysplasia and early OSCC (Figure 4a). Moreover, “Keratinization” (GO: 0031424), “Epidermis development” (GO: 0008544), and “Signal transduction” (GO: 0007165) stood out as the most substantial BPs deregulated in the malignant transformation of normal oral tissue (Figure 4b). In the MF category, “Extracellular matrix structural constituent” (GO: 0005201), “Serine-type endopeptidase inhibitor activity” (GO: 0004867), and “Integrin binding” (GO: 0005178) exhibited the highest −log_10_ FDR values (Figure 4c). Furthermore, “Cornified envelope” (GO: 0001533), “Extracellular region” (GO: 0005576), and “Extracellular space” (GO: 0005615) were identified as the most significant CCs enriched in the initiation of OPL and early OSCC (Figure 4d).

### 3.4. Upstream Regulators of Hub Genes

**Figure 5:**
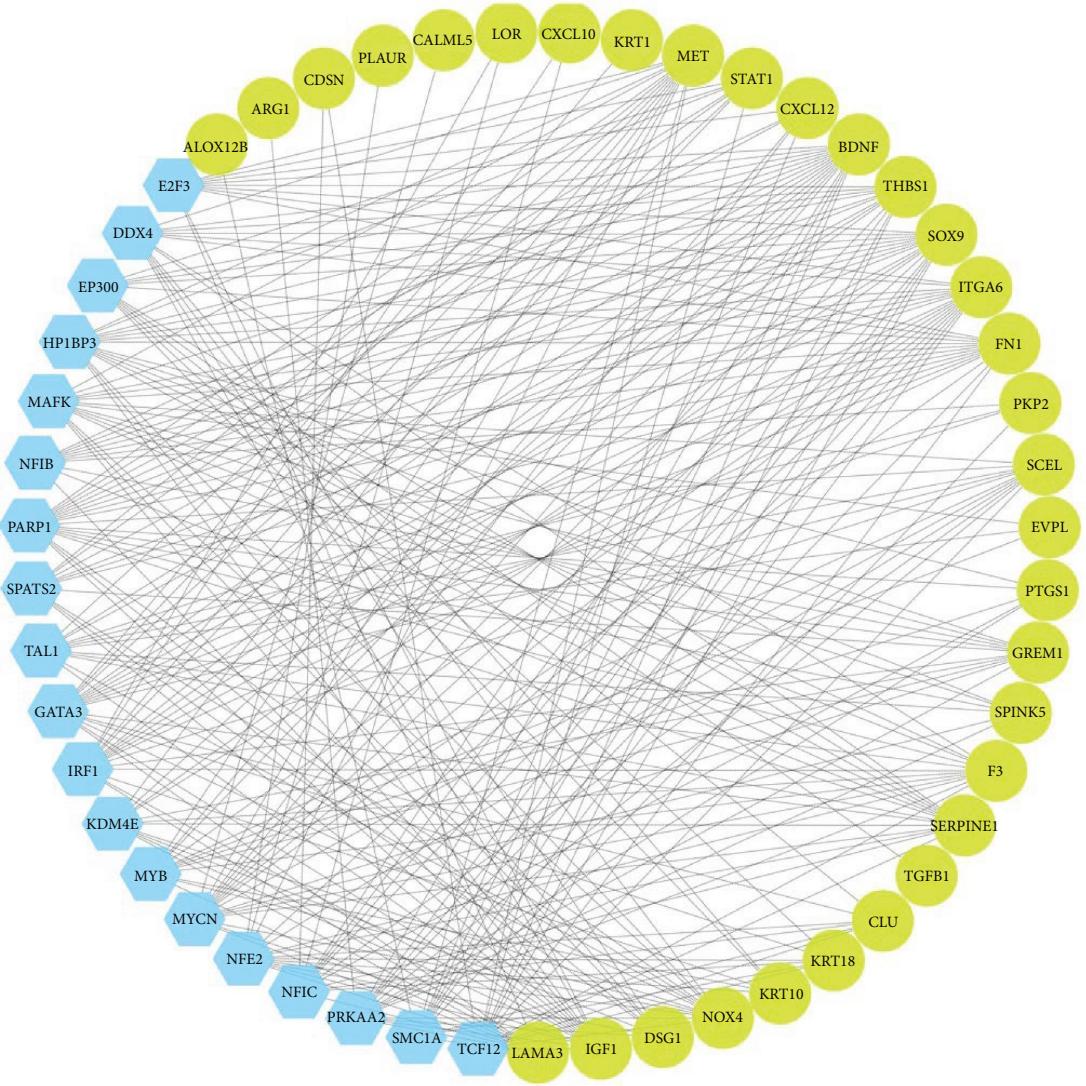
A directed gene regulatory network mediating the malignant transformation of healthy oral mucosa to dysplasia and OSCC. Circles and hexagons represent hub genes and their upstream transcription factors, respectively.

The iRegulon app identified 19 TFs as upstream regulators of hub genes involved in the malignant transformation from normal oral tissue to OPL and early OSCC. Notably, NFIB exhibited the most significant result, meeting the criteria of a normalized enrichment score (NES) of 5.365. It regulated 10 targets, including FN1, SOX9, THBS1, SERPINE1, ITGA6, GREM1, CXCL12, IGF1, BDNF, and PKP2 (Table 2). A gene regulatory network (GRN) was constructed, comprising 32 hubs, and these 19 identified upstream regulators (Figure 5).

### 3.5. Prognostic Markers

The analysis of 34 hub genes unveiled their prognostic impact on HNSCC. Notably, the upregulation of SERPINE1, PLAUR, THBS1, and ITGA6 was significantly associated with poor prognoses in HNSCC patients. SERPINE1 emerged as the most notable negative prognostic marker, exhibiting an HR of 1.5 and an HR *p*-value of 0.0025. Conversely, the overexpression of CALML5 and SPINK5 was significantly correlated with a favorable prognosis in HNSCC patients. CALML5 was the most significant positive prognostic marker, with an HR of 0.56 and an HR *p*-value of 0.000027. Intriguingly, SERPINE1, PLAUR, and THBS1 combined constituted the most significant adverse prognostic signature, with an HR of 1.8 and an HR *p*-value of 0.000013 (Table 3 and Supporting Information S2: Figure 1).

### 3.6. Boxplot Analysis

**Figure 6:**
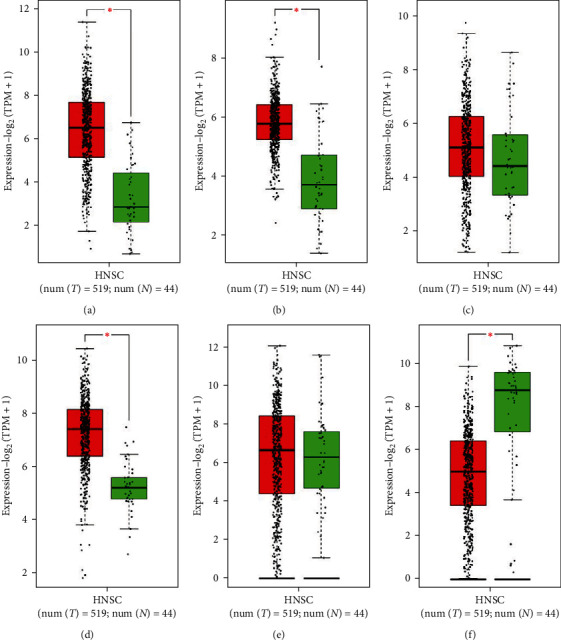
Gene expression analysis at the mRNA level using the GEPIA2 database for prognostic markers in HNSCC including (a) SERPINE1, (b) PLAUR, (c) THBS1, (d) ITGA6, (e) CALML5, and (f) SPINK5. Box plots are based on 519 cancerous tissues (red color) and 44 healthy samples (green color). HNSCC, head and neck squamous cell carcinoma. Outliers are marked by asterisks ( ^*∗*^).

The boxplot analysis revealed higher SERPINE1, PLAUR, THBS1, and ITGA6 mRNA expression levels in HNSCC tissues compared to healthy control samples. Conversely, SPINK5 exhibited lower expression in HNSCC tissues than in healthy controls, aligning with the study's findings. Notably, CALML5 displayed an upregulated pattern in oral dysplasia compared with normal oral tissue, and it exhibited under-expression in early OSCC compared to oral dysplasia. While the boxplot analysis did not indicate significant changes in CALML5 mRNA expression levels in HNSCC compared to healthy controls, the overall pattern seems consistent with the study's findings (Figure 6).

### 3.7. RNA-Sequencing

**Figure 7:**
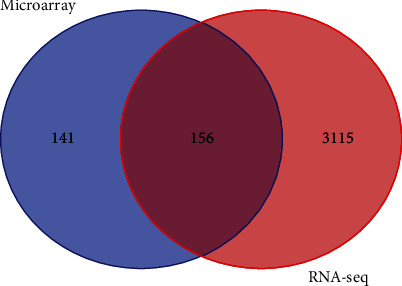
Venn diagram depicting the overlap of DEGs identified in microarray dataset GSE30784 and RNA-seq dataset GSE186775. DEG, differentially expressed gene.

Our analysis of the independent RNA-seq dataset GSE186775 using the GEO2R analysis tool revealed a total of 3271 DEGs in OSCC tissues compared to healthy controls (FDR < 0.05 and |log_2_ FC| > 1) (Supporting Information S1: Table 3). These DEGs included 1506 upregulated and 1765 downregulated genes. Notably, 156 genes exhibited overlap between the DEGs identified in the microarray dataset GSE30784 and the RNA-seq dataset GSE186775 (Figure 7 and Table 4). This overlap strengthens the validity of our findings.

### 3.8. Experimental Validation

Examination of microarray dataset GSE30784 uncovered heightened SERPINE1 and ITGA6 transcripts expression in oral dysplasia compared to healthy controls, with further elevation observed in primary OSCC relative to dysplastic tissues. Corroboration using RNA-seq dataset GSE186775 affirmed the upregulation of these markers in OSCC samples. Moreover, boxplot analysis exhibited increased mRNA levels of these genes in HNSCC tissues compared to normal specimens. Additionally, elevated expression of these genes was associated with unfavorable outcomes in HNSCC patients. These observations suggested SERPINE1 and ITGA6 as viable candidates for IHC evaluation.

**Figure 8:**
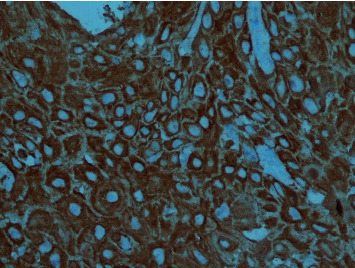
The expression of ITGA6 protein in primary oral squamous cell carcinoma (magnification: ×400).

Since SERPINE1 protein expression had been previously investigated [37], ITGA6 was selected for subsequent experimental validation. The findings confirmed a statistically significant increase (*p*-value = 0.007) in ITGA6 tissue expression within primary OSCC compared to healthy oral mucosa, corroborating our earlier results. Data on the mean percentage of ITGA6 expression in each group are summarized in Table 5. Semiquantitative assessments for ITGA6 are presented in Table 6.  Figure 8 illustrates a microphotograph of ITGA6 expression in primary OSCC.

## 4. Discussion

OSCC stands out as the most prevalent cancer type in the head and neck region, characterized by its aggressive nature [30]. The 5-year survival rate for patients with metastatic OSCC ranges from 40.1% to 66.8% [44]. Identifying signaling pathways, essential genes, and prognostic markers contributing to the malignant transformation from normal oral tissue to OPL and primary OSCC holds the promise of formulating the most effective strategies for treating individuals diagnosed with dysplasia or early-stage OSCC.

Following functional enrichment analysis, it was discerned that “Keratinization” emerged as one of the most significant pathways (R-HSA-6805567) and BPs (GO: 0031424) implicated in the malignant transformation from normal oral tissue to dysplasia and early OSCC. Additionally, “Keratin filament” was one of the most significant CC categories that was enriched in the development of primary OSCC. Interestingly, Jun et al. [45] reported a significant association between keratinization and an unfavorable outcome in patients with oral cancer. Their study, comprising nine patients with keratinized tumors and 8 with nonkeratinized tumors, demonstrated a poor prognosis in those with keratinized tumors compared to their nonkeratinized counterparts (*p*-value = 0.033). Conversely, Wolfer et al. [46] conducted a study assessing the impact of keratinization on the outcome of OSCC patients. Involving 151 patients, including 119 with no or low degrees of keratinization and 32 with medium or high grades, the results indicated that tumor recurrence was more evident in the group with low keratinization compared to the group with a high degree of keratinization (*p*-value = 0.0008).

In the current investigation, we utilized a microarray dataset GSE30784, which provided valuable insights into gene expression profile alterations during the transition from normal oral tissue to OPL and subsequently to primary OSCC. While microarray technology has been a helpful tool for gene expression profiling, it has well-documented limitations in dynamic range, sensitivity, and accuracy compared to RNA-seq [47–51]. To address these limitations and reinforce the validity of our findings, we conducted a cross-validation analysis using RNA-sequencing data from the GEO dataset GSE186775. This dataset identified DEGs in OSCC and compared them with healthy oral tissues. Subsequently, a set of common DEGs was identified between the microarray dataset GSE30784 and the RNA-seq dataset GSE186775. The overlap in DEGs (*n* = 156) between the two platforms provided support for the key findings from the microarray analysis, suggesting the involvement of these genes (such as SERPINE1, ITGA6, CALML5, and SPINK5) in the malignant transformation process. However, it is crucial to acknowledge that RNA-seq might not capture some genes identified through microarray analysis due to inherent platform limitations. Further investigations utilizing other high-throughput technologies, such as RNA-seq, are warranted to characterize the transcriptomic landscape of oral dysplasia and OSCC comprehensively.

The current investigation identified that the upregulation of SERPINE1, PLAUR, THBS1, and ITGA6 is significantly associated with poor prognoses in patients with HNSCC. Conversely, patients with HNSCC who exhibited overexpression of CALML5 and SPINK5 demonstrated a favorable prognosis, as evidenced by the log-rank test and HR *p*-values < 0.05. Recognizing that a single gene may not be sufficiently accurate for prognosis, a combination of markers was evaluated [52] to determine whether the HR is enhanced. The analysis revealed that the signature of three genes, SERPINE1, PLAUR, and THBS1, constituted the most compelling prognostic panel for HNSCC, demonstrating an HR of 1.8 and a log-rank test *p*-value of 0.000013. This combination of genes emerged as the best predictor for the prognosis of patients with HNSCC.

**Figure 9:**
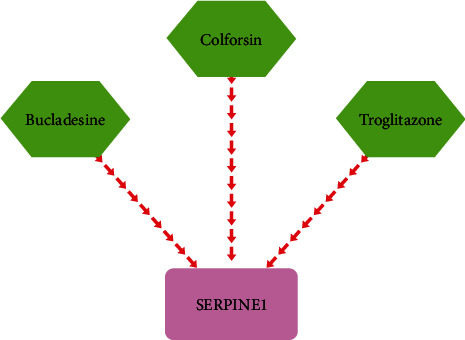
Visualization of the gene–drug interaction involving SERPINE1 sourced from the DrugBank database.

Serpin family E member 1 (SERPINE1) plays a role in several crucial BPs, including coagulation, homeostasis, and fibrinolysis [53, 54]. Previous studies have consistently confirmed the oncogenic role of SERPINE1 in various cancers [55]. For instance, it has been reported that SERPINE1 is overexpressed in gastric adenocarcinoma [56] and is associated with the metastasis of breast cancer cells [57]. In the context of OSCC, Zhao et al. [12] detected SERPINE1 in OSCC tissues and reported a positive correlation between SERPINE1 overexpression and advanced tumor stage. The same study demonstrated that the expression of SERPINE1 could be reduced via miR-617, resulting in decreased cell proliferation and increased apoptosis of OSCC cells. The authors concluded that the miR-617/SERPINE1 axis could be targeted for treating OSCC. Consistent with these findings, our study revealed that SERPINE1 was significantly overexpressed in dysplasia compared to normal oral mucosa (fold change = 7.01) and in early OSCC tissues compared with dysplasia (fold change = 10.06). This upregulation of SERPINE1 was further validated at the mRNA level using the GEPIA2 database. Additionally, information from the DrugBank database (accessible at https://go.drugbank.com/ [58]) indicates that Bucladesine and Colforsin function as inhibitors of SERPINE1, while Troglitazone acts as an antagonist for the enzyme (Figure 9). These insights into the molecular regulation of SERPINE1 provide potential avenues for therapeutic intervention in OSCC.

Serpin B5 (also recognized as maspin) represents an atypical, noninhibitory member of the serine protease inhibitor (serpin) superfamily [59, 60]. The functional role of the maspin gene is contingent upon both tissue specificity and the subcellular localization of the maspin protein [61, 62]. While extensively studied in breast carcinomas and colorectal cancer (CRC), maspin expression has been identified in various carcinomas, including those affecting the oral cavity, esophagus, stomach, larynx, lung, pancreas, thyroid, prostate, ovary, and urinary bladder [63]. In gastrointestinal cancers, cytoplasmic maspin positivity in gastric carcinomas and CRC indicates a low metastatic risk and late recurrence. In contrast, nuclear positivity correlates with early recurrence postsurgery, particularly in advanced-stage carcinomas [64–66]. Nuclear maspin expression in early stages may be associated with a higher risk of lymph node metastases [67]. Reduced maspin levels elevate the risk of tumor progression and the development of distant metastases [62, 65].

Banias et al. [64] assessed maspin expression in CRC and its potential role in quantifying tumor budding. In a prospective analysis of 49 CRC cases, categorized into low budding (group A, <5 buds) and high budding (group B, ≥5 buds), maspin expression in the tumor core and buds was evaluated in microsatellite stable adenocarcinomas. The study revealed higher pT and pN stages and infiltrative aspects in group B. Although no correlation was found between maspin expression in the tumor core and budding grade, cytoplasm-to-nuclear translocation of maspin was more frequent in group B. Banias et al. [44] suggested that the infiltrative aspect and nuclear maspin expression in buds may indicate lymph node metastases and contribute to a negative prognosis in CRC.

Bereczki et al. [68] investigated actin cytoskeleton disruption mechanisms in non-oropharyngeal OSCC and their correlation with high-risk human papillomavirus (HPV). Immunohistochemistry was employed in 43 surgically resected OSCC cases to evaluate various proteins, including maspin. The study suggested that cytoskeleton activity in nonoropharyngeal OSCC might be influenced by the Mena/E-cadherin/SMA axis, indicating active epithelial–mesenchymal interaction. High Mena expression was associated with poorly differentiated carcinomas, high-risk HPV, and an unfavorable prognosis, suggesting a potential role for maspin in the evolution of OSCC. The investigation identified independent adverse prognostic factors, such as high Mena expression, loss of E-cadherin, high SMA expression, and the presence of high-risk HPV, emphasizing the significance of maspin and other proteins in the prognosis of nonoropharyngeal OSCC with HPV involvement.

The urokinase plasminogen activator surface receptor (PLAUR), also known as uPAR, serves as a cancer-specific biomarker and plays a pivotal role in the migration and invasion of tumor cells [69, 70, and 71]. In cancer cells, PLAUR binds to its ligand (PLAU), initiating molecular changes in the tumor's extracellular matrix, ultimately resulting in invasive behavior and metastasis of tumor cells [72]. Numerous reports have highlighted the upregulation of PLAUR in various carcinomas, including gastric cancer [73] and CRC [74]. Consistent with these observations, the present study revealed an enhanced expression of PLAUR in dysplasia and primary OSCC tissues compared to healthy oral tissues (fold change = 2.91) and precancerous specimens (fold change = 4.47), respectively. This upregulation of PLAUR was further validated at the mRNA level using the GEPIA2 database. The findings underscore the potential significance of PLAUR as a molecular marker associated with the progression of oral lesions toward OSCC.

Pal et al. [75] conducted a study to elucidate the role of Thrombospondin 1 (THBS1) in the etiology of OSCC. In their investigation, THBS1 expression was assessed using IHC analysis and cDNA microarray in OSCC. The study demonstrated that THBS1 was overexpressed in the tumor microenvironment of OSCC cell lines, including HSC3 and HO1N1 cells. Furthermore, THBS1 expression in OSCC cell lines was induced by transforming growth factor-beta 1 (TGFB1), leading to enhanced invasion and migration of OSCC cells. The findings also revealed a positive correlation between THBS1 expression and the expression of several matrix metalloproteinases (MMPs), including MMP-3, MMP-11, and MMP-13, in HO1N1 cells. In line with these insights, our previous report [76] identified THBS1 as a hub gene in a PPI network based on the targets of differentially expressed miRNAs in primary OSCC tissues compared with their adjacent non-tumor epithelium. The current study corroborates these findings by demonstrating that THBS1 is upregulated in precancerous tissue compared with normal oral mucosa (fold change = 2.89) and in early OSCC specimens compared to dysplasia (fold change = 3.76). This upregulation of THBS1 was validated at both the mRNA and protein levels using the GEPIA2 and HPA databases, respectively. The collective evidence underscores the potential involvement of THBS1 in the early stages of OSCC development.

The current investigation identified the nuclear factor 1B-type (NFIB) as the most significant TF regulating the expression of 10 hub genes, displaying an NES of 5.365. Accumulating evidence suggests that NFIB acts as an upregulated oncogene in various cancers [77, 78], including triple-negative breast cancer [79], submandibular gland carcinoma [80], giant cell tumors of bone [81], and squamous cell carcinoma of the esophagus [82]. In contrast, Lou et al. [64] reported that NFIB functions as a tumor suppressor gene in oral cancer. Their study demonstrated that NFIB is the target of miR-626, wherein miR-626 suppresses NFIB expression by binding to the 3′-UTR of the gene, consequently contributing to oral cancer progression. Accordingly, the authors proposed that exosomal miR-626 could be a potential therapeutic target in treating oral cancer. This apparent dual role of NFIB in different cancer contexts highlights the complexity of its regulatory functions. It emphasizes the need to explore its specific roles in oral cancer further.

## 5. Conclusion

This study used bioinformatics to explore the molecular underpinnings of oral tissue progressing to dysplasia and early OSCC. A network analysis revealed 34 essential genes potentially involved, with functions related to skin development and keratinization. Interestingly, some genes (SERPINE1, PLAUR, THBS1, and ITGA6) correlated with poor patient outcomes, while others (CALML5, SPINK5) had the opposite effect. Notably, a combination of three genes (SERPINE1, PLAUR, and THBS1) emerged as a strong indicator of poor prognosis. Additionally, NFIB was pinpointed as crucial for regulating these essential genes. Finally, lab tests confirmed the overexpression of ITGA6 in OSCC samples, supporting the computational findings. This study provides valuable insights into the molecular signatures of early OSCC development, potentially paving the way for improved diagnosis and treatment strategies.

## Figures and Tables

**Table 1:** A total of 34 nodes were assigned as hub genes in the PPI network mediating OSCC progression.

Gene symbol	Degree	Betweenness
FN1	54	0.300479
CDSN	28	0.036372
DSG1	26	0.086235
CXCL12	26	0.033531
TGFB1	25	0.071694
LOR	25	0.064438
THBS1	23	0.023401
IGF1	22	0.060233
EVPL	21	0.058264
SERPINE1	21	0.028979
CXCL10	20	0.042468
MET	19	0.092324
STAT1	19	0.065658
KRT1	18	0.064641
ITGA6	17	0.076581
SPINK5	17	0.022736
SOX9	16	0.052818
ARG1	15	0.047812
LCE3D	15	0.024822
PLAUR	14	0.051421
BDNF	14	0.033030
KLK7	13	0.047707
KRT10	13	0.040765
KRT18	12	0.113795
PKP2	11	0.038212
LAMA3	11	0.022400
CLU	10	0.027848
SCEL	10	0.024702
ALOX12B	8	0.030863
NOX4	7	0.041693
F3	7	0.030884
GREM1	7	0.026074
CALML5	7	0.022591
PTGS1	6	0.040195

Abbreviations: OSCC, oral squamous cell carcinoma; PPI, protein–protein interaction network.

**Table 2:** Nineteen transcription factors were identified as upstream regulators of hub genes.

TF	NES	No. of targets	Targets
NFIB	5.365	10	FN1, SOX9, THBS1, SERPINE1, ITGA6, GREM1, CXCL12, IGF1, BDNF, PKP2
DDX4	5.332	12	BDNF, FN1, MET, IGF1, DSG1, SOX9, NOX4, KRT10, SERPINE1, F3, ITGA6, THBS1
PRKAA2	4.945	14	FN1, ARG1, SOX9, LAMA3, PTGS1, BDNF, ITGA6, IGF1, F3, DSG1, NOX4, SPINK5, GREM1, SCEL
SPATS2	4.822	10	GREM1, SOX9, BDNF, FN1, IGF1, MET, ITGA6, NOX4, DSG1, STAT1
NFE2	4.283	14	LAMA3, CLU, MET, F3, IGF1, NOX4, ITGA6, SERPINE1, KRT10, SCEL, PLAUR, LOR, KRT18, DSG1
MYB	4.242	12	EVPL, MET, BDNF, ITGA6, PTGS1, LAMA3, CXCL12, F3, KRT10, NOX4, GREM1, SCEL
EP300	4.23	10	SERPINE1, STAT1, KRT18, SOX9, CLU, KRT10, THBS1, BDNF, FN1, TGFB1
PARP1	4.134	17	FN1, BDNF, SOX9, MET, IGF1, SCEL, CXCL12, GREM1, SPINK5, NOX4, ITGA6, PTGS1, THBS1, F3, SERPINE1, DSG1, STAT1
HP1BP3	4.019	15	FN1, BDNF, SOX9, IGF1, MET, THBS1, NOX4, ITGA6, SERPINE1, SPINK5, DSG1, F3, STAT1, GREM1, PTGS1
IRF1	3.771	15	FN1, STAT1, BDNF, ITGA6, F3, MET, SOX9, CXCL10, SCEL, GREM1, IGF1, KRT10, THBS1, LOR, LAMA3
MYCN	3.747	16	BDNF, F3, THBS1, SOX9, ITGA6, IGF1, MET, SCEL, FN1, SERPINE1, SPINK5, GREM1, NOX4, CXCL12, LAMA3, CALML5
TAL1	3.658	12	IGF1, PKP2, BDNF, SERPINE1, MET, THBS1, FN1, NOX4, SCEL, SPINK5, EVPL, F3
GATA3	3.566	16	LAMA3, THBS1, KRT18, CLU, SERPINE1, SOX9, PKP2, STAT1, BDNF, ITGA6, SCEL, IGF1, FN1, NOX4, MET, KRT1
NFIC	3.555	12	SERPINE1, SOX9, THBS1, CLU, SCEL, CDSN, BDNF, KRT18, PTGS1, ITGA6, CXCL10, LAMA3
TCF12	3.503	15	SERPINE1, SOX9, THBS1, BDNF, CLU, IGF1, NOX4, CDSN, MET, KRT18, SCEL, FN1, STAT1, F3, LAMA3
E2F3	3.468	10	IGF1, FN1, ITGA6, SOX9, THBS1, BDNF, CXCL12, LAMA3, STAT1, MET
MAFK	3.271	14	MET, SERPINE1, BDNF, F3, SOX9, FN1, ITGA6, NOX4, SPINK5, GREM1, DSG1, EVPL, IGF1, SCEL
SMC1A	3.264	11	THBS1, MET, PKP2, ALOX12B, ITGA6, SOX9, BDNF, KRT18, CXCL12, CLU, LAMA3
KDM4E	3.095	11	IGF1, SOX9, MET, F3, SERPINE1, LAMA3, NOX4, GREM1, BDNF, ITGA6, DSG1

Abbreviation: NES, normalized enrichment score.

**Table 3:** A total of six hub genes in the PPI network associated with the OSCC development demonstrated prognostic impact in HNSCC.

Gene symbol (gene label)	HR (high)		*p* (log-rank test)	*p* (HR)	FC (dysplasia/normal)	FC (OSCC/dysplasia)
A, Single-gene						
SERPINE1 (A)	1.5		0.0024	0.0025	7.01	10.06
PLAUR (B)	1.4		0.0086	0.0092	2.91	4.47
THBS1 (C)	1.4		0.0099	0.01	2.89	3.76
ITGA6 (D)	1.3		0.035	0.036	2.45	2.23
CALML5 (E)	0.56		0.000027	0.000035	6.11	0.15
SPINK5 (F)	0.69		0.0062	0.0067	0.29	0.17

**Prognostic panel**	**HR (high)**	***p* (log-rank test)**		***p* (HR)**		

B, Combination of genes						
A + B + C	1.8	0.000013		0.000017		
A + C	1.8	0.00003		0.000038		
A + B + C + D	1.7	0.00025		0.00029		
A + D	1.6	0.00031		0.00035		
A + C + D	1.6	0.00031		0.00035		
A + B	1.6	0.00045		0.0005		
B + C + D	1.5	0.0025		0.0027		
C + D	1.5	0.0046		0.0049		
B + D	1.4	0.015		0.016		
E + F	0.69	0.0059		0.0064		

Abbreviations: HNSCC, head and neck squamous cell carcinoma; OSCC, oral squamous cell carcinoma; PPI, protein–protein interaction network.

**Table 4:** A total of 156 common DEGs in OSCC tissues compared to healthy control samples were identified by analyzing the microarray dataset GSE30784 and the RNA-seq dataset GSE186775.

Datasets	Total	Genes
The primary dataset (microarray, GSE30784) and the dataset used for cross-validation (RNA-seq, GSE186775)	156	ADAM12, **IGF1**, CPXM2, **MET**, ENDOU, CMPK2, ASPRV1, **KRT1**, GDF10, IRS1, HLF, **FN1**, SORBS2, **SPINK5**, **CDSN**, PPL, CYP3A5, PI15, TMPRSS13, SH3BGRL2, C1QTNF7, **ITGA6**, EPSTI1, RBP7, DSC1, HOTAIRM1, AIF1L, TGFB1, PGLYRP4, SPTLC3, SOCS3, ABCA12, **LAMA3**, KRT78, GPD1L, MMP1, **STAT1**, MMP12, REEP1, GADD45B, CDH3, GREM2, COL4A2, KRT9, **CALML5**, CGNL1, ALOXE3, PNPLA3, SASH1, DKK3, KLK7, CDON, LYVE1, DCBLD1, P3H2, DIO2, MTHFD1L, PLA2G7, MFAP2, SUSD4, ZSCAN31, PEG3, **CXCL10**, IL36RN, ADAP2, LAMC2, LYZ, NDRG4, NOVA1, WFDC12, CALD1, COL11A1, MMP10, SLC16A1, CYP4B1, COL12A1, TMPRSS11B, KIF26B, CXCL14, PLAU, EPB41L4A, KLK14, HOMER3, FPR3, AKR1B10, NOS1, CLDN11, WFDC5, TMEM45A, KRT13, PTHLH, NRP2, MAL, PTGFR, RIMS3, FCER1A, FAM167A, PPP2R2C, CRLF1, ABI3BP, HPGD, **GREM1**, TNFRSF12A, EPHX2, DUXAP10, HMGA2, ISG15, PDPN, RGS5, TNC, TUBB2A, MGLL, FAP, INHBA, **KLF7**, IFI6, LOXL2, SLC16A6, SLC15A1, **LCE3D**, **CLU**, **CXCL12**, RDH12, DLX3, MICAL2, COL14A1, CXCL9, DLX5, ANKRD29, CRNN, FAM43A, LYPD5, SLC47A2, **ALOX12B**, PAIP2B, APCDD1, IFI35, **SERPINE1**, RSAD2, ADH1B, KLK10, MYRIP, CAB39L, **KRT10**, PPP1R3C, RRAGD, TCEA3, TDO2, **PTGS1**, PMEPA1, MAMDC2, NRG1, FBXO32, **TGFBI**, VSIG10, GALNT16

*Note:* The bolded genes were indicated as hubs in the PPI network constructed based on the DEGs indicated by the microarray dataset GSE30784.

Abbreviation: DEG, differentially expressed gene.

**Table 5:** Mean percentage of ITGA6 in the studied groups.

Group	*n*	Mean ± SD	Minimum	Maximum
Primary OSCC	16	46.56 ± 19.43	8	77
Healthy oral mucosa	16	28.31 ± 15.49	5	59

Abbreviation: OSCC, oral squamous cell carcinoma.

**Table 6:** Semiquantitative results for ITGA6 in the studied groups.

Group	Negative	Weak	Moderate	Strong	Total
Primary OSCC	1	2	8	5	16
Healthy oral mucosa	2	8	4	2	16

Abbreviation: OSCC, oral squamous cell carcinoma.

## Data Availability

The datasets used and/or analyzed during the current study are available from the corresponding author upon reasonable request.

## Supporting Information

Supporting Information S1: Table 1. A total of 1248 DEGs were identified in oral dysplasia compared to the normal oral tissue. Table 2. A total of 947 DEGs were identified between primary oral squamous cell carcinoma and oral dysplasia. Table 3. A total of 3271 DEGs were identified between oral squamous cell carcinoma and oral healthy tissue based on the RNA-sequencing dataset GSE186775.

Supporting Information S2: Figure 1. Kaplan–Meier plots demonstrating the correlation between the expression of (A) SERPINE1, (B) PLAUR, (C) THBS1, (D) ITGA6, (E) CALML5, (F) SPINK5 and survival rate in HNSCC. Red and blue lines exhibit upregulated and downregulated genes, respectively. The *x*-axis and *y*-axis show the survival time of HNSCC patients and the percent of survival, respectively. The 95% confidence interval is shown with dotted lines. HNSCC, head and neck squamous cell carcinoma; HR, hazard ratio.
