# Supplementary material for: Malignant Transformation of Normal Oral Tissue to Dysplasia and Early Oral Squamous Cell Carcinoma: An In Silico Transcriptomics Approach
Source: Anal Cell Pathol (Amst). 2024 Sep 18;2024:6260651. doi: 10.1155/2024/6260651 (PMC11458300; doi:10.1155/2024/6260651)
Supplement: Supporting Information S1: — Table 1. A total of 1248 DEGs were identified in oral dysplasia compared to the normal oral tissue. Table 2. A total of 947 DEGs were identified between primary oral squamous cell carcinoma and oral dysplasia. Table 3. A total of 3271 DEGs were identified between oral squamous cell carcinoma and oral healthy tissue based on the RNA-sequencing dataset GSE186775. [file 6260651.f1.docx]

**Supplementary Table 1.** A total of 1248 DEGs were identified in oral dysplasia compared to the normal oral tissue.

| **Gene symbol** | **FDR** | **log2 FC** |
| --- | --- | --- |
| SPRR2G | 1.30E-12 | 7.63 |
| KRT1 | 1.62E-07 | 7.38 |
| CDSN | 1.67E-19 | 6.08 |
| LOR | 4.06E-07 | 5.08 |
| DSC1 | 5.59E-09 | 5.02 |
| KRT17 | 1.13E-12 | 5 |
| S100A7A | 1.05E-07 | 4.97 |
| KRT16 | 2.64E-08 | 4.91 |
| ASPRV1 | 2.79E-10 | 4.89 |
| COL1A1 | 1.74E-17 | 4.89 |
| LCE2B | 7.70E-11 | 4.85 |
| WFDC12 | 2.08E-10 | 4.79 |
| LCE3D | 9.99E-07 | 4.78 |
| SPINK6 | 1.94E-09 | 4.77 |
| BPIFC | 1.33E-15 | 4.75 |
| ADAMTS2 | 1.94E-18 | 4.71 |
| ALOX12B | 1.66E-10 | 4.69 |
| S100A7 | 5.53E-05 | 4.61 |
| KRT76 | 4.85E-07 | 4.6 |
| COMP | 6.30E-14 | 4.53 |
| LRRC15 | 3.40E-16 | 4.41 |
| ABCA12 | 3.36E-12 | 4.39 |
| IL36G | 7.35E-07 | 4.34 |
| PRR9 | 3.72E-10 | 4.23 |
| TMEM45A | 1.44E-09 | 4.23 |
| KRT2 | 3.17E-06 | 4.14 |
| LOX | 3.48E-15 | 4.06 |
| NEFL | 3.00E-07 | 4.05 |
| MMP1 | 3.36E-07 | 4.01 |
| COL1A2 | 2.59E-17 | 3.99 |
| ASPN | 1.21E-12 | 3.98 |
| DEFB4B | 7.76E-04 | 3.94 |
| FLG2 | 1.10E-04 | 3.94 |
| WDR66 | 3.78E-13 | 3.89 |
| POSTN | 2.05E-08 | 3.87 |
| COL5A1 | 2.21E-14 | 3.72 |
| EPPK1 | 6.03E-10 | 3.7 |
| COL8A1 | 8.96E-13 | 3.61 |
| HAL | 6.76E-06 | 3.55 |
| ADAM12 | 7.43E-11 | 3.53 |
| KRT75 | 6.14E-09 | 3.53 |
| CTHRC1 | 4.04E-10 | 3.5 |
| DSG1 | 5.53E-05 | 3.46 |
| SERPINB4 | 3.52E-03 | 3.46 |
| GREM1 | 4.67E-10 | 3.4 |
| RPTN | 8.46E-04 | 3.4 |
| KRTDAP | 9.58E-04 | 3.37 |
| IFI6 | 8.97E-07 | 3.36 |
| TENM2 | 3.43E-11 | 3.36 |
| COL3A1 | 2.52E-17 | 3.35 |
| LCE1B | 5.39E-06 | 3.34 |
| RIMS3 | 2.02E-14 | 3.28 |
| WISP1 | 3.75E-10 | 3.27 |
| VCAN | 3.14E-08 | 3.26 |
| FAM89A | 2.23E-16 | 3.22 |
| COL5A2 | 4.90E-15 | 3.19 |
| HOXD10 | 5.73E-13 | 3.19 |
| THY1 | 1.29E-10 | 3.19 |
| CARD18 | 2.41E-07 | 3.17 |
| DLX5 | 1.23E-18 | 3.17 |
| KRT9 | 8.69E-05 | 3.16 |
| F2RL2 | 4.90E-15 | 3.15 |
| RGS20 | 1.38E-09 | 3.14 |
| COL4A1 | 1.75E-13 | 3.13 |
| LOXL2 | 1.63E-12 | 3.13 |
| NMRAL1P1 | 2.98E-09 | 3.12 |
| PNLIPRP3 | 2.22E-07 | 3.12 |
| WFDC5 | 1.08E-07 | 3.12 |
| FAP | 5.26E-10 | 3.04 |
| MMP12 | 4.99E-03 | 3 |
| AKR1B10 | 3.16E-06 | 2.99 |
| CXCL10 | 1.15E-03 | 2.99 |
| KRT10 | 5.57E-11 | 2.95 |
| COL6A3 | 1.35E-14 | 2.92 |
| AADACL2 | 4.68E-04 | 2.91 |
| CXCL14 | 9.15E-18 | 2.91 |
| OASL | 1.00E-08 | 2.87 |
| ARG1 | 1.70E-03 | 2.86 |
| CD36 | 5.12E-05 | 2.85 |
| SERPINH1 | 7.23E-12 | 2.81 |
| SEMA3C | 6.95E-07 | 2.79 |
| PSORS1C2 | 3.08E-07 | 2.75 |
| NSG1 | 1.45E-05 | 2.74 |
| HOXC6 | 3.36E-12 | 2.72 |
| COL4A2 | 2.04E-09 | 2.7 |
| SERPINE1 | 3.11E-09 | 2.68 |
| THBS2 | 3.37E-10 | 2.68 |
| ENAH | 1.35E-14 | 2.67 |
| PTHLH | 3.95E-05 | 2.67 |
| TNFAIP6 | 8.44E-07 | 2.64 |
| COL12A1 | 4.81E-07 | 2.63 |
| INHBA | 1.68E-05 | 2.62 |
| MAGEA6 | 3.22E-04 | 2.62 |
| CALML5 | 6.45E-04 | 2.61 |
| KLK5 | 4.20E-03 | 2.58 |
| MFAP2 | 1.87E-07 | 2.57 |
| NAV1 | 1.23E-09 | 2.57 |
| PXDN | 3.14E-08 | 2.57 |
| IFI27 | 2.11E-07 | 2.53 |
| HYAL1 | 1.36E-14 | 2.52 |
| PI3 | 5.26E-05 | 2.5 |
| THBS4 | 6.54E-03 | 2.5 |
| LYPD5 | 7.03E-07 | 2.48 |
| LUM | 2.70E-09 | 2.47 |
| SLC7A11 | 1.40E-05 | 2.46 |
| CDH11 | 1.30E-09 | 2.45 |
| MTCL1 | 1.96E-11 | 2.45 |
| SFRP4 | 4.08E-05 | 2.44 |
| SPARC | 1.33E-12 | 2.43 |
| NREP | 1.10E-09 | 2.42 |
| SDR9C7 | 9.91E-05 | 2.42 |
| SERPINB7 | 1.34E-03 | 2.42 |
| C10orf99 | 5.38E-07 | 2.39 |
| NDRG4 | 1.91E-09 | 2.39 |
| BNC1 | 2.66E-07 | 2.38 |
| COL11A1 | 2.28E-05 | 2.37 |
| SLC16A1 | 2.06E-07 | 2.36 |
| SLC39A6 | 4.45E-07 | 2.35 |
| ESYT3 | 7.64E-09 | 2.34 |
| PTGER3 | 4.12E-09 | 2.34 |
| SELE | 1.47E-04 | 2.34 |
| PLAU | 3.18E-08 | 2.32 |
| ISG15 | 6.77E-05 | 2.31 |
| CD177 | 1.02E-03 | 2.28 |
| FAM43A | 1.77E-07 | 2.28 |
| KLK9///KLK8 | 3.97E-08 | 2.28 |
| GJA1 | 4.36E-14 | 2.27 |
| PTPRZ1 | 7.17E-06 | 2.27 |
| SERPINB12 | 5.10E-06 | 2.26 |
| CHST2 | 3.98E-06 | 2.25 |
| CNTNAP2 | 3.75E-07 | 2.25 |
| PMEPA1 | 1.12E-07 | 2.24 |
| WDR72 | 1.36E-02 | 2.24 |
| CDH3 | 8.97E-07 | 2.23 |
| EGFL6 | 6.11E-06 | 2.22 |
| CYP27C1 | 5.81E-08 | 2.21 |
| NNMT | 2.54E-04 | 2.21 |
| ADAP2 | 2.41E-10 | 2.2 |
| CXCL9 | 1.97E-02 | 2.2 |
| BST2 | 3.07E-04 | 2.19 |
| EPB41L4B | 3.55E-07 | 2.19 |
| FLRT3 | 1.50E-04 | 2.19 |
| ZIC2 | 4.17E-09 | 2.19 |
| SLC6A14 | 8.94E-03 | 2.18 |
| SLC28A3 | 1.42E-05 | 2.17 |
| ALOXE3 | 5.74E-09 | 2.16 |
| ANKRD29 | 9.48E-08 | 2.16 |
| MYO1B | 1.25E-07 | 2.16 |
| RCAN1 | 1.12E-08 | 2.15 |
| HAS3 | 6.63E-06 | 2.14 |
| C12orf75 | 2.18E-07 | 2.13 |
| HMCN1 | 2.12E-08 | 2.13 |
| MYO5A | 6.98E-08 | 2.13 |
| COL6A1 | 4.19E-11 | 2.12 |
| CRISPLD2 | 2.56E-09 | 2.12 |
| MFHAS1 | 3.96E-07 | 2.12 |
| DLX2 | 4.51E-09 | 2.11 |
| FBLIM1 | 4.99E-11 | 2.11 |
| C1orf186 | 4.38E-12 | 2.1 |
| MMP2 | 9.13E-07 | 2.1 |
| IFI44 | 1.45E-05 | 2.09 |
| HTRA1 | 3.93E-06 | 2.07 |
| PCDHB14 | 8.64E-09 | 2.06 |
| PTGS2 | 4.51E-03 | 2.06 |
| SLC47A2 | 6.24E-09 | 2.06 |
| TNC | 7.36E-06 | 2.06 |
| NID2 | 1.19E-07 | 2.05 |
| OLFML2B | 6.11E-07 | 2.05 |
| SESN3 | 6.92E-09 | 2.05 |
| CLEC7A | 2.08E-04 | 2.04 |
| PPP1R14C | 3.60E-07 | 2.04 |
| SLC2A1 | 9.74E-09 | 2.04 |
| ADAM23 | 9.58E-08 | 2.03 |
| COL7A1 | 2.17E-10 | 2.03 |
| DLX3 | 1.45E-08 | 2.03 |
| PGLYRP4 | 1.43E-06 | 2.03 |
| CA2 | 2.40E-03 | 2.02 |
| CYP26B1 | 3.37E-10 | 2.02 |
| FNDC1 | 3.67E-05 | 2.02 |
| STAT1 | 1.55E-04 | 2.02 |
| COL4A5 | 9.42E-08 | 2.01 |
| BMP2 | 1.15E-05 | 1.99 |
| COL13A1 | 1.68E-09 | 1.99 |
| GPX2 | 1.86E-04 | 1.99 |
| ELOVL7 | 4.32E-09 | 1.97 |
| KHDC1L | 2.39E-06 | 1.97 |
| KLK7 | 2.62E-02 | 1.97 |
| COL27A1 | 4.24E-08 | 1.96 |
| DSC2 | 1.62E-03 | 1.96 |
| GDA | 1.78E-03 | 1.96 |
| ANGPTL2 | 1.95E-06 | 1.95 |
| BTG3 | 1.29E-12 | 1.95 |
| PGBD5 | 7.60E-06 | 1.95 |
| IGFL2 | 7.18E-05 | 1.93 |
| PKP1 | 3.20E-04 | 1.93 |
| SPON1 | 3.80E-05 | 1.93 |
| APCDD1 | 1.07E-06 | 1.92 |
| NOS1 | 9.15E-05 | 1.92 |
| WISP2 | 1.54E-03 | 1.91 |
| LAMC2 | 2.03E-05 | 1.9 |
| TYMP | 1.25E-06 | 1.9 |
| COL4A6 | 6.23E-10 | 1.89 |
| GPR68 | 3.93E-06 | 1.89 |
| SNCAIP | 1.86E-08 | 1.89 |
| DNM3OS | 7.20E-06 | 1.88 |
| FAM167A | 1.27E-06 | 1.88 |
| IFI44L | 5.79E-03 | 1.88 |
| NETO2 | 1.97E-05 | 1.88 |
| BGN | 2.89E-08 | 1.87 |
| EPSTI1 | 5.70E-03 | 1.87 |
| FBXO45 | 2.99E-08 | 1.87 |
| NIPAL4 | 2.06E-04 | 1.87 |
| PTGS1 | 5.67E-06 | 1.87 |
| KRT14 | 1.07E-06 | 1.86 |
| MMP10 | 3.41E-04 | 1.86 |
| SFRP2 | 1.42E-02 | 1.86 |
| LTBP1 | 3.56E-06 | 1.85 |
| PLA2G3 | 3.32E-06 | 1.84 |
| VNN1 | 7.91E-04 | 1.84 |
| MBD1 | 1.41E-06 | 1.83 |
| LY6G6C | 2.92E-03 | 1.82 |
| AKR1C1 | 1.15E-03 | 1.81 |
| SMTN | 5.06E-05 | 1.81 |
| APOL1 | 6.90E-06 | 1.8 |
| DDX58 | 5.67E-05 | 1.8 |
| ELMOD1 | 2.04E-04 | 1.8 |
| FEZ1 | 5.75E-07 | 1.8 |
| IL36RN | 5.93E-03 | 1.8 |
| MUCL1 | 2.76E-03 | 1.8 |
| PANX1 | 4.93E-10 | 1.8 |
| COL16A1 | 9.03E-13 | 1.79 |
| CSRP2 | 3.37E-10 | 1.79 |
| FBN2 | 3.64E-05 | 1.79 |
| ADGRV1 | 1.63E-05 | 1.78 |
| FCHSD1 | 1.23E-09 | 1.76 |
| IFFO2 | 7.21E-07 | 1.76 |
| MICAL2 | 3.16E-10 | 1.76 |
| SATB2 | 3.69E-11 | 1.76 |
| CPXM1 | 2.89E-06 | 1.75 |
| CTSC | 2.17E-09 | 1.75 |
| GALNT18 | 1.69E-08 | 1.75 |
| IGFBP3 | 2.35E-08 | 1.74 |
| SSFA2 | 1.53E-10 | 1.74 |
| XAF1 | 6.80E-06 | 1.74 |
| HOMER3 | 1.39E-08 | 1.73 |
| PCDH7 | 8.06E-04 | 1.72 |
| RSAD2 | 1.08E-02 | 1.72 |
| ELN | 2.00E-03 | 1.71 |
| IFIT1 | 3.05E-04 | 1.71 |
| RDH12 | 9.87E-03 | 1.7 |
| AATBC | 7.83E-07 | 1.69 |
| TUBB2A | 8.69E-07 | 1.69 |
| C21orf91 | 2.72E-06 | 1.68 |
| CRLF1 | 5.38E-07 | 1.68 |
| FNDC3B | 1.04E-04 | 1.68 |
| COL6A2 | 5.73E-05 | 1.67 |
| DUXAP10 | 1.15E-03 | 1.67 |
| SLC15A1 | 6.84E-05 | 1.67 |
| TPBG | 7.47E-14 | 1.67 |
| CCDC3 | 2.33E-05 | 1.66 |
| KLK10 | 1.40E-02 | 1.66 |
| LAMB1 | 8.11E-09 | 1.66 |
| SPAG17 | 4.06E-03 | 1.66 |
| ENPEP | 9.91E-06 | 1.65 |
| FAM83B | 6.21E-05 | 1.65 |
| FSCN1 | 4.01E-07 | 1.65 |
| OAS2 | 5.26E-04 | 1.65 |
| SIRPA | 5.33E-12 | 1.65 |
| AKR1C3 | 1.45E-03 | 1.64 |
| CDHR1 | 4.01E-05 | 1.64 |
| CTSK | 3.59E-07 | 1.64 |
| EGLN3 | 7.54E-05 | 1.64 |
| GPR176 | 2.62E-06 | 1.64 |
| KRT6B | 4.16E-03 | 1.64 |
| BBOX1 | 4.45E-06 | 1.63 |
| DUSP14 | 2.79E-05 | 1.63 |
| HAUS7 | 4.10E-09 | 1.62 |
| KLK14 | 9.61E-07 | 1.62 |
| LIPG | 1.37E-04 | 1.62 |
| PRSS23 | 3.04E-05 | 1.62 |
| RTP4 | 9.88E-04 | 1.62 |
| KCNJ15 | 1.04E-03 | 1.61 |
| PDPN | 2.05E-08 | 1.61 |
| PTGFRN | 2.20E-08 | 1.61 |
| SULF1 | 2.72E-05 | 1.61 |
| CNTN1 | 3.56E-06 | 1.59 |
| SLC7A8 | 5.32E-07 | 1.58 |
| TEAD4 | 2.06E-05 | 1.58 |
| TP63 | 2.40E-03 | 1.58 |
| FLVCR2 | 3.08E-06 | 1.57 |
| GDF10 | 1.78E-03 | 1.57 |
| GJC1 | 1.95E-08 | 1.57 |
| KIRREL | 1.31E-08 | 1.57 |
| NRCAM | 6.85E-06 | 1.57 |
| PTGFR | 5.43E-04 | 1.57 |
| TMPRSS13 | 6.72E-06 | 1.57 |
| TRAM2 | 1.52E-04 | 1.57 |
| IFIT3 | 1.17E-03 | 1.56 |
| CPXM2 | 1.68E-05 | 1.55 |
| EDIL3 | 2.66E-04 | 1.54 |
| PLAUR | 7.38E-05 | 1.54 |
| SNAI2 | 3.88E-10 | 1.54 |
| CDKN2A | 1.09E-03 | 1.53 |
| MAP2 | 1.52E-03 | 1.53 |
| NELL2 | 2.22E-02 | 1.53 |
| P3H2 | 4.64E-03 | 1.53 |
| SLC38A5 | 8.55E-08 | 1.53 |
| THBS1 | 1.23E-02 | 1.53 |
| BMP1 | 2.56E-09 | 1.52 |
| GBP1 | 9.36E-04 | 1.52 |
| GNAI1 | 2.87E-08 | 1.52 |
| KLHDC7B | 4.54E-04 | 1.52 |
| AFAP1L1 | 1.01E-04 | 1.51 |
| AKR1C2 | 1.84E-03 | 1.51 |
| ANO1 | 2.53E-02 | 1.51 |
| BDNF | 6.12E-07 | 1.51 |
| ELAVL2 | 6.00E-05 | 1.51 |
| HERC5 | 4.32E-04 | 1.51 |
| JMY | 5.71E-10 | 1.51 |
| RCN3 | 6.73E-06 | 1.51 |
| C5orf46 | 3.35E-02 | 1.5 |
| CCND2 | 4.65E-04 | 1.5 |
| FPR3 | 2.81E-03 | 1.5 |
| PHLDB2 | 1.93E-08 | 1.5 |
| SERPINA12 | 1.02E-03 | 1.5 |
| COL14A1 | 2.26E-03 | 1.49 |
| FAT1 | 1.10E-07 | 1.49 |
| PGF | 6.50E-05 | 1.49 |
| PLCXD1 | 9.43E-06 | 1.49 |
| PPP2R3A | 3.56E-05 | 1.49 |
| NTRK2 | 2.36E-04 | 1.48 |
| PDGFRB | 9.28E-08 | 1.48 |
| AEBP1 | 3.05E-05 | 1.47 |
| BCL2L10 | 2.09E-05 | 1.47 |
| F2RL1 | 2.47E-04 | 1.47 |
| GPR153 | 1.56E-09 | 1.47 |
| GPX8 | 3.42E-05 | 1.47 |
| RNF217 | 3.81E-06 | 1.47 |
| SLAMF7 | 4.96E-02 | 1.47 |
| SLC44A1 | 4.81E-06 | 1.47 |
| TPSAB1 | 1.89E-03 | 1.47 |
| UCHL1 | 7.16E-03 | 1.47 |
| VANGL2 | 7.86E-08 | 1.47 |
| ZC3H12A | 7.67E-03 | 1.47 |
| ABCA13 | 2.66E-04 | 1.46 |
| MINPP1 | 5.94E-08 | 1.46 |
| MIR31HG | 1.65E-05 | 1.46 |
| CYP1B1 | 2.88E-02 | 1.45 |
| HENMT1 | 1.35E-05 | 1.45 |
| LAMA3 | 4.79E-06 | 1.45 |
| ADAMTS12 | 3.59E-06 | 1.44 |
| CDC25B | 2.53E-08 | 1.44 |
| HOXB7 | 1.88E-06 | 1.44 |
| LURAP1L | 7.68E-04 | 1.44 |
| NAPEPLD | 6.80E-07 | 1.44 |
| PNPLA3 | 5.11E-05 | 1.44 |
| ZSCAN31 | 1.11E-04 | 1.44 |
| CUX1 | 1.10E-06 | 1.43 |
| DLX1 | 7.31E-08 | 1.43 |
| KRT6C | 3.51E-03 | 1.43 |
| MCTP1 | 8.91E-04 | 1.43 |
| NID1 | 4.25E-05 | 1.43 |
| TDO2 | 1.45E-03 | 1.43 |
| FAM84A | 1.40E-04 | 1.42 |
| ATP2C1 | 7.79E-09 | 1.41 |
| CYP39A1 | 4.25E-06 | 1.41 |
| GATA3 | 4.27E-02 | 1.41 |
| MCAM | 2.72E-06 | 1.41 |
| FAM20C | 2.03E-06 | 1.4 |
| LAMP3 | 9.36E-03 | 1.4 |
| P4HA2 | 7.63E-12 | 1.4 |
| PCOLCE | 4.73E-04 | 1.4 |
| SH3PXD2B | 2.70E-08 | 1.4 |
| CCL8 | 6.92E-03 | 1.39 |
| DFNA5 | 2.05E-04 | 1.39 |
| PDK1 | 2.05E-02 | 1.39 |
| C6orf15 | 5.24E-05 | 1.38 |
| CDC14B | 1.94E-03 | 1.38 |
| CLIC4 | 1.15E-03 | 1.38 |
| LTB4R | 1.14E-03 | 1.38 |
| RAB7B | 4.97E-04 | 1.38 |
| EGR3 | 3.31E-02 | 1.37 |
| EML1 | 4.79E-04 | 1.37 |
| NRG1 | 1.46E-04 | 1.37 |
| PLAT | 9.66E-03 | 1.37 |
| SLC39A14 | 3.59E-06 | 1.37 |
| C1orf68 | 8.71E-04 | 1.36 |
| CEBPB | 3.64E-09 | 1.36 |
| MARCH3 | 1.26E-03 | 1.36 |
| RAB32 | 1.81E-08 | 1.36 |
| CD276 | 1.46E-08 | 1.35 |
| COL17A1 | 2.82E-03 | 1.35 |
| RAB31 | 5.81E-07 | 1.35 |
| TPSB2 | 2.95E-02 | 1.35 |
| DLGAP4 | 1.03E-13 | 1.34 |
| GAS1 | 9.77E-05 | 1.34 |
| TSPAN11 | 1.25E-06 | 1.34 |
| DEGS1 | 2.26E-06 | 1.33 |
| LTBP2 | 4.60E-06 | 1.33 |
| NLGN4X | 4.06E-05 | 1.33 |
| S100A12 | 8.95E-03 | 1.33 |
| TMEM158 | 1.09E-06 | 1.33 |
| TWIST2 | 7.25E-05 | 1.33 |
| ZNF532 | 7.81E-11 | 1.33 |
| ANGPT2 | 1.54E-06 | 1.32 |
| BCL11B | 3.36E-04 | 1.32 |
| CD274 | 3.43E-02 | 1.32 |
| CHODL | 2.72E-06 | 1.32 |
| ESRG | 2.97E-02 | 1.32 |
| GPSM1 | 2.71E-07 | 1.32 |
| LRRC8E | 2.15E-05 | 1.32 |
| NDE1 | 2.11E-07 | 1.32 |
| NTM | 1.79E-02 | 1.32 |
| THBD | 2.48E-06 | 1.32 |
| VNN3 | 1.28E-04 | 1.32 |
| CHN1 | 8.57E-05 | 1.31 |
| NDUFA4L2 | 3.91E-03 | 1.31 |
| ODC1 | 8.94E-03 | 1.31 |
| PRTFDC1 | 1.08E-05 | 1.31 |
| SOX4 | 2.04E-08 | 1.31 |
| ASTN2 | 1.22E-06 | 1.3 |
| BICD2 | 2.49E-05 | 1.3 |
| CPA3 | 1.28E-02 | 1.3 |
| DDX60 | 4.17E-04 | 1.3 |
| FOXN1 | 3.77E-03 | 1.3 |
| GPNMB | 1.00E-11 | 1.3 |
| ID3 | 6.93E-07 | 1.3 |
| IMPA2 | 1.74E-03 | 1.3 |
| LRP12 | 4.06E-05 | 1.3 |
| MTHFD1L | 1.77E-02 | 1.3 |
| NRP2 | 4.47E-06 | 1.3 |
| PCDHB9 | 1.13E-07 | 1.3 |
| ABCG1 | 1.01E-05 | 1.29 |
| ACKR4 | 1.97E-03 | 1.29 |
| ANTXR1 | 3.45E-09 | 1.29 |
| FABP5 | 5.00E-05 | 1.29 |
| FMNL2 | 4.39E-06 | 1.29 |
| FN1 | 5.30E-03 | 1.29 |
| ITGA6 | 6.94E-05 | 1.29 |
| KIAA1644 | 1.04E-08 | 1.29 |
| MXRA8 | 1.23E-03 | 1.29 |
| NAV3 | 8.48E-03 | 1.29 |
| PRSS12 | 3.00E-06 | 1.29 |
| SLC26A9 | 3.63E-03 | 1.29 |
| WDR53 | 2.55E-08 | 1.29 |
| GPR161 | 3.92E-12 | 1.28 |
| KCND3 | 9.43E-04 | 1.28 |
| PTPRD | 9.66E-05 | 1.28 |
| SOCS3 | 4.53E-02 | 1.28 |
| COPZ2 | 1.57E-05 | 1.27 |
| GABRE | 1.29E-03 | 1.27 |
| NOX4 | 2.69E-07 | 1.27 |
| PLA2G7 | 1.94E-02 | 1.27 |
| PROCR | 3.02E-06 | 1.27 |
| REEP1 | 4.80E-04 | 1.27 |
| SPTLC3 | 2.71E-03 | 1.27 |
| TAP1 | 3.78E-04 | 1.27 |
| TGFB3 | 1.73E-05 | 1.27 |
| CDKN1A | 1.13E-04 | 1.26 |
| CLEC2B | 3.36E-06 | 1.26 |
| FAM69A | 4.16E-06 | 1.26 |
| MAFB | 3.55E-09 | 1.26 |
| PECAM1 | 1.87E-04 | 1.26 |
| POLR3G | 7.35E-05 | 1.26 |
| ABCC1 | 3.65E-05 | 1.25 |
| CCL2 | 1.41E-02 | 1.25 |
| CMPK2 | 2.47E-02 | 1.25 |
| FOXRED2 | 9.03E-07 | 1.25 |
| PLEKHG4 | 1.88E-06 | 1.25 |
| TGFBI | 4.86E-07 | 1.25 |
| CHRNA9 | 6.58E-04 | 1.24 |
| COL15A1 | 4.78E-04 | 1.24 |
| GM2A | 1.11E-06 | 1.24 |
| SMOX | 1.83E-06 | 1.24 |
| DCBLD1 | 2.34E-07 | 1.23 |
| DKK3 | 2.72E-06 | 1.23 |
| GUCY1B3 | 1.97E-04 | 1.23 |
| HSD3B7 | 3.20E-05 | 1.23 |
| KIF26B | 1.36E-05 | 1.23 |
| MSX1 | 1.28E-04 | 1.23 |
| MX1 | 6.66E-04 | 1.23 |
| PLVAP | 6.68E-05 | 1.23 |
| COL6A6 | 2.05E-03 | 1.22 |
| DNMT3B | 3.74E-09 | 1.22 |
| PDE8A | 1.01E-05 | 1.22 |
| TNFRSF12A | 6.47E-03 | 1.22 |
| BNIP3 | 2.46E-04 | 1.21 |
| CRMP1 | 1.33E-04 | 1.21 |
| MSN | 3.42E-05 | 1.21 |
| MXRA5 | 1.02E-04 | 1.21 |
| TGFB1 | 4.31E-03 | 1.21 |
| ADORA2B | 8.50E-03 | 1.2 |
| AVPR1A | 1.72E-04 | 1.2 |
| CPA4 | 3.84E-02 | 1.2 |
| DSC3 | 7.42E-04 | 1.2 |
| GPLD1 | 1.73E-03 | 1.2 |
| SQLE | 5.18E-03 | 1.2 |
| ARRDC4 | 1.68E-05 | 1.19 |
| C1QTNF5 | 5.57E-05 | 1.19 |
| CEMIP | 2.35E-02 | 1.19 |
| EHD2 | 1.93E-04 | 1.19 |
| GOLGA7B | 1.37E-04 | 1.19 |
| PPIF | 2.04E-02 | 1.19 |
| RFTN1 | 1.74E-02 | 1.19 |
| SPRR4 | 5.62E-04 | 1.19 |
| TRIO | 3.81E-07 | 1.19 |
| TUBB2B | 9.36E-05 | 1.19 |
| ANPEP | 5.80E-03 | 1.18 |
| ATP1B3 | 1.10E-09 | 1.18 |
| CXCL12 | 2.45E-02 | 1.18 |
| GGH | 3.88E-05 | 1.18 |
| NMB | 1.22E-05 | 1.18 |
| ALDH1L2 | 3.69E-02 | 1.17 |
| ENTPD1 | 6.74E-03 | 1.17 |
| FADS1 | 2.77E-03 | 1.17 |
| IQCJ | 3.58E-03 | 1.17 |
| IRF9 | 5.11E-05 | 1.17 |
| LRRC8D | 2.44E-06 | 1.17 |
| MLF1 | 9.68E-03 | 1.17 |
| CD109 | 2.09E-05 | 1.16 |
| GALNT16 | 2.43E-03 | 1.16 |
| IFI35 | 3.49E-02 | 1.16 |
| KYNU | 4.71E-02 | 1.16 |
| MB21D2 | 5.97E-08 | 1.16 |
| OAS3 | 9.36E-04 | 1.16 |
| OSR2 | 1.97E-03 | 1.16 |
| PLPPR4 | 2.70E-04 | 1.16 |
| TNFAIP1 | 1.08E-07 | 1.16 |
| ARHGAP28 | 4.22E-05 | 1.15 |
| F2R | 1.14E-06 | 1.15 |
| HSPA2 | 3.76E-04 | 1.15 |
| KCNJ18 | 1.73E-03 | 1.15 |
| LAPTM4B | 3.47E-10 | 1.15 |
| LRRN1 | 2.71E-02 | 1.15 |
| P2RX7 | 3.22E-04 | 1.15 |
| PFN2 | 6.48E-03 | 1.15 |
| SPON2 | 7.51E-03 | 1.15 |
| C1QTNF2 | 2.24E-04 | 1.14 |
| CARD10 | 7.69E-03 | 1.14 |
| FAM65C | 1.17E-04 | 1.14 |
| GLI3 | 1.35E-07 | 1.14 |
| IRS1 | 2.73E-06 | 1.14 |
| MACF1 | 4.19E-11 | 1.14 |
| RBP1 | 3.04E-02 | 1.14 |
| CACNA2D3 | 3.85E-07 | 1.13 |
| DCUN1D3 | 1.69E-05 | 1.13 |
| DUXAP8 | 7.81E-05 | 1.13 |
| ID2B | 8.57E-05 | 1.13 |
| KRT6A | 1.96E-02 | 1.13 |
| PARP14 | 2.09E-03 | 1.13 |
| DSEL | 2.41E-04 | 1.12 |
| FSTL1 | 5.74E-04 | 1.12 |
| FUT1 | 1.61E-04 | 1.12 |
| KCTD15 | 2.78E-05 | 1.12 |
| NPNT | 3.29E-05 | 1.12 |
| OSMR | 6.89E-03 | 1.12 |
| PCDH17 | 4.19E-06 | 1.12 |
| PI15 | 2.01E-02 | 1.12 |
| UNC93A | 3.75E-02 | 1.12 |
| AMPD3 | 1.46E-04 | 1.11 |
| EN1 | 1.22E-05 | 1.11 |
| ENG | 1.94E-04 | 1.11 |
| HIST1H2BK | 8.65E-06 | 1.11 |
| IL13RA2 | 3.92E-02 | 1.11 |
| KCNJ8 | 3.34E-04 | 1.11 |
| MMP16 | 1.86E-03 | 1.11 |
| NLRC5 | 5.72E-03 | 1.11 |
| SCG2 | 2.98E-03 | 1.11 |
| STX17 | 8.80E-09 | 1.11 |
| TTC39B | 1.33E-04 | 1.11 |
| CYP24A1 | 2.37E-02 | 1.1 |
| DNM1 | 3.54E-03 | 1.1 |
| HPSE | 2.48E-04 | 1.1 |
| MFAP3L | 3.69E-02 | 1.1 |
| PPP2R2C | 1.32E-02 | 1.1 |
| SERPINE2 | 3.50E-03 | 1.1 |
| BICC1 | 4.38E-03 | 1.09 |
| COL18A1 | 3.91E-08 | 1.09 |
| DNASE1L2 | 4.90E-04 | 1.09 |
| IL12RB2 | 3.42E-04 | 1.09 |
| MARVELD1 | 1.04E-08 | 1.09 |
| MET | 4.31E-04 | 1.09 |
| MLLT11 | 5.85E-03 | 1.09 |
| MYO10 | 3.17E-05 | 1.09 |
| PIEZO2 | 8.04E-04 | 1.09 |
| TMEM200A | 4.02E-03 | 1.09 |
| TNS4 | 1.14E-05 | 1.09 |
| H2BFS | 1.12E-04 | 1.08 |
| HEPH | 1.98E-02 | 1.08 |
| HMGA2 | 1.13E-04 | 1.08 |
| IL37 | 9.55E-04 | 1.08 |
| LPAR3 | 3.20E-03 | 1.08 |
| MSANTD3 | 1.59E-06 | 1.08 |
| SPIRE1 | 4.19E-04 | 1.08 |
| TSHZ3 | 3.75E-04 | 1.08 |
| TUSC3 | 1.93E-03 | 1.08 |
| WISP3 | 8.11E-04 | 1.08 |
| ACP5 | 5.59E-04 | 1.07 |
| CCL11 | 4.82E-03 | 1.07 |
| IGFBP4 | 3.79E-03 | 1.07 |
| LAMA4 | 1.09E-04 | 1.07 |
| MAGEA10 | 5.87E-03 | 1.07 |
| PARP9 | 5.43E-03 | 1.07 |
| PCDHB16 | 1.83E-04 | 1.07 |
| PRKCDBP | 6.13E-04 | 1.07 |
| TPST1 | 1.02E-03 | 1.07 |
| WFDC1 | 8.83E-04 | 1.07 |
| CALD1 | 5.75E-03 | 1.06 |
| CAMSAP2 | 7.17E-04 | 1.06 |
| CAV1 | 2.25E-03 | 1.06 |
| CDON | 1.01E-03 | 1.06 |
| IL15RA | 2.86E-04 | 1.06 |
| LRP8 | 1.74E-03 | 1.06 |
| MAF | 5.02E-03 | 1.06 |
| POU3F1 | 2.63E-05 | 1.06 |
| PTK7 | 6.84E-08 | 1.06 |
| ZAK | 7.87E-05 | 1.06 |
| AFAP1L2 | 1.28E-04 | 1.05 |
| FLJ32255 | 4.32E-04 | 1.05 |
| GJA3 | 6.33E-04 | 1.05 |
| IGSF3 | 1.90E-04 | 1.05 |
| IRX4 | 1.15E-04 | 1.05 |
| MAGEA11 | 7.78E-03 | 1.05 |
| PLXDC1 | 9.69E-03 | 1.05 |
| PRRX1 | 1.41E-02 | 1.05 |
| RGS5 | 1.28E-02 | 1.05 |
| SERPINB8 | 1.03E-03 | 1.05 |
| SGCB | 8.24E-04 | 1.05 |
| TMEM54 | 1.32E-02 | 1.05 |
| APBB2 | 5.81E-05 | 1.04 |
| ASAP1 | 2.79E-05 | 1.04 |
| B3GNT9 | 1.71E-09 | 1.04 |
| BEX3 | 1.01E-08 | 1.04 |
| CD44 | 4.24E-03 | 1.04 |
| IDE | 2.93E-03 | 1.04 |
| IFITM2 | 8.31E-04 | 1.04 |
| IGF1 | 3.64E-02 | 1.04 |
| RGS3 | 1.10E-04 | 1.04 |
| STARD4 | 2.86E-02 | 1.04 |
| UBE2L6 | 9.88E-04 | 1.04 |
| CALU | 1.87E-03 | 1.03 |
| CASK | 1.82E-10 | 1.03 |
| HSD17B6 | 1.75E-06 | 1.03 |
| KLF7 | 1.63E-03 | 1.03 |
| NANOS1 | 2.09E-04 | 1.03 |
| NRIP1 | 4.36E-07 | 1.03 |
| SDCCAG8 | 2.04E-07 | 1.03 |
| SFXN3 | 5.32E-07 | 1.03 |
| TNN | 5.95E-04 | 1.03 |
| AAED1 | 2.87E-06 | 1.02 |
| C4orf48 | 2.44E-03 | 1.02 |
| CCDC102B | 3.57E-06 | 1.02 |
| ERVH-6 | 1.02E-03 | 1.02 |
| PGM2L1 | 4.31E-03 | 1.02 |
| PLOD3 | 1.71E-06 | 1.02 |
| RASIP1 | 5.15E-04 | 1.02 |
| RNF168 | 1.34E-08 | 1.02 |
| AP2B1 | 1.41E-02 | 1.01 |
| FLNB | 1.17E-04 | 1.01 |
| GALNT10 | 3.91E-03 | 1.01 |
| GDPD2 | 4.70E-05 | 1.01 |
| GNB5 | 5.19E-03 | 1.01 |
| HTRA3 | 4.36E-04 | 1.01 |
| ID1 | 1.95E-04 | 1.01 |
| ID2 | 2.07E-06 | 1.01 |
| MEOX1 | 2.01E-04 | 1.01 |
| PAPLN | 4.12E-02 | 1.01 |
| UCK2 | 5.88E-06 | 1.01 |
| CAPN1 | 2.31E-04 | -1.01 |
| CES2 | 2.22E-02 | -1.01 |
| CSTF2 | 1.86E-05 | -1.01 |
| FAAH2 | 1.87E-04 | -1.01 |
| GOLPH3L | 3.25E-07 | -1.01 |
| HOTAIRM1 | 2.52E-03 | -1.01 |
| TJP1 | 1.56E-03 | -1.01 |
| ZNRF1 | 4.47E-05 | -1.01 |
| BBIP1 | 1.81E-07 | -1.02 |
| CHP1 | 1.61E-02 | -1.02 |
| EPB41L1 | 4.02E-03 | -1.02 |
| EPB41L4A | 5.21E-08 | -1.02 |
| ESYT2 | 9.15E-04 | -1.02 |
| LPCAT4 | 2.32E-04 | -1.02 |
| MTMR10 | 1.14E-05 | -1.02 |
| NYNRIN | 1.08E-02 | -1.02 |
| PGD | 5.12E-03 | -1.02 |
| SASH1 | 3.53E-04 | -1.02 |
| SLC16A6 | 1.35E-02 | -1.02 |
| SLC6A1 | 1.44E-02 | -1.02 |
| TRIM2 | 8.33E-04 | -1.02 |
| ZNF880 | 3.16E-02 | -1.02 |
| ADGRD1 | 1.49E-02 | -1.03 |
| ANKRD35 | 3.28E-03 | -1.03 |
| BICDL2 | 2.31E-03 | -1.03 |
| EPB41L3 | 1.14E-02 | -1.03 |
| HSPB2 | 6.21E-03 | -1.03 |
| PKP2 | 2.65E-03 | -1.03 |
| RHOD | 1.90E-02 | -1.03 |
| SDPR | 1.23E-02 | -1.03 |
| TM4SF1 | 2.96E-03 | -1.03 |
| CAST | 3.51E-06 | -1.04 |
| GALNT7 | 1.15E-04 | -1.04 |
| KSR1 | 3.41E-08 | -1.04 |
| SUOX | 2.24E-06 | -1.04 |
| TCEA3 | 9.66E-03 | -1.04 |
| ECHDC2 | 2.52E-06 | -1.05 |
| MYB | 8.68E-05 | -1.05 |
| SDC4 | 6.92E-06 | -1.05 |
| TLE2 | 4.76E-03 | -1.05 |
| TMEM150C | 3.33E-04 | -1.05 |
| ADD3 | 5.56E-06 | -1.06 |
| MAML3 | 2.36E-04 | -1.06 |
| PDZD2 | 1.63E-03 | -1.06 |
| TMEM220 | 8.77E-03 | -1.06 |
| ABI3BP | 3.83E-02 | -1.07 |
| C3orf14 | 3.21E-04 | -1.07 |
| CAPNS1 | 1.46E-08 | -1.07 |
| KRT15 | 3.34E-02 | -1.07 |
| MGST2 | 1.78E-06 | -1.07 |
| PLEKHG6 | 1.11E-02 | -1.07 |
| RHPN2 | 2.84E-03 | -1.07 |
| ACOT11 | 1.95E-03 | -1.08 |
| BEX5 | 2.81E-02 | -1.08 |
| CYP4X1 | 8.05E-04 | -1.08 |
| EVPL | 1.30E-02 | -1.08 |
| MYEF2 | 8.95E-03 | -1.08 |
| WNT5B | 1.88E-04 | -1.08 |
| ALCAM | 2.30E-04 | -1.09 |
| AR | 1.41E-02 | -1.09 |
| ARHGAP5 | 6.25E-04 | -1.09 |
| GNA14 | 1.26E-03 | -1.09 |
| KCNAB1 | 7.40E-04 | -1.09 |
| MYRIP | 3.23E-03 | -1.09 |
| OR7E47P | 1.25E-02 | -1.09 |
| PDLIM2 | 4.30E-04 | -1.09 |
| SNCA | 4.01E-03 | -1.09 |
| BEX2 | 6.49E-04 | -1.1 |
| CREB3L4 | 1.44E-02 | -1.1 |
| DTX2 | 7.53E-05 | -1.1 |
| FOXC1 | 1.02E-03 | -1.1 |
| HES1 | 3.47E-04 | -1.1 |
| MPC1 | 2.05E-08 | -1.1 |
| SH3YL1 | 6.32E-06 | -1.1 |
| SMAD9 | 1.21E-02 | -1.1 |
| SYTL5 | 4.65E-02 | -1.1 |
| ATP6V0E2 | 3.13E-04 | -1.11 |
| DCXR | 1.55E-07 | -1.11 |
| FAM189A2 | 1.37E-02 | -1.11 |
| KIAA0232 | 1.95E-04 | -1.11 |
| MANSC1 | 1.53E-06 | -1.11 |
| NR2F1 | 2.05E-03 | -1.11 |
| PRKAB1 | 5.25E-07 | -1.11 |
| SCN7A | 4.02E-02 | -1.11 |
| SH3PXD2A-AS1 | 3.26E-02 | -1.11 |
| SPTLC1 | 5.30E-03 | -1.11 |
| C3orf67 | 8.58E-04 | -1.12 |
| CD46 | 2.50E-06 | -1.12 |
| ERO1A | 2.20E-02 | -1.12 |
| FAR1 | 4.85E-05 | -1.12 |
| FCER1A | 2.67E-03 | -1.12 |
| MAOA | 2.67E-03 | -1.12 |
| PPID | 4.41E-03 | -1.12 |
| CPEB3 | 1.18E-05 | -1.13 |
| PPARGC1A | 1.73E-02 | -1.13 |
| S100A14 | 7.15E-05 | -1.13 |
| WNK4 | 3.88E-02 | -1.13 |
| ZDHHC20 | 4.87E-05 | -1.13 |
| AFDN | 1.67E-07 | -1.14 |
| FBXO34 | 1.16E-09 | -1.14 |
| GADD45B | 7.31E-04 | -1.14 |
| PHC1 | 4.10E-05 | -1.14 |
| TMEM8A | 1.27E-06 | -1.14 |
| WFDC21P | 2.13E-02 | -1.14 |
| ADGRG6 | 8.18E-05 | -1.15 |
| B3GNT3 | 1.16E-02 | -1.15 |
| FA2H | 3.62E-03 | -1.15 |
| FUT2 | 4.07E-03 | -1.15 |
| MIPEPP3 | 3.17E-02 | -1.15 |
| NHLH2 | 8.51E-03 | -1.15 |
| ALDH6A1 | 1.30E-05 | -1.16 |
| ARHGAP27 | 1.54E-06 | -1.16 |
| CCND1 | 9.90E-05 | -1.16 |
| NFIA | 1.98E-03 | -1.16 |
| PRKACB | 1.33E-03 | -1.16 |
| SH2D1B | 2.80E-02 | -1.16 |
| STRADB | 7.75E-07 | -1.16 |
| NECTIN2 | 5.07E-06 | -1.17 |
| RALA | 6.14E-07 | -1.17 |
| ABCD3 | 3.02E-03 | -1.18 |
| CRIP1 | 4.30E-04 | -1.18 |
| MGLL | 1.18E-04 | -1.18 |
| SPAG1 | 4.97E-03 | -1.18 |
| ZSCAN18 | 9.21E-04 | -1.18 |
| ENPP5 | 3.64E-02 | -1.19 |
| LRRK2 | 1.34E-06 | -1.19 |
| MALL | 5.51E-04 | -1.19 |
| SLC1A1 | 3.50E-03 | -1.19 |
| ST3GAL1 | 5.63E-06 | -1.19 |
| AHNAK | 9.97E-03 | -1.2 |
| CCNYL1 | 6.36E-04 | -1.2 |
| DBNDD1 | 1.69E-03 | -1.2 |
| ITCH | 1.09E-04 | -1.2 |
| MAB21L3 | 3.89E-02 | -1.2 |
| MYEOV | 1.01E-03 | -1.2 |
| P2RY2 | 1.31E-02 | -1.2 |
| PMM1 | 5.98E-05 | -1.2 |
| TPCN1 | 3.60E-07 | -1.2 |
| ATP8A1 | 1.01E-02 | -1.21 |
| NME7 | 6.90E-05 | -1.21 |
| PAIP2B | 1.64E-03 | -1.21 |
| SUSD4 | 1.72E-03 | -1.21 |
| TMCO4 | 1.51E-05 | -1.21 |
| TSPAN13 | 2.30E-06 | -1.21 |
| C7 | 4.38E-02 | -1.22 |
| NTN1 | 1.12E-03 | -1.22 |
| TPRG1 | 3.86E-03 | -1.22 |
| ZNF823 | 9.31E-06 | -1.22 |
| C15orf59 | 3.60E-03 | -1.23 |
| CLYBL | 1.61E-07 | -1.23 |
| MYO5C | 1.12E-03 | -1.23 |
| PGAP3 | 6.98E-07 | -1.23 |
| TICAM1 | 2.27E-05 | -1.23 |
| TLR3 | 2.42E-04 | -1.23 |
| ZNF519 | 2.89E-04 | -1.23 |
| C1QTNF7 | 2.76E-02 | -1.24 |
| KLB | 2.41E-04 | -1.24 |
| SCNN1A | 3.17E-05 | -1.24 |
| GALE | 1.56E-06 | -1.25 |
| IL23A | 1.66E-02 | -1.25 |
| MEIS1 | 2.48E-05 | -1.25 |
| PTK6 | 2.74E-03 | -1.25 |
| RASAL2 | 7.05E-04 | -1.25 |
| RBPMS | 9.23E-05 | -1.25 |
| RHOF | 5.36E-04 | -1.25 |
| UBL3 | 1.40E-08 | -1.25 |
| VAV3 | 3.05E-02 | -1.25 |
| DIO2 | 4.58E-02 | -1.26 |
| PHACTR4 | 1.94E-04 | -1.26 |
| ZBED2 | 2.76E-02 | -1.26 |
| CDC42EP5 | 2.43E-04 | -1.27 |
| CTTNBP2 | 2.26E-03 | -1.27 |
| DOCK9 | 1.29E-03 | -1.27 |
| DUSP16 | 2.17E-04 | -1.27 |
| FRAS1 | 2.70E-02 | -1.27 |
| GPR160 | 3.74E-02 | -1.27 |
| NSUN7 | 1.33E-04 | -1.27 |
| PWWP2B | 2.45E-07 | -1.27 |
| TNFRSF11A | 7.84E-05 | -1.27 |
| CD24 | 6.92E-04 | -1.28 |
| CLIC3 | 1.30E-02 | -1.28 |
| EHF | 3.83E-05 | -1.28 |
| FAM107A | 5.76E-03 | -1.28 |
| FAM149A | 1.07E-03 | -1.28 |
| GRIA2 | 4.65E-02 | -1.28 |
| MAGI3 | 5.67E-08 | -1.28 |
| MBOAT1 | 7.15E-07 | -1.28 |
| PDCD4 | 1.19E-08 | -1.28 |
| TMEM71 | 7.16E-03 | -1.28 |
| ZNF92 | 6.02E-06 | -1.28 |
| CDKN2AIP | 9.52E-09 | -1.29 |
| CGN | 1.25E-03 | -1.29 |
| CLDN11 | 1.47E-02 | -1.29 |
| DUOX2 | 1.37E-02 | -1.29 |
| RALGPS1 | 4.39E-06 | -1.29 |
| RBM47 | 3.57E-10 | -1.29 |
| RNASE4 | 1.91E-02 | -1.29 |
| CDKN2B | 2.62E-02 | -1.3 |
| CLDN4 | 9.90E-07 | -1.3 |
| MYCN | 9.01E-06 | -1.3 |
| NEDD4L | 8.26E-05 | -1.3 |
| TMOD3 | 4.06E-05 | -1.3 |
| ANXA1 | 2.78E-02 | -1.31 |
| HIST1H3F | 6.20E-04 | -1.31 |
| ID4 | 1.47E-02 | -1.31 |
| SOX2 | 2.09E-03 | -1.31 |
| ANXA11 | 8.49E-11 | -1.32 |
| BLNK | 7.38E-07 | -1.32 |
| C6orf132 | 1.76E-03 | -1.32 |
| TMEM120A | 8.70E-09 | -1.32 |
| PPL | 2.01E-05 | -1.33 |
| ABLIM3 | 2.69E-04 | -1.34 |
| ACP6 | 8.36E-07 | -1.34 |
| CA13 | 2.32E-06 | -1.34 |
| ETHE1 | 8.76E-07 | -1.34 |
| FAM129B | 1.27E-05 | -1.34 |
| KLF3-AS1 | 3.45E-04 | -1.34 |
| ACPP | 8.02E-03 | -1.35 |
| ADRB1 | 1.15E-03 | -1.35 |
| SLC25A23 | 8.96E-07 | -1.35 |
| ABHD11 | 1.48E-08 | -1.36 |
| ARHGAP20 | 1.17E-03 | -1.36 |
| ENPP4 | 3.18E-03 | -1.36 |
| HIST1H2BJ | 1.10E-02 | -1.36 |
| RBP7 | 2.23E-03 | -1.36 |
| SLC13A4 | 2.86E-04 | -1.36 |
| STK39 | 3.30E-09 | -1.36 |
| ACOX1 | 2.12E-08 | -1.37 |
| STRBP | 9.07E-08 | -1.37 |
| ICA1 | 4.46E-06 | -1.38 |
| LCN2 | 2.22E-04 | -1.38 |
| MSLN | 4.16E-03 | -1.38 |
| SLC27A2 | 4.99E-04 | -1.38 |
| C1GALT1 | 3.46E-06 | -1.39 |
| CYSLTR1 | 9.90E-06 | -1.39 |
| IL18 | 1.90E-03 | -1.39 |
| MDFIC | 1.08E-04 | -1.39 |
| PARD6B | 1.58E-05 | -1.39 |
| TMPRSS4 | 1.03E-04 | -1.39 |
| A2ML1 | 1.08E-02 | -1.4 |
| AFDN-AS1 | 5.63E-05 | -1.4 |
| AIM1L | 2.81E-03 | -1.4 |
| GFRA1 | 3.91E-04 | -1.4 |
| NT5C2 | 9.42E-13 | -1.4 |
| VGLL1 | 1.78E-03 | -1.4 |
| CRTAC1 | 7.44E-04 | -1.41 |
| GDPD3 | 9.59E-04 | -1.41 |
| GLYATL2 | 2.82E-02 | -1.41 |
| MECOM | 2.22E-04 | -1.41 |
| NOVA1 | 6.70E-04 | -1.41 |
| RORC | 1.17E-03 | -1.41 |
| RRAGD | 2.43E-05 | -1.41 |
| CRIP2 | 2.80E-04 | -1.42 |
| DEPTOR | 4.39E-03 | -1.42 |
| GCNT2 | 1.15E-05 | -1.42 |
| OCLN | 1.23E-04 | -1.42 |
| FAM214A | 9.33E-04 | -1.43 |
| OBFC1 | 3.32E-07 | -1.43 |
| FMO1 | 2.33E-02 | -1.44 |
| PTGDS | 3.52E-02 | -1.44 |
| SHMT1 | 2.00E-05 | -1.44 |
| STXBP6 | 2.62E-04 | -1.44 |
| DHRS11 | 1.02E-04 | -1.45 |
| FETUB | 2.38E-02 | -1.45 |
| FNDC4 | 1.31E-04 | -1.45 |
| OGFRL1 | 2.30E-11 | -1.45 |
| PDE8B | 5.76E-03 | -1.45 |
| SIDT1 | 2.49E-03 | -1.45 |
| UPK3B | 3.42E-05 | -1.45 |
| DOPEY2 | 1.03E-04 | -1.46 |
| HLF | 8.22E-05 | -1.46 |
| PPP1R9A | 2.21E-02 | -1.46 |
| SYT8 | 2.44E-05 | -1.46 |
| TAB3 | 2.19E-04 | -1.46 |
| ZNF844 | 1.35E-05 | -1.46 |
| SLC27A6 | 3.82E-03 | -1.47 |
| FBXO32 | 1.10E-04 | -1.48 |
| SLC6A4 | 4.11E-03 | -1.48 |
| TRIP10 | 7.69E-07 | -1.48 |
| HPGD | 2.33E-03 | -1.49 |
| MMRN1 | 9.36E-04 | -1.5 |
| PPCDC | 3.46E-08 | -1.5 |
| SPAG16 | 2.65E-08 | -1.5 |
| SYNPO2 | 3.85E-03 | -1.5 |
| ATP1B1 | 9.85E-15 | -1.51 |
| EPS8L2 | 6.27E-04 | -1.51 |
| TIMP2 | 2.51E-03 | -1.51 |
| MYO5B | 1.61E-06 | -1.52 |
| PADI3 | 1.59E-03 | -1.52 |
| RMND5B | 3.89E-09 | -1.52 |
| SPINT1 | 6.71E-06 | -1.52 |
| VPS37B | 3.56E-08 | -1.52 |
| MXD1 | 1.38E-02 | -1.53 |
| BCKDHB | 4.11E-06 | -1.54 |
| ITGB8 | 3.80E-05 | -1.54 |
| PLLP | 1.67E-04 | -1.54 |
| C1orf116 | 3.79E-05 | -1.55 |
| ST3GAL4 | 4.07E-07 | -1.55 |
| SPNS2 | 8.25E-05 | -1.56 |
| COX10 | 4.23E-05 | -1.57 |
| ADGRG2 | 1.28E-03 | -1.58 |
| ELOVL6 | 3.31E-04 | -1.58 |
| MLPH | 1.08E-02 | -1.58 |
| RAB11FIP1 | 1.50E-09 | -1.58 |
| SRPX2 | 8.21E-05 | -1.58 |
| TP53INP2 | 1.21E-05 | -1.58 |
| ZNF101 | 1.09E-06 | -1.58 |
| GNG4 | 1.56E-03 | -1.59 |
| VSIG10 | 1.88E-04 | -1.59 |
| VSIG2 | 6.68E-06 | -1.59 |
| ALDH3A1 | 5.72E-03 | -1.6 |
| GGT6 | 3.29E-05 | -1.6 |
| NCEH1 | 7.79E-06 | -1.6 |
| ALDH1L1 | 1.04E-05 | -1.61 |
| ATP13A4 | 1.83E-04 | -1.61 |
| CHPT1 | 2.07E-05 | -1.61 |
| NR3C2 | 3.81E-05 | -1.61 |
| SELENBP1 | 5.37E-04 | -1.61 |
| EDAR | 9.99E-06 | -1.62 |
| EPHX2 | 1.78E-05 | -1.62 |
| KIAA1211L | 4.01E-05 | -1.62 |
| PLCB4 | 1.45E-02 | -1.62 |
| FGFBP2 | 1.78E-02 | -1.63 |
| GULP1 | 1.73E-05 | -1.63 |
| HBB | 3.74E-02 | -1.63 |
| MYOC | 1.79E-02 | -1.63 |
| TCEAL2 | 2.24E-03 | -1.63 |
| TRIM6 | 1.36E-06 | -1.63 |
| WWC1 | 5.37E-04 | -1.63 |
| IL1A | 1.91E-02 | -1.64 |
| KLHDC8A | 2.72E-03 | -1.64 |
| KRT13 | 1.00E-04 | -1.64 |
| LEPROT | 3.16E-05 | -1.65 |
| SNORA68 | 1.02E-03 | -1.65 |
| SUN1 | 2.84E-05 | -1.65 |
| TC2N | 2.86E-14 | -1.65 |
| F3 | 3.02E-06 | -1.66 |
| SLC15A2 | 1.01E-03 | -1.66 |
| SMIM5 | 2.38E-04 | -1.66 |
| PCBP1 | 3.14E-08 | -1.67 |
| LGALS3 | 3.55E-04 | -1.68 |
| CPPED1 | 2.22E-10 | -1.69 |
| COCH | 8.29E-04 | -1.7 |
| SORT1 | 2.66E-07 | -1.7 |
| COLCA2 | 3.63E-07 | -1.71 |
| FAM84B | 1.24E-10 | -1.72 |
| LRG1 | 3.18E-03 | -1.72 |
| HS6ST2 | 1.87E-04 | -1.73 |
| LYVE1 | 5.34E-03 | -1.73 |
| USP6NL | 1.55E-05 | -1.74 |
| CSTB | 5.79E-04 | -1.76 |
| ARFGEF3 | 3.19E-02 | -1.77 |
| GPT2 | 1.11E-06 | -1.77 |
| SPINK5 | 3.98E-03 | -1.77 |
| CALB2 | 5.09E-04 | -1.78 |
| CHST9 | 1.97E-02 | -1.78 |
| LIPH | 9.71E-07 | -1.78 |
| AFF3 | 3.59E-03 | -1.79 |
| CYP2J2 | 6.46E-04 | -1.79 |
| TMC4 | 7.94E-05 | -1.79 |
| TSPAN1 | 3.36E-02 | -1.79 |
| B4GALT5 | 3.26E-09 | -1.8 |
| C2orf40 | 4.27E-03 | -1.8 |
| SLC37A1 | 2.99E-11 | -1.8 |
| SMAGP | 1.42E-06 | -1.8 |
| ADH7 | 3.39E-04 | -1.81 |
| DHRS9 | 7.75E-04 | -1.81 |
| PRKAA2 | 1.96E-02 | -1.82 |
| CLDN23 | 3.27E-05 | -1.83 |
| CLU | 1.31E-03 | -1.83 |
| GBP6 | 5.11E-05 | -1.83 |
| REEP6 | 7.33E-06 | -1.83 |
| CCNG2 | 6.73E-06 | -1.85 |
| PTN | 2.14E-05 | -1.85 |
| BARX2 | 2.88E-05 | -1.87 |
| ENDOU | 3.50E-03 | -1.88 |
| HMGCS2 | 1.09E-02 | -1.88 |
| PITX1 | 1.38E-04 | -1.88 |
| SERPINB2 | 7.85E-05 | -1.88 |
| SLITRK5 | 6.83E-05 | -1.88 |
| ANXA3 | 6.95E-05 | -1.89 |
| CYSRT1 | 9.70E-04 | -1.89 |
| LDOC1 | 1.46E-08 | -1.89 |
| LIFR | 3.23E-05 | -1.89 |
| MAMDC2 | 6.92E-04 | -1.9 |
| SOX9 | 3.60E-05 | -1.9 |
| LNX1 | 9.94E-11 | -1.91 |
| PPP1R1B | 1.05E-02 | -1.91 |
| BCAS1 | 5.59E-10 | -1.92 |
| NUCB2 | 3.48E-07 | -1.92 |
| CAB39L | 8.93E-06 | -1.93 |
| SSTR5 | 3.46E-02 | -1.93 |
| PHLDA1 | 2.90E-07 | -1.94 |
| MEIS2 | 7.58E-10 | -1.95 |
| CLIC6 | 1.55E-04 | -1.96 |
| DUSP5 | 1.20E-05 | -1.96 |
| KRT18 | 1.22E-07 | -1.96 |
| NTRK3 | 5.32E-07 | -1.96 |
| SLC44A3 | 1.12E-08 | -1.96 |
| CRISP2 | 2.87E-03 | -1.97 |
| CDH26 | 3.09E-05 | -1.98 |
| CEL | 1.56E-03 | -1.98 |
| ETV1 | 7.86E-03 | -1.98 |
| LEXM | 2.28E-05 | -1.98 |
| MAOB | 7.94E-05 | -1.99 |
| RASEF | 1.98E-07 | -1.99 |
| SERPINB1 | 1.28E-08 | -1.99 |
| C18orf25 | 7.42E-05 | -2 |
| C9orf152 | 7.87E-05 | -2 |
| ADH1B | 1.30E-02 | -2.01 |
| ALDH1A3 | 6.47E-08 | -2.02 |
| RNF128 | 2.75E-05 | -2.02 |
| SAMD5 | 2.27E-05 | -2.03 |
| MACC1 | 1.19E-07 | -2.04 |
| RFK | 6.66E-11 | -2.04 |
| C15orf48 | 1.83E-06 | -2.05 |
| ESPL1 | 1.38E-06 | -2.05 |
| GREM2 | 4.59E-03 | -2.06 |
| NDRG2 | 1.33E-07 | -2.06 |
| FAM221A | 1.73E-13 | -2.07 |
| LPIN1 | 1.88E-07 | -2.07 |
| SLC2A12 | 2.06E-05 | -2.07 |
| SLC35C1 | 6.09E-07 | -2.07 |
| EPS8L1 | 2.60E-05 | -2.08 |
| CP | 5.90E-05 | -2.09 |
| FOXA1 | 1.76E-06 | -2.09 |
| IKZF2 | 1.22E-10 | -2.1 |
| SERPINB11 | 3.18E-02 | -2.1 |
| WFDC2 | 3.58E-02 | -2.1 |
| SFTA2 | 2.09E-03 | -2.12 |
| TSPAN6 | 8.29E-12 | -2.13 |
| GMDS | 5.57E-11 | -2.15 |
| PRR15L | 4.11E-06 | -2.15 |
| AIF1L | 6.19E-06 | -2.16 |
| PP14571 | 1.32E-06 | -2.17 |
| VIT | 1.97E-05 | -2.17 |
| ACTG1P4 | 5.04E-04 | -2.18 |
| GSTA1 | 1.06E-02 | -2.18 |
| SLC4A4 | 2.90E-05 | -2.18 |
| AIFM2 | 2.88E-05 | -2.2 |
| CLDN8 | 5.93E-05 | -2.21 |
| TMPRSS11E | 3.20E-04 | -2.21 |
| TCP11L2 | 3.33E-05 | -2.22 |
| CEACAM6 | 1.17E-07 | -2.23 |
| IL1R2 | 3.96E-05 | -2.23 |
| GPRC5A | 6.81E-04 | -2.24 |
| MYZAP | 1.76E-07 | -2.24 |
| PLEKHA7 | 8.20E-09 | -2.24 |
| ST6GALNAC1 | 4.80E-07 | -2.26 |
| TFF1 | 3.37E-02 | -2.28 |
| C5orf66 | 8.89E-07 | -2.29 |
| COBL | 2.92E-05 | -2.29 |
| BEX4 | 4.72E-09 | -2.3 |
| C4orf19 | 1.12E-05 | -2.3 |
| HS3ST1 | 2.35E-09 | -2.3 |
| MFSD4A | 2.19E-04 | -2.31 |
| PROM1 | 4.32E-02 | -2.31 |
| CAPN5 | 1.70E-08 | -2.32 |
| PLS1 | 8.62E-08 | -2.32 |
| PAQR8 | 1.48E-08 | -2.33 |
| KRT7 | 1.15E-04 | -2.34 |
| IGHD | 2.71E-02 | -2.35 |
| KIAA1324 | 1.42E-02 | -2.35 |
| LYZ | 3.36E-02 | -2.36 |
| RHCG | 2.11E-07 | -2.36 |
| SCEL | 1.30E-05 | -2.36 |
| CEACAM5 | 6.23E-08 | -2.4 |
| IL1RN | 1.29E-06 | -2.4 |
| C2orf54 | 8.23E-10 | -2.41 |
| GPD1L | 2.83E-10 | -2.41 |
| TFF3 | 2.07E-02 | -2.42 |
| TOX3 | 3.20E-03 | -2.42 |
| CLGN | 1.40E-05 | -2.43 |
| PEG3 | 4.04E-04 | -2.43 |
| SVIP | 5.73E-13 | -2.43 |
| GNE | 2.13E-11 | -2.44 |
| B3GALT5 | 5.31E-06 | -2.45 |
| S100P | 1.22E-03 | -2.46 |
| HCG22 | 1.53E-04 | -2.47 |
| ELF3 | 5.07E-09 | -2.48 |
| EYA2 | 2.41E-10 | -2.48 |
| SCGB2A1 | 2.04E-02 | -2.48 |
| CYP2E1 | 2.92E-04 | -2.51 |
| ECM1 | 1.85E-06 | -2.51 |
| ANKRD20A12P | 1.05E-10 | -2.54 |
| ABAT | 2.33E-13 | -2.55 |
| PKIB | 5.56E-10 | -2.55 |
| ANXA9 | 3.28E-04 | -2.57 |
| CYP4B1 | 3.46E-05 | -2.57 |
| LMO7 | 6.45E-08 | -2.58 |
| CLDN7 | 1.14E-12 | -2.59 |
| CYP3A5 | 3.25E-07 | -2.59 |
| GALNT5 | 1.31E-09 | -2.6 |
| PPP1R3C | 7.57E-07 | -2.6 |
| RBM20 | 3.17E-05 | -2.6 |
| TTC9 | 8.87E-12 | -2.6 |
| SLC26A2 | 6.19E-10 | -2.61 |
| BTC | 1.84E-09 | -2.63 |
| CXCL17 | 2.17E-10 | -2.64 |
| EHD3 | 5.78E-09 | -2.64 |
| C6orf58 | 2.93E-02 | -2.67 |
| NKX3-1 | 1.09E-02 | -2.67 |
| UGT1A1 | 3.69E-05 | -2.67 |
| LRMP | 2.99E-04 | -2.68 |
| CAMK2N1 | 1.13E-11 | -2.69 |
| FLG-AS1 | 7.72E-08 | -2.75 |
| ALDH1A1 | 2.09E-05 | -2.76 |
| PAX9 | 1.04E-11 | -2.77 |
| PRSS27 | 9.92E-06 | -2.8 |
| CGNL1 | 1.74E-10 | -2.81 |
| TJP3 | 1.84E-09 | -2.84 |
| OR7E14P | 4.70E-08 | -2.87 |
| EMP1 | 5.07E-09 | -2.89 |
| C15orf62 | 2.89E-08 | -2.9 |
| HLCS | 2.10E-07 | -2.91 |
| AGR3 | 2.85E-03 | -2.92 |
| TRNP1 | 3.72E-07 | -2.93 |
| SHROOM3 | 3.04E-11 | -2.95 |
| SYTL4 | 9.78E-10 | -2.95 |
| SORBS2 | 2.11E-11 | -2.96 |
| IL36A | 4.96E-05 | -2.97 |
| DMBT1 | 1.52E-02 | -3.01 |
| UGT1A3 | 3.79E-05 | -3.01 |
| FUT3 | 3.48E-11 | -3.02 |
| MAL | 8.46E-07 | -3.14 |
| SCGB1A1 | 3.34E-04 | -3.15 |
| GCHFR | 1.18E-11 | -3.16 |
| SH3BGRL2 | 8.69E-12 | -3.17 |
| CRNN | 1.49E-04 | -3.19 |
| UPK1B | 8.81E-05 | -3.31 |
| GALNT12 | 1.98E-15 | -3.33 |
| MUC7 | 1.11E-02 | -3.33 |
| FUT6 | 3.36E-12 | -3.39 |
| AGFG2 | 9.17E-11 | -3.4 |
| KRT78 | 4.67E-07 | -3.45 |
| BPIFB2 | 4.66E-03 | -3.47 |
| SCGB3A1 | 8.45E-03 | -3.48 |
| LTF | 7.22E-03 | -3.5 |
| TF | 4.44E-06 | -3.51 |
| PRH1 | 1.28E-02 | -3.54 |
| CLCA4 | 3.33E-11 | -3.59 |
| PIP | 8.66E-03 | -3.6 |
| PAX1 | 4.45E-05 | -3.62 |
| ETNK2 | 1.89E-08 | -3.65 |
| PIGR | 1.83E-02 | -3.67 |
| TMPRSS11B | 1.20E-07 | -3.71 |
| CRYM | 9.95E-13 | -3.75 |
| FAM3B | 1.80E-13 | -3.76 |
| FDCSP | 6.48E-03 | -3.77 |
| MUC20 | 4.08E-14 | -3.8 |
| SNX31 | 1.27E-09 | -3.81 |
| ADGRF1 | 1.57E-11 | -3.84 |
| SLC8A1-AS1 | 4.06E-08 | -3.85 |
| MUC1 | 1.01E-10 | -3.92 |
| FMO9P | 5.32E-07 | -3.93 |
| TMPRSS2 | 2.31E-15 | -3.93 |
| PSCA | 4.94E-11 | -3.95 |
| ATP6V0A4 | 2.54E-13 | -4.05 |
| ANKRD20A11P | 3.48E-15 | -4.19 |
| CEACAM1 | 2.86E-14 | -4.24 |
| GABRP | 5.59E-07 | -4.31 |
| ANKRD20A5P | 8.36E-12 | -4.32 |
| PADI1 | 9.77E-12 | -4.33 |
| SIM2 | 2.52E-13 | -4.33 |
| FAM3D | 4.12E-14 | -4.37 |
| STATH | 3.52E-03 | -4.43 |
| BPIFB1 | 1.43E-02 | -4.46 |
| PLAC8 | 5.17E-09 | -4.5 |
| MUC4 | 3.09E-08 | -4.52 |
| ANKRD20A4 | 4.45E-14 | -4.55 |
| SCIN | 2.49E-14 | -4.55 |
| GCNT3 | 4.66E-11 | -4.63 |
| MUC5B | 2.96E-03 | -4.69 |
| KRT4 | 2.73E-08 | -5.11 |
| KRT19 | 1.15E-13 | -5.23 |
| CAPN14 | 2.17E-15 | -5.24 |
| ZG16B | 1.03E-04 | -5.26 |
| AGR2 | 1.30E-08 | -5.69 |
| CLDN10 | 7.55E-08 | -5.72 |
| CRISP3 | 1.07E-12 | -6.42 |

DEG, differentially expressed genes; FDR, false discovery rate; FC, fold change.

**Supplementary Table 2.** A total of 947 DEGs were identified between primary oral squamous cell carcinoma and oral dysplasia.

| **Gene symbol** | **FDR** | **logFC** |
| --- | --- | --- |
| MMP3 | 6.44E-08 | 5.19 |
| MMP1 | 1.69E-08 | 5.05 |
| CXCL8 | 3.96E-09 | 4.42 |
| SPP1 | 1.40E-06 | 4.24 |
| MMP10 | 1.30E-04 | 4.05 |
| MMP13 | 3.20E-04 | 3.85 |
| INHBA | 1.23E-05 | 3.51 |
| MMP12 | 1.12E-07 | 3.5 |
| CXCL13 | 7.40E-06 | 3.47 |
| DSG2 | 8.10E-10 | 3.39 |
| SERPINE1 | 9.39E-06 | 3.33 |
| LAMC2 | 3.98E-06 | 3.07 |
| CXCL5 | 7.69E-04 | 3.02 |
| CXCL1 | 7.97E-07 | 2.99 |
| CXCL6 | 1.36E-04 | 2.8 |
| HMGA2 | 1.07E-03 | 2.68 |
| CCL3L3 | 1.40E-06 | 2.67 |
| PTHLH | 4.44E-03 | 2.66 |
| CXCL2 | 1.04E-06 | 2.6 |
| MMP7 | 1.15E-03 | 2.59 |
| CCL20 | 1.57E-03 | 2.56 |
| COL10A1 | 1.69E-03 | 2.55 |
| PLAU | 5.99E-06 | 2.45 |
| SERPINA1 | 3.11E-07 | 2.42 |
| ITGB6 | 1.34E-04 | 2.4 |
| CXCL11 | 2.01E-02 | 2.39 |
| IGHM | 8.68E-03 | 2.39 |
| IGK | 7.03E-03 | 2.39 |
| IL24 | 2.48E-03 | 2.39 |
| DKK1 | 2.80E-03 | 2.38 |
| COL11A1 | 4.62E-02 | 2.37 |
| IGF2BP3 | 3.12E-03 | 2.37 |
| BCL2A1 | 2.89E-06 | 2.35 |
| AGR2 | 1.93E-02 | 2.33 |
| TNFRSF12A | 4.61E-06 | 2.33 |
| PLA2G7 | 9.42E-08 | 2.32 |
| RGS1 | 8.93E-06 | 2.32 |
| APOC1 | 3.82E-05 | 2.31 |
| CCL4 | 2.43E-06 | 2.3 |
| UBD | 2.06E-03 | 2.28 |
| ICAM1 | 1.42E-06 | 2.27 |
| IGLC1 | 1.61E-02 | 2.25 |
| IL6 | 2.86E-03 | 2.21 |
| PDPN | 8.29E-05 | 2.21 |
| MMP9 | 1.71E-05 | 2.19 |
| IDO1 | 4.49E-03 | 2.17 |
| PLAUR | 1.49E-07 | 2.16 |
| FPR1 | 4.91E-06 | 2.15 |
| IGKC | 2.47E-02 | 2.13 |
| IGHD | 4.89E-02 | 2.12 |
| RGS4 | 1.52E-03 | 2.11 |
| MMP11 | 4.61E-03 | 2.07 |
| BMS1P20 | 2.54E-02 | 2.06 |
| CXCL3 | 1.20E-03 | 2.06 |
| WDR66 | 1.67E-03 | 2.05 |
| TDO2 | 1.30E-02 | 2.03 |
| TSPAN1 | 2.60E-03 | 2.02 |
| CHST11 | 2.48E-07 | 2.01 |
| CXCR4 | 5.98E-05 | 2 |
| IGKV1OR2 | 1.23E-02 | 1.98 |
| APOBEC3A | 2.48E-03 | 1.97 |
| FCGR1CP | 5.18E-05 | 1.97 |
| LYZ | 2.19E-02 | 1.96 |
| APOE | 2.56E-04 | 1.95 |
| RSAD2 | 1.28E-02 | 1.95 |
| TNFAIP3 | 3.04E-09 | 1.94 |
| ADAMDEC1 | 3.76E-03 | 1.93 |
| MLIP | 1.42E-02 | 1.93 |
| SERPINA3 | 4.82E-02 | 1.93 |
| GREM1 | 5.18E-04 | 1.92 |
| FST | 2.65E-03 | 1.91 |
| IL1B | 1.35E-02 | 1.91 |
| THBS1 | 9.75E-05 | 1.91 |
| CXCL10 | 3.50E-02 | 1.89 |
| CYR61 | 1.63E-04 | 1.89 |
| IGLJ3 | 2.23E-02 | 1.89 |
| SOX9 | 3.64E-05 | 1.88 |
| C5AR1 | 6.32E-06 | 1.85 |
| CST1 | 3.20E-02 | 1.84 |
| IGHV3 | 2.60E-02 | 1.83 |
| SLCO1B3 | 1.37E-02 | 1.83 |
| TLR2 | 1.12E-07 | 1.82 |
| CD86 | 1.83E-06 | 1.81 |
| DUXAP10 | 9.52E-03 | 1.81 |
| CXCL9 | 4.48E-02 | 1.8 |
| RAB20 | 1.96E-08 | 1.76 |
| SLFN5 | 4.20E-05 | 1.76 |
| SOCS3 | 2.16E-06 | 1.76 |
| SULF1 | 4.33E-03 | 1.76 |
| RARRES1 | 1.45E-02 | 1.7 |
| FCGR1B | 9.60E-04 | 1.69 |
| KRT18 | 3.94E-04 | 1.69 |
| RHPN2 | 9.92E-06 | 1.69 |
| THEMIS2 | 5.71E-07 | 1.68 |
| IL7R | 9.99E-05 | 1.67 |
| AIM2 | 5.96E-03 | 1.66 |
| F3 | 5.14E-04 | 1.66 |
| FAP | 2.08E-02 | 1.66 |
| PRR5 | 4.99E-04 | 1.66 |
| SCG5 | 1.37E-02 | 1.66 |
| TREM1 | 7.10E-03 | 1.66 |
| MARCKSL1 | 2.72E-08 | 1.65 |
| MDK | 2.30E-04 | 1.64 |
| GBP5 | 7.94E-03 | 1.63 |
| IGHV4 | 4.48E-02 | 1.63 |
| ISG15 | 5.81E-03 | 1.63 |
| G0S2 | 1.10E-02 | 1.62 |
| IGLV1 | 6.93E-03 | 1.62 |
| LAMB3 | 4.57E-04 | 1.62 |
| CD300A | 1.31E-05 | 1.61 |
| IQCG | 2.60E-02 | 1.61 |
| CAMK2N1 | 7.79E-04 | 1.6 |
| KLF7 | 5.98E-05 | 1.6 |
| FKBP5 | 6.92E-03 | 1.59 |
| NCEH1 | 2.29E-05 | 1.59 |
| NRIP3 | 7.60E-04 | 1.59 |
| AREG | 2.35E-02 | 1.58 |
| LAMA3 | 7.76E-03 | 1.58 |
| PLEK2 | 1.94E-03 | 1.58 |
| CD163 | 2.22E-03 | 1.57 |
| CDK6 | 1.67E-03 | 1.57 |
| GNLY | 1.88E-03 | 1.57 |
| SRGN | 5.99E-06 | 1.57 |
| TGM2 | 5.31E-05 | 1.57 |
| CYP27B1 | 3.23E-03 | 1.56 |
| P3H2 | 3.26E-02 | 1.56 |
| HAS2 | 1.43E-02 | 1.55 |
| SOD2 | 1.20E-02 | 1.55 |
| CYAT1 | 7.49E-03 | 1.54 |
| FN1 | 2.01E-02 | 1.54 |
| SNX10 | 7.50E-06 | 1.54 |
| C1QB | 1.60E-04 | 1.53 |
| EIF5A2 | 5.54E-04 | 1.53 |
| EPSTI1 | 4.89E-03 | 1.53 |
| MERTK | 8.51E-04 | 1.53 |
| FCGR3B | 7.72E-05 | 1.52 |
| PIK3AP1 | 7.08E-04 | 1.52 |
| SELL | 1.99E-02 | 1.52 |
| ADGRE2 | 4.56E-04 | 1.51 |
| CHI3L1 | 3.65E-02 | 1.51 |
| SOAT1 | 3.98E-07 | 1.51 |
| SHANK2 | 4.82E-02 | 1.5 |
| IFI6 | 2.44E-02 | 1.49 |
| CTSS | 4.80E-05 | 1.48 |
| CCL5 | 1.36E-02 | 1.47 |
| FBXO32 | 1.12E-02 | 1.47 |
| GOLM1 | 9.10E-03 | 1.47 |
| KANK4 | 2.46E-02 | 1.47 |
| CDH3 | 4.99E-04 | 1.46 |
| GZMB | 1.39E-02 | 1.46 |
| KLHL6 | 2.15E-03 | 1.46 |
| PKP2 | 1.88E-03 | 1.46 |
| STC1 | 1.41E-02 | 1.46 |
| TNFAIP6 | 9.39E-03 | 1.46 |
| ITGA3 | 2.10E-04 | 1.45 |
| LARP6 | 2.94E-03 | 1.45 |
| CCL11 | 4.15E-02 | 1.44 |
| ETV1 | 6.10E-03 | 1.44 |
| LAMP5 | 9.94E-03 | 1.44 |
| MFAP2 | 6.82E-03 | 1.44 |
| NEK6 | 3.38E-07 | 1.44 |
| SLC16A3 | 1.17E-03 | 1.44 |
| ADAM12 | 3.85E-02 | 1.43 |
| ADAM19 | 4.00E-04 | 1.43 |
| EVA1A | 1.43E-02 | 1.43 |
| PMEPA1 | 1.54E-03 | 1.43 |
| TENM3 | 2.11E-02 | 1.43 |
| PDE7A | 7.72E-05 | 1.42 |
| XDH | 1.02E-02 | 1.42 |
| AMIGO2 | 2.21E-03 | 1.41 |
| FCGR2A | 9.62E-06 | 1.41 |
| HINT3 | 1.27E-02 | 1.41 |
| PAPPA | 7.20E-04 | 1.41 |
| RAC2 | 6.20E-05 | 1.41 |
| FADS3 | 7.43E-03 | 1.4 |
| FCER1G | 3.35E-05 | 1.4 |
| PI15 | 2.95E-02 | 1.4 |
| SIGLEC10 | 6.74E-04 | 1.4 |
| APOBEC3B | 6.28E-03 | 1.39 |
| ITGB4 | 1.53E-04 | 1.39 |
| SAMSN1 | 1.36E-04 | 1.39 |
| SLC2A3 | 1.51E-04 | 1.39 |
| BCAT1 | 7.82E-03 | 1.38 |
| TFRC | 7.03E-03 | 1.38 |
| MAD2L1 | 1.48E-03 | 1.37 |
| NCF2 | 1.41E-04 | 1.37 |
| SAA2 | 1.46E-02 | 1.37 |
| CTLA4 | 8.10E-03 | 1.36 |
| FOSL1 | 2.57E-02 | 1.36 |
| FSTL3 | 1.16E-02 | 1.36 |
| PDGFC | 1.20E-02 | 1.36 |
| ATP13A3 | 4.87E-05 | 1.35 |
| CCDC71L | 2.30E-04 | 1.35 |
| GCA | 6.42E-06 | 1.35 |
| RTKN | 3.01E-05 | 1.35 |
| STK17B | 2.57E-06 | 1.35 |
| BID | 6.24E-06 | 1.34 |
| RAI14 | 1.97E-04 | 1.34 |
| TNFSF13 | 1.09E-06 | 1.33 |
| IFI30 | 1.51E-05 | 1.32 |
| IL27RA | 3.20E-04 | 1.32 |
| TGFB1 | 1.90E-05 | 1.32 |
| BIRC3 | 4.81E-03 | 1.31 |
| KRT8 | 1.08E-02 | 1.31 |
| NFIL3 | 2.19E-06 | 1.31 |
| RUNX2 | 6.18E-05 | 1.31 |
| SLC7A7 | 6.01E-05 | 1.31 |
| TNFSF4 | 4.48E-03 | 1.31 |
| SOCS1 | 7.53E-05 | 1.3 |
| TGFBI | 6.42E-03 | 1.3 |
| PLPP4 | 3.65E-02 | 1.29 |
| TFEC | 4.06E-03 | 1.29 |
| BCAR3 | 5.60E-05 | 1.28 |
| CYTIP | 2.04E-03 | 1.28 |
| FGD6 | 3.58E-04 | 1.28 |
| IFI35 | 7.21E-04 | 1.28 |
| NEDD4L | 2.41E-04 | 1.28 |
| NOX4 | 1.14E-02 | 1.28 |
| APBB2 | 1.29E-02 | 1.27 |
| SLAMF8 | 1.40E-03 | 1.27 |
| SLC2A14 | 4.10E-04 | 1.27 |
| TREM2 | 5.82E-03 | 1.27 |
| TTYH3 | 2.67E-05 | 1.27 |
| ACTN1 | 2.04E-04 | 1.26 |
| C11orf96 | 8.92E-03 | 1.26 |
| C1QC | 2.40E-04 | 1.26 |
| EFHD2 | 7.88E-07 | 1.26 |
| HAVCR2 | 5.23E-04 | 1.26 |
| LY6E | 4.87E-04 | 1.26 |
| PDE4B | 2.61E-03 | 1.26 |
| MMP14 | 2.17E-02 | 1.25 |
| SEL1L3 | 1.58E-02 | 1.25 |
| SLA | 3.32E-03 | 1.25 |
| ADAM28 | 7.78E-03 | 1.24 |
| CMPK2 | 3.77E-02 | 1.24 |
| FAM26F | 1.49E-02 | 1.24 |
| MICB | 5.25E-04 | 1.24 |
| NRG1 | 3.71E-02 | 1.24 |
| OSMR | 8.08E-04 | 1.24 |
| PLOD2 | 1.68E-03 | 1.24 |
| TNFSF13B | 9.30E-03 | 1.24 |
| BUB1 | 3.17E-04 | 1.23 |
| CDC6 | 4.63E-03 | 1.23 |
| CDKN3 | 6.95E-04 | 1.23 |
| GABPB1 | 6.73E-05 | 1.23 |
| SERPINB9 | 7.16E-04 | 1.23 |
| SLC16A1 | 1.41E-02 | 1.23 |
| CCR1 | 3.46E-03 | 1.22 |
| FBP1 | 2.22E-02 | 1.22 |
| FNDC3B | 2.03E-05 | 1.21 |
| SDC4 | 1.27E-06 | 1.21 |
| ATAD2 | 2.89E-03 | 1.2 |
| DFNA5 | 2.60E-02 | 1.2 |
| LAPTM5 | 1.53E-04 | 1.2 |
| MB21D1 | 3.72E-03 | 1.2 |
| MTHFD1L | 2.24E-04 | 1.2 |
| HDAC9 | 3.21E-03 | 1.19 |
| MS4A7 | 1.38E-03 | 1.19 |
| SLC52A2 | 3.44E-04 | 1.19 |
| TNFSF12 | 1.90E-06 | 1.19 |
| ALCAM | 1.48E-03 | 1.18 |
| BAG2 | 1.51E-02 | 1.18 |
| CTGF | 1.57E-02 | 1.18 |
| MET | 6.17E-03 | 1.18 |
| MIAT | 1.59E-02 | 1.18 |
| WARS | 1.57E-02 | 1.18 |
| ABL2 | 9.13E-05 | 1.17 |
| ANGPT2 | 6.50E-03 | 1.17 |
| C1QA | 5.38E-03 | 1.17 |
| CALD1 | 1.05E-02 | 1.17 |
| CYBB | 4.53E-03 | 1.17 |
| DDIT4 | 4.24E-04 | 1.17 |
| TNFRSF10B | 2.31E-04 | 1.17 |
| CD80 | 6.29E-03 | 1.16 |
| DCBLD1 | 3.99E-03 | 1.16 |
| HOMER3 | 6.38E-03 | 1.16 |
| ITGA6 | 1.06E-02 | 1.16 |
| MICAL2 | 2.06E-02 | 1.16 |
| PRIM2B | 6.44E-04 | 1.16 |
| SH3TC1 | 5.68E-04 | 1.16 |
| ZNF114 | 9.96E-03 | 1.16 |
| DCBLD2 | 1.07E-02 | 1.15 |
| DLGAP5 | 5.46E-04 | 1.15 |
| RELB | 1.29E-04 | 1.15 |
| SLC11A1 | 7.25E-03 | 1.15 |
| SLC39A8 | 6.92E-03 | 1.15 |
| TCP11L1 | 3.88E-03 | 1.15 |
| FTH1 | 1.43E-05 | 1.14 |
| FYB | 7.09E-03 | 1.14 |
| FZD2 | 2.38E-04 | 1.14 |
| KIAA0226L | 2.65E-03 | 1.14 |
| PLEC | 7.60E-04 | 1.14 |
| TMEM268 | 2.79E-04 | 1.14 |
| USP18 | 3.83E-02 | 1.14 |
| USP31 | 4.97E-03 | 1.14 |
| COL4A2 | 7.99E-03 | 1.13 |
| GADD45B | 8.41E-04 | 1.13 |
| LAIR1 | 4.59E-03 | 1.13 |
| OLR1 | 2.85E-02 | 1.13 |
| TLR8 | 8.51E-03 | 1.13 |
| VMP1 | 1.93E-05 | 1.13 |
| XPR1 | 1.49E-05 | 1.13 |
| ANXA3 | 2.79E-02 | 1.12 |
| DUSP10 | 4.48E-04 | 1.12 |
| LRRC8C | 1.65E-04 | 1.12 |
| PDP1 | 3.07E-05 | 1.12 |
| SPHK1 | 3.12E-03 | 1.12 |
| TGIF1 | 1.50E-05 | 1.12 |
| UHMK1 | 2.90E-03 | 1.12 |
| ARTN | 5.84E-03 | 1.11 |
| DKK3 | 1.85E-02 | 1.11 |
| KLRC2 | 4.88E-02 | 1.11 |
| MMP28 | 3.94E-02 | 1.11 |
| TNS4 | 5.15E-03 | 1.11 |
| CBS | 4.38E-02 | 1.1 |
| CEP55 | 4.86E-04 | 1.1 |
| IL15 | 9.82E-03 | 1.1 |
| ITGB2 | 4.51E-03 | 1.1 |
| NRP2 | 7.52E-04 | 1.1 |
| P4HA1 | 8.57E-05 | 1.1 |
| PHLDA1 | 6.26E-04 | 1.1 |
| STARD3NL | 1.34E-05 | 1.1 |
| TNC | 2.11E-02 | 1.1 |
| TPM1 | 1.51E-02 | 1.1 |
| ADAM8 | 2.79E-04 | 1.09 |
| ETS1 | 1.05E-03 | 1.09 |
| IRS1 | 8.70E-03 | 1.09 |
| LITAF | 3.95E-07 | 1.09 |
| LYN | 9.30E-04 | 1.09 |
| MCM10 | 7.74E-03 | 1.09 |
| ZNF281 | 1.01E-07 | 1.09 |
| BIRC5 | 8.57E-03 | 1.08 |
| IL1RAP | 1.98E-02 | 1.08 |
| RHOBTB3 | 7.31E-03 | 1.08 |
| SERINC2 | 1.57E-02 | 1.08 |
| SRGAP1 | 2.92E-04 | 1.08 |
| CDCA5 | 1.26E-03 | 1.07 |
| CTSZ | 4.65E-03 | 1.07 |
| FKBP11 | 4.68E-03 | 1.07 |
| LOXL2 | 2.92E-02 | 1.07 |
| SLC20A1 | 4.59E-04 | 1.07 |
| SNORA5B | 2.50E-04 | 1.07 |
| CDC42EP3 | 1.18E-03 | 1.06 |
| COL12A1 | 3.23E-02 | 1.06 |
| GPR160 | 4.95E-02 | 1.06 |
| GPR39 | 1.42E-02 | 1.06 |
| HMGB3 | 4.39E-04 | 1.06 |
| NEDD9 | 2.93E-04 | 1.06 |
| PRR5L | 8.03E-06 | 1.06 |
| SMIM3 | 1.82E-03 | 1.06 |
| WDR54 | 8.32E-04 | 1.06 |
| WISP1 | 4.41E-02 | 1.06 |
| BLM | 8.41E-04 | 1.05 |
| KIF26B | 4.83E-02 | 1.05 |
| PAG1 | 1.75E-04 | 1.05 |
| RTTN | 7.62E-03 | 1.05 |
| SLC39A14 | 2.83E-03 | 1.05 |
| STAT1 | 3.36E-02 | 1.05 |
| TOX | 1.80E-02 | 1.05 |
| HOTAIRM1 | 4.06E-03 | 1.04 |
| LPCAT1 | 4.12E-04 | 1.04 |
| MT2A | 2.78E-03 | 1.04 |
| NFE2L3 | 2.99E-02 | 1.04 |
| RAD51AP1 | 6.12E-04 | 1.04 |
| CMTM7 | 2.69E-03 | 1.03 |
| COLGALT1 | 1.45E-04 | 1.03 |
| CTSL | 3.31E-04 | 1.03 |
| FAM92A1 | 1.54E-04 | 1.03 |
| IGF2BP2 | 1.22E-02 | 1.03 |
| LRRC8A | 7.03E-04 | 1.03 |
| MMP19 | 1.73E-02 | 1.03 |
| MS4A4A | 1.78E-02 | 1.03 |
| PPP4R4 | 2.76E-02 | 1.03 |
| TIMP1 | 3.89E-03 | 1.03 |
| C3AR1 | 1.30E-03 | 1.02 |
| CD300LF | 5.44E-03 | 1.02 |
| DRAM1 | 1.77E-04 | 1.02 |
| FOXM1 | 6.15E-03 | 1.02 |
| FPR3 | 1.74E-02 | 1.02 |
| ITGA5 | 1.43E-02 | 1.02 |
| SGPP1 | 9.84E-03 | 1.02 |
| TANC2 | 9.88E-03 | 1.02 |
| UBE2S | 1.49E-04 | 1.02 |
| AP2B1 | 8.76E-04 | 1.01 |
| IGSF6 | 4.80E-03 | 1.01 |
| PXN | 8.58E-04 | 1.01 |
| RHOB | 4.92E-02 | 1.01 |
| RNF19B | 3.58E-04 | 1.01 |
| CCDC80 | 2.01E-02 | -1.01 |
| PLBD1 | 1.30E-02 | -1.01 |
| RAB3D | 6.25E-04 | -1.01 |
| RAB7B | 2.27E-03 | -1.01 |
| SLC9A9 | 3.08E-03 | -1.01 |
| VSIG10 | 7.45E-03 | -1.01 |
| ZNF521 | 2.18E-02 | -1.01 |
| APCDD1 | 3.50E-03 | -1.02 |
| KLF4 | 4.78E-02 | -1.02 |
| LINC00537 | 1.04E-02 | -1.02 |
| MMRN2 | 1.77E-04 | -1.02 |
| PNPLA3 | 2.10E-04 | -1.02 |
| BMP7 | 3.51E-02 | -1.03 |
| CDH19 | 3.15E-03 | -1.03 |
| CPEB2 | 2.65E-04 | -1.03 |
| CXCR2 | 1.77E-02 | -1.03 |
| DHRS1 | 3.30E-03 | -1.03 |
| GGTA1P | 9.52E-03 | -1.03 |
| GPLD1 | 1.22E-03 | -1.03 |
| HSPB6 | 2.15E-03 | -1.03 |
| KAT2B | 1.05E-03 | -1.03 |
| KLK14 | 2.95E-02 | -1.03 |
| MFSD6 | 1.02E-04 | -1.03 |
| MRLN | 1.05E-02 | -1.03 |
| OSBPL6 | 5.47E-03 | -1.03 |
| PRDX2 | 2.80E-04 | -1.03 |
| RANBP9 | 2.00E-04 | -1.03 |
| ZNF662 | 1.28E-04 | -1.03 |
| CBR1 | 5.25E-04 | -1.04 |
| CD1A | 3.42E-02 | -1.04 |
| CYBRD1 | 1.61E-03 | -1.04 |
| DNASE1L2 | 4.14E-08 | -1.04 |
| ECM2 | 1.67E-02 | -1.04 |
| FAM162A | 1.24E-05 | -1.04 |
| HTR3A | 4.92E-03 | -1.04 |
| METTL7A | 3.78E-02 | -1.04 |
| MRGPRF | 6.00E-03 | -1.04 |
| NOD2 | 8.98E-03 | -1.04 |
| PCOLCE2 | 3.44E-02 | -1.04 |
| PIGN | 4.39E-04 | -1.04 |
| SDR42E1 | 6.47E-05 | -1.04 |
| STOX2 | 9.55E-03 | -1.04 |
| TMOD4 | 2.14E-02 | -1.04 |
| FBXL16 | 1.60E-02 | -1.05 |
| FNDC5 | 3.08E-04 | -1.05 |
| GIPC2 | 8.47E-03 | -1.05 |
| GPD1L | 3.47E-02 | -1.05 |
| LDB2 | 2.21E-03 | -1.05 |
| PTGR1 | 1.81E-02 | -1.05 |
| RALGPS2 | 3.28E-04 | -1.05 |
| USP2 | 4.72E-03 | -1.05 |
| ACVR2A | 3.08E-03 | -1.06 |
| APOBEC2 | 3.12E-02 | -1.06 |
| DKK2 | 3.39E-05 | -1.06 |
| IL22RA1 | 6.74E-04 | -1.06 |
| KCNT2 | 1.23E-04 | -1.06 |
| KIAA1211L | 2.53E-02 | -1.06 |
| MAN1C1 | 1.67E-03 | -1.06 |
| OLFML1 | 1.30E-02 | -1.06 |
| PPFIBP2 | 2.77E-03 | -1.06 |
| PRSS2 | 4.31E-03 | -1.06 |
| RBP7 | 1.49E-02 | -1.06 |
| ACACB | 4.82E-03 | -1.07 |
| ACADSB | 2.61E-03 | -1.07 |
| CARD14 | 2.18E-05 | -1.07 |
| FDFT1 | 1.86E-04 | -1.07 |
| KLHL18 | 4.97E-07 | -1.07 |
| NOVA1 | 7.72E-05 | -1.07 |
| TCAIM | 4.88E-06 | -1.07 |
| ADIPOQ | 2.47E-03 | -1.08 |
| BDH1 | 1.27E-02 | -1.08 |
| BLNK | 3.58E-02 | -1.08 |
| EFNA5 | 6.06E-04 | -1.08 |
| FGF7 | 1.69E-04 | -1.08 |
| KY | 9.61E-04 | -1.08 |
| MAB21L3 | 1.06E-02 | -1.08 |
| MYRIP | 1.88E-04 | -1.08 |
| QSOX1 | 7.92E-05 | -1.08 |
| RRAGD | 1.53E-02 | -1.08 |
| SCAPER | 3.99E-04 | -1.08 |
| TINCR | 3.16E-02 | -1.08 |
| TRIM7 | 4.46E-03 | -1.08 |
| ANKRD22 | 1.56E-02 | -1.09 |
| CHODL | 1.69E-05 | -1.09 |
| CIART | 1.22E-02 | -1.09 |
| CPXM2 | 6.49E-03 | -1.09 |
| FAM167A | 4.00E-03 | -1.09 |
| IDE | 2.76E-03 | -1.09 |
| ITGA9 | 4.35E-03 | -1.09 |
| P2RX7 | 3.69E-03 | -1.09 |
| ZNF254 | 3.76E-03 | -1.09 |
| CA13 | 1.38E-02 | -1.1 |
| DKFZP564C152 | 1.27E-05 | -1.1 |
| LONRF1 | 3.41E-04 | -1.1 |
| OSR1 | 5.19E-06 | -1.1 |
| CNKSR3 | 4.26E-05 | -1.11 |
| DIO2 | 2.74E-02 | -1.11 |
| NOV | 1.60E-02 | -1.11 |
| PLCXD1 | 7.29E-04 | -1.11 |
| SHE | 1.47E-03 | -1.11 |
| SLIT3 | 9.83E-07 | -1.11 |
| TMEM237 | 5.38E-03 | -1.11 |
| ADAM23 | 3.53E-02 | -1.12 |
| DSC3 | 1.13E-02 | -1.12 |
| FBLN1 | 1.20E-02 | -1.12 |
| MIB2 | 7.64E-09 | -1.12 |
| PGAM2 | 1.76E-03 | -1.12 |
| PGLYRP4 | 4.95E-02 | -1.12 |
| TCEA3 | 3.53E-02 | -1.12 |
| EBF1 | 1.20E-02 | -1.13 |
| FYCO1 | 6.50E-08 | -1.13 |
| HSPA4L | 6.46E-03 | -1.13 |
| KLHL31 | 1.69E-02 | -1.13 |
| NAV3 | 3.56E-02 | -1.13 |
| TNFRSF19 | 3.40E-02 | -1.13 |
| EDNRB | 5.98E-03 | -1.14 |
| EP300-AS1 | 1.75E-03 | -1.14 |
| KLF8 | 1.86E-03 | -1.14 |
| MANSC1 | 1.15E-02 | -1.14 |
| PITX1 | 3.05E-02 | -1.14 |
| PLA2G4A | 4.55E-02 | -1.14 |
| SLC5A1 | 2.00E-02 | -1.14 |
| SMYD1 | 4.94E-03 | -1.14 |
| TTN | 4.47E-02 | -1.14 |
| AMPD1 | 1.90E-02 | -1.15 |
| FAM189A2 | 1.68E-04 | -1.15 |
| ACTN2 | 1.93E-02 | -1.16 |
| ADSSL1 | 1.71E-02 | -1.16 |
| C1orf116 | 2.13E-02 | -1.16 |
| FRZB | 1.82E-02 | -1.16 |
| MYH7 | 6.95E-03 | -1.16 |
| SH3RF2 | 2.59E-03 | -1.16 |
| SNED1 | 2.04E-03 | -1.16 |
| TEF | 3.55E-04 | -1.16 |
| XKRX | 1.23E-04 | -1.16 |
| DIRAS3 | 6.24E-03 | -1.17 |
| PCSK6 | 6.72E-03 | -1.17 |
| PRSS3P2 | 1.37E-03 | -1.17 |
| ZNF273 | 9.84E-07 | -1.17 |
| ACKR4 | 5.87E-03 | -1.18 |
| COX7A1 | 1.14E-02 | -1.18 |
| DIAPH2 | 1.47E-04 | -1.18 |
| ESRG | 1.02E-02 | -1.18 |
| NIPAL2 | 5.69E-05 | -1.18 |
| TMEM100 | 5.21E-05 | -1.18 |
| TUBB2A | 4.45E-03 | -1.18 |
| ALS2CL | 7.57E-05 | -1.19 |
| GLIDR | 6.62E-04 | -1.19 |
| GLTP | 1.61E-03 | -1.19 |
| MYL3 | 1.45E-02 | -1.19 |
| PAIP2B | 9.37E-04 | -1.19 |
| PKP1 | 4.34E-02 | -1.19 |
| PLCD1 | 1.41E-05 | -1.19 |
| TYRO3 | 5.80E-05 | -1.19 |
| C1QTNF7 | 2.68E-05 | -1.2 |
| IFFO2 | 2.72E-04 | -1.2 |
| UNC13B | 4.19E-05 | -1.2 |
| C1orf68 | 2.23E-04 | -1.21 |
| C6orf132 | 2.59E-03 | -1.21 |
| CAMSAP3 | 2.30E-04 | -1.21 |
| CKMT2 | 2.15E-02 | -1.21 |
| CTSG | 2.28E-03 | -1.21 |
| GALNT16 | 4.23E-07 | -1.21 |
| PID1 | 3.97E-04 | -1.21 |
| RCAN2 | 5.33E-03 | -1.21 |
| XK | 1.00E-02 | -1.21 |
| DBI | 4.32E-07 | -1.22 |
| LYVE1 | 7.26E-03 | -1.22 |
| SELP | 4.61E-03 | -1.22 |
| HMGCR | 3.40E-06 | -1.23 |
| MYH11 | 3.33E-02 | -1.23 |
| NDN | 1.20E-02 | -1.23 |
| RAPGEFL1 | 1.30E-02 | -1.23 |
| SERPINA12 | 1.30E-03 | -1.23 |
| ADGRL2 | 4.76E-04 | -1.24 |
| EPHX2 | 4.28E-03 | -1.24 |
| LIMCH1 | 1.56E-02 | -1.24 |
| MSX1 | 3.35E-05 | -1.24 |
| NRN1 | 2.08E-03 | -1.24 |
| ROBO2 | 3.54E-04 | -1.24 |
| RUNX1T1 | 1.35E-02 | -1.24 |
| SLC24A3 | 1.01E-02 | -1.24 |
| CNTNAP3B | 3.35E-05 | -1.25 |
| EPN3 | 4.72E-04 | -1.25 |
| GAN | 3.78E-05 | -1.25 |
| LEPROT | 9.50E-03 | -1.25 |
| PLN | 4.32E-02 | -1.25 |
| YOD1 | 6.70E-05 | -1.25 |
| PITPNM3 | 3.15E-04 | -1.26 |
| PROS1 | 4.61E-04 | -1.26 |
| SERTAD4 | 1.96E-04 | -1.26 |
| UNC93A | 1.43E-04 | -1.26 |
| ALOXE3 | 2.41E-02 | -1.27 |
| CAPN6 | 2.07E-03 | -1.27 |
| GRHL1 | 2.26E-02 | -1.27 |
| LYPD3 | 7.46E-03 | -1.27 |
| PKIA | 1.65E-02 | -1.27 |
| THSD4 | 8.49E-06 | -1.27 |
| CLU | 4.73E-02 | -1.28 |
| COL21A1 | 1.93E-02 | -1.28 |
| FXYD1 | 1.01E-03 | -1.28 |
| HSPB8 | 1.27E-02 | -1.28 |
| MAPK13 | 1.88E-04 | -1.28 |
| PRSS12 | 9.48E-03 | -1.28 |
| C1orf21 | 6.77E-06 | -1.29 |
| CYP3A5 | 1.73E-02 | -1.29 |
| MPZL2 | 1.68E-04 | -1.29 |
| TMEM40 | 3.30E-03 | -1.29 |
| AHNAK | 1.47E-03 | -1.3 |
| ARHGAP32 | 1.16E-03 | -1.3 |
| CGNL1 | 4.31E-02 | -1.3 |
| PPT2 | 7.65E-07 | -1.3 |
| PTK6 | 5.60E-05 | -1.3 |
| TRDN | 1.18E-02 | -1.3 |
| XG | 2.69E-03 | -1.3 |
| AOC3 | 1.23E-03 | -1.31 |
| C1QTNF2 | 1.71E-10 | -1.31 |
| CASQ1 | 1.89E-03 | -1.31 |
| CDHR1 | 1.49E-03 | -1.31 |
| DEGS2 | 1.41E-04 | -1.31 |
| EPB41L4A | 3.90E-04 | -1.31 |
| EPHB6 | 2.33E-05 | -1.31 |
| FAM43A | 4.27E-03 | -1.31 |
| SORBS2 | 1.96E-04 | -1.31 |
| SOX6 | 1.07E-02 | -1.31 |
| TYRP1 | 5.70E-03 | -1.31 |
| ADAP2 | 7.72E-05 | -1.32 |
| DCT | 9.16E-06 | -1.32 |
| DUOX1 | 8.58E-03 | -1.32 |
| AIF1L | 1.21E-02 | -1.33 |
| CAB39L | 1.53E-04 | -1.33 |
| ZSCAN31 | 3.23E-03 | -1.33 |
| CHL1 | 4.28E-02 | -1.34 |
| GAS7 | 2.75E-04 | -1.34 |
| GFOD2 | 1.46E-05 | -1.34 |
| TMEM45A | 9.75E-03 | -1.34 |
| CXCL12 | 1.73E-02 | -1.35 |
| EVPL | 9.94E-04 | -1.35 |
| HYMAI | 1.63E-05 | -1.35 |
| LINC00592 | 2.22E-03 | -1.35 |
| PBX1 | 1.87E-02 | -1.35 |
| SORBS1 | 9.02E-03 | -1.35 |
| ULK3 | 8.93E-06 | -1.35 |
| HAUS7 | 3.86E-06 | -1.36 |
| ISM1 | 2.65E-03 | -1.36 |
| NLRX1 | 9.16E-06 | -1.36 |
| SLC15A1 | 2.04E-04 | -1.36 |
| CALML3 | 1.48E-03 | -1.37 |
| RAB25 | 2.63E-03 | -1.37 |
| GLDN | 8.69E-06 | -1.38 |
| IL36A | 2.22E-02 | -1.38 |
| NRAP | 4.38E-02 | -1.38 |
| PPP2R2C | 1.10E-02 | -1.38 |
| RNF39 | 8.18E-03 | -1.38 |
| SYNGR1 | 2.43E-04 | -1.38 |
| CBR3 | 1.90E-04 | -1.39 |
| HPSE | 3.12E-03 | -1.39 |
| MARCH3 | 4.38E-04 | -1.39 |
| MPP7 | 2.61E-03 | -1.39 |
| PANK1 | 1.25E-05 | -1.39 |
| PPID | 8.42E-05 | -1.39 |
| SCN4B | 4.35E-04 | -1.39 |
| SGCG | 2.26E-03 | -1.39 |
| AATBC | 1.18E-05 | -1.4 |
| BDNF | 7.15E-06 | -1.4 |
| FCHSD1 | 4.54E-06 | -1.4 |
| HPGD | 4.85E-02 | -1.4 |
| IVL | 4.72E-02 | -1.4 |
| ITIH5 | 1.88E-04 | -1.41 |
| MAF | 7.81E-05 | -1.41 |
| ADRA2A | 9.61E-03 | -1.42 |
| GPX3 | 5.92E-03 | -1.42 |
| PLAGL1 | 1.91E-04 | -1.42 |
| SLC16A9 | 2.13E-02 | -1.42 |
| SLC39A2 | 3.10E-03 | -1.42 |
| CLN8 | 3.46E-07 | -1.43 |
| FRY | 2.12E-04 | -1.43 |
| KLK9 | 1.10E-03 | -1.43 |
| RECK | 1.61E-04 | -1.43 |
| RGS5 | 3.75E-04 | -1.43 |
| SLC47A2 | 1.93E-03 | -1.43 |
| CA3 | 3.13E-02 | -1.44 |
| DCLK1 | 4.30E-06 | -1.44 |
| IL17RD | 6.06E-04 | -1.45 |
| OMD | 1.59E-02 | -1.45 |
| TP53AIP1 | 9.13E-04 | -1.45 |
| UPK1A | 4.88E-05 | -1.45 |
| BOC | 3.68E-05 | -1.46 |
| PGM5 | 5.18E-05 | -1.46 |
| C8orf22 | 5.40E-09 | -1.47 |
| PITX2 | 4.68E-03 | -1.47 |
| SEMA3G | 1.22E-06 | -1.47 |
| SH3BGRL2 | 1.56E-02 | -1.47 |
| CRLF1 | 7.86E-06 | -1.48 |
| HMCN1 | 3.96E-03 | -1.48 |
| IL20RA | 3.36E-04 | -1.48 |
| LDB3 | 2.28E-02 | -1.48 |
| MGLL | 9.74E-05 | -1.48 |
| PTGS1 | 1.35E-03 | -1.48 |
| BICDL2 | 6.54E-04 | -1.49 |
| GREM2 | 1.23E-04 | -1.49 |
| SULT2B1 | 1.04E-02 | -1.49 |
| ZNF185 | 4.48E-04 | -1.49 |
| GGT6 | 4.59E-04 | -1.5 |
| MEOX2 | 1.49E-03 | -1.5 |
| PEG3 | 2.70E-02 | -1.5 |
| TPPP | 8.22E-04 | -1.5 |
| ATP13A4 | 4.60E-03 | -1.51 |
| PERP | 2.87E-03 | -1.51 |
| TTC22 | 1.55E-04 | -1.51 |
| ALDH3B2 | 9.50E-03 | -1.52 |
| CERS3 | 1.59E-03 | -1.52 |
| CRYAB | 4.68E-03 | -1.52 |
| WNT2B | 5.88E-03 | -1.52 |
| ELMOD1 | 2.59E-04 | -1.53 |
| ID4 | 1.17E-04 | -1.53 |
| JAM2 | 5.45E-05 | -1.53 |
| ME1 | 4.30E-04 | -1.53 |
| PLA2G4B | 2.16E-05 | -1.53 |
| CRABP2 | 1.52E-03 | -1.54 |
| GRAMD1C | 9.60E-04 | -1.54 |
| IGF1 | 1.26E-02 | -1.54 |
| MIR99AHG | 5.98E-05 | -1.54 |
| ZDHHC11B | 5.97E-05 | -1.54 |
| ZNF750 | 5.06E-03 | -1.54 |
| ITM2A | 1.74E-04 | -1.55 |
| SUSD4 | 6.02E-03 | -1.55 |
| CXCL14 | 6.74E-03 | -1.56 |
| HYAL1 | 1.07E-04 | -1.56 |
| ANKRD29 | 2.66E-03 | -1.57 |
| C14orf132 | 3.62E-05 | -1.57 |
| DLX5 | 7.97E-04 | -1.57 |
| NDNF | 2.06E-07 | -1.57 |
| PTGFR | 2.10E-04 | -1.57 |
| SASH1 | 1.85E-06 | -1.58 |
| TMEM79 | 1.15E-03 | -1.58 |
| CDON | 2.57E-06 | -1.59 |
| FAM46B | 6.29E-03 | -1.59 |
| SPTLC3 | 2.69E-04 | -1.59 |
| ZDHHC21 | 3.70E-05 | -1.59 |
| ANXA9 | 1.62E-02 | -1.6 |
| FAM83C | 1.68E-02 | -1.6 |
| NPR3 | 4.50E-03 | -1.6 |
| WISP2 | 4.03E-03 | -1.6 |
| LTB4R | 3.24E-04 | -1.61 |
| MYZAP | 6.05E-04 | -1.61 |
| SPRR3 | 4.41E-02 | -1.61 |
| ELF5 | 2.94E-02 | -1.62 |
| GRHL3 | 7.44E-03 | -1.62 |
| GSTM3 | 2.27E-02 | -1.62 |
| PENK | 1.32E-02 | -1.62 |
| TMEM184A | 3.78E-05 | -1.62 |
| YIF1B | 3.80E-03 | -1.62 |
| AQP1 | 2.47E-06 | -1.63 |
| ATP2A1 | 9.12E-03 | -1.63 |
| DLX3 | 7.53E-05 | -1.63 |
| PALMD | 1.03E-05 | -1.63 |
| CYP4F12 | 4.59E-04 | -1.64 |
| EMCN | 2.18E-05 | -1.64 |
| KRT31 | 6.88E-04 | -1.64 |
| P2RY1 | 3.30E-03 | -1.64 |
| SPNS2 | 4.84E-04 | -1.64 |
| BSPRY | 3.17E-04 | -1.65 |
| CLDN11 | 3.35E-05 | -1.65 |
| BNIPL | 3.58E-05 | -1.66 |
| ABLIM1 | 2.29E-05 | -1.67 |
| CYP4B1 | 3.51E-02 | -1.67 |
| MPZL3 | 1.16E-04 | -1.67 |
| SCNN1A | 2.36E-02 | -1.67 |
| SNX21 | 4.91E-06 | -1.67 |
| TMPRSS13 | 2.11E-05 | -1.67 |
| FHL1 | 6.38E-03 | -1.68 |
| SOX7 | 1.52E-05 | -1.68 |
| SPRR2B | 2.89E-02 | -1.69 |
| TTC39A | 2.38E-04 | -1.69 |
| CSTB | 1.53E-03 | -1.71 |
| LINC01133 | 9.08E-03 | -1.71 |
| SOSTDC1 | 2.18E-02 | -1.73 |
| ARHGEF26 | 7.03E-03 | -1.74 |
| DGAT2 | 7.93E-04 | -1.74 |
| GDA | 1.63E-02 | -1.74 |
| GSTA4 | 1.81E-05 | -1.74 |
| CYP2C9 | 3.38E-07 | -1.75 |
| ELOVL7 | 6.61E-04 | -1.75 |
| MAB21L2 | 9.57E-05 | -1.75 |
| PPL | 6.41E-04 | -1.75 |
| PPM1L | 2.03E-04 | -1.75 |
| STX19 | 4.23E-07 | -1.75 |
| IGSF10 | 3.25E-05 | -1.76 |
| LGALSL | 1.59E-04 | -1.76 |
| PTGIS | 3.55E-03 | -1.76 |
| CFD | 4.33E-05 | -1.78 |
| DNASE1L3 | 2.96E-03 | -1.78 |
| EPHX3 | 2.11E-02 | -1.78 |
| LAMB4 | 4.66E-09 | -1.78 |
| PLAC9 | 4.69E-07 | -1.78 |
| IL36RN | 1.29E-02 | -1.79 |
| TGM1 | 2.20E-02 | -1.79 |
| ZBTB7C | 8.70E-05 | -1.79 |
| PLA2G3 | 4.59E-07 | -1.8 |
| FAM13C | 9.02E-06 | -1.81 |
| KLK11 | 1.02E-02 | -1.81 |
| KLK8 | 2.29E-02 | -1.81 |
| RIMS3 | 3.11E-04 | -1.81 |
| SCNN1B | 8.29E-05 | -1.81 |
| SH3GL3 | 9.42E-04 | -1.81 |
| ANKRD35 | 3.70E-05 | -1.82 |
| SERPINB4 | 3.13E-02 | -1.82 |
| ACKR1 | 3.75E-03 | -1.83 |
| LINC01279 | 1.03E-02 | -1.83 |
| LYPD5 | 5.93E-04 | -1.83 |
| ELN | 2.39E-04 | -1.84 |
| LRRN4CL | 6.66E-08 | -1.84 |
| NDRG4 | 7.15E-06 | -1.84 |
| SERPINB3 | 2.72E-02 | -1.84 |
| SLC16A6 | 1.84E-05 | -1.84 |
| PRELP | 2.58E-05 | -1.85 |
| PRSS27 | 1.51E-02 | -1.85 |
| DLK1 | 1.06E-03 | -1.86 |
| FCER1A | 1.40E-03 | -1.86 |
| NOS1 | 2.36E-05 | -1.86 |
| SMIM5 | 5.85E-05 | -1.87 |
| GPC3 | 6.09E-04 | -1.88 |
| MAP2 | 2.00E-03 | -1.88 |
| AKR1B10 | 1.54E-02 | -1.9 |
| KRT80 | 1.23E-03 | -1.9 |
| CLIC3 | 5.65E-03 | -1.91 |
| DMKN | 1.92E-02 | -1.91 |
| ESYT3 | 2.01E-07 | -1.91 |
| ALOX12 | 3.72E-03 | -1.92 |
| PYGM | 1.94E-03 | -1.92 |
| SLC16A14 | 1.11E-04 | -1.92 |
| CARD18 | 7.60E-03 | -1.93 |
| SFRP1 | 9.75E-03 | -1.94 |
| CNFN | 3.42E-02 | -1.95 |
| ALDH3A1 | 3.05E-02 | -1.96 |
| ABI3BP | 7.62E-03 | -1.97 |
| PLP1 | 4.92E-08 | -1.97 |
| TPRG1 | 4.79E-03 | -1.98 |
| KLK10 | 9.78E-03 | -1.99 |
| SLC16A7 | 8.38E-07 | -1.99 |
| NMU | 1.20E-03 | -2.01 |
| WFDC5 | 2.86E-03 | -2.01 |
| RAET1E | 1.34E-04 | -2.02 |
| FOXP2 | 5.24E-04 | -2.03 |
| PSORS1C2 | 1.02E-03 | -2.03 |
| ATP6V1C2 | 1.38E-04 | -2.04 |
| SCEL | 2.50E-02 | -2.05 |
| TMEM45B | 2.09E-03 | -2.05 |
| FAM84A | 3.35E-05 | -2.06 |
| SPAG17 | 1.23E-07 | -2.06 |
| ATG9B | 1.34E-05 | -2.07 |
| BBOX1 | 1.10E-03 | -2.07 |
| CCL15 | 1.88E-04 | -2.08 |
| KRT10 | 2.47E-04 | -2.08 |
| ADH7 | 1.48E-02 | -2.1 |
| CYSRT1 | 8.02E-03 | -2.1 |
| PPP1R3C | 3.35E-03 | -2.12 |
| CD207 | 2.01E-05 | -2.13 |
| GDF10 | 2.65E-09 | -2.13 |
| SERPINB12 | 1.78E-10 | -2.13 |
| ANK2 | 5.76E-04 | -2.14 |
| MFAP4 | 2.79E-04 | -2.14 |
| TGM5 | 2.18E-05 | -2.14 |
| HOPX | 8.64E-04 | -2.15 |
| OGN | 2.78E-03 | -2.15 |
| POF1B | 1.05E-03 | -2.15 |
| RORA | 1.44E-08 | -2.15 |
| ABCA12 | 1.02E-03 | -2.16 |
| GDPD3 | 4.38E-04 | -2.16 |
| KLK12 | 1.44E-02 | -2.18 |
| HRASLS | 1.08E-04 | -2.2 |
| A2ML1 | 6.09E-04 | -2.21 |
| NEBL | 1.77E-05 | -2.21 |
| TM7SF2 | 8.94E-05 | -2.22 |
| GBP6 | 1.47E-03 | -2.23 |
| PTGER3 | 4.72E-09 | -2.24 |
| TGFBR3 | 2.88E-06 | -2.24 |
| ADH1B | 2.24E-05 | -2.25 |
| FOXN1 | 4.73E-04 | -2.25 |
| SBSN | 1.28E-02 | -2.25 |
| TMPRSS11D | 2.85E-03 | -2.25 |
| RHCG | 1.86E-02 | -2.26 |
| SCARA5 | 1.86E-04 | -2.29 |
| AADAC | 1.01E-07 | -2.3 |
| LINC00302 | 1.48E-04 | -2.3 |
| KLK7 | 1.41E-02 | -2.31 |
| PRSS3 | 4.56E-04 | -2.31 |
| CYP4F22 | 4.52E-06 | -2.33 |
| REEP1 | 3.35E-05 | -2.33 |
| EXPH5 | 1.82E-06 | -2.34 |
| ATP10B | 2.02E-05 | -2.35 |
| DPT | 2.89E-05 | -2.35 |
| ASPRV1 | 2.54E-02 | -2.41 |
| CRCT1 | 1.09E-02 | -2.41 |
| SDR9C7 | 3.78E-04 | -2.41 |
| MAMDC2 | 2.24E-05 | -2.46 |
| SPRR2C | 6.35E-03 | -2.47 |
| ACPP | 9.62E-06 | -2.48 |
| COMP | 1.19E-03 | -2.48 |
| C10orf99 | 5.69E-03 | -2.49 |
| SPINK6 | 2.14E-02 | -2.49 |
| TPPP3 | 1.12E-04 | -2.49 |
| ABCA8 | 4.70E-04 | -2.51 |
| DSC1 | 3.84E-02 | -2.51 |
| HLF | 1.97E-05 | -2.52 |
| SERPINB13 | 1.15E-03 | -2.52 |
| NSG1 | 1.63E-04 | -2.53 |
| SPINK5 | 5.26E-03 | -2.57 |
| CILP | 2.76E-05 | -2.58 |
| KRT9 | 3.74E-05 | -2.59 |
| LCE1B | 2.44E-04 | -2.59 |
| CYP2C18 | 5.85E-04 | -2.6 |
| CAPNS2 | 3.41E-05 | -2.66 |
| CLDN17 | 5.19E-06 | -2.68 |
| ELOVL4 | 4.47E-06 | -2.68 |
| WFDC12 | 3.76E-03 | -2.69 |
| CALML5 | 2.22E-03 | -2.74 |
| KLK13 | 5.26E-03 | -2.74 |
| VSIG10L | 2.41E-04 | -2.74 |
| HAL | 2.71E-03 | -2.75 |
| DSC2 | 3.06E-04 | -2.77 |
| CHRDL1 | 2.07E-04 | -2.8 |
| COL14A1 | 4.69E-07 | -2.81 |
| MUC15 | 4.04E-05 | -2.81 |
| KRT78 | 5.48E-04 | -2.83 |
| RDH12 | 1.50E-04 | -2.83 |
| LY6G6C | 1.05E-06 | -2.84 |
| KRT13 | 3.88E-02 | -2.85 |
| LCE3D | 5.88E-03 | -2.85 |
| SERPINB11 | 8.60E-05 | -2.85 |
| CD36 | 5.30E-09 | -2.87 |
| EXOSC7 | 1.44E-08 | -2.87 |
| FLG2 | 4.52E-03 | -2.92 |
| DAPL1 | 6.58E-04 | -3.05 |
| ALOX12B | 2.61E-04 | -3.1 |
| CWH43 | 3.39E-05 | -3.11 |
| ANGPTL1 | 8.50E-08 | -3.12 |
| SPRR2G | 7.97E-03 | -3.13 |
| TNXB | 3.96E-09 | -3.18 |
| KRT2 | 1.60E-04 | -3.25 |
| CDSN | 2.50E-04 | -3.26 |
| MAL | 5.81E-03 | -3.3 |
| TMPRSS11B | 8.08E-03 | -3.38 |
| ENDOU | 1.90E-06 | -3.42 |
| RPTN | 6.99E-04 | -3.53 |
| AADACL2 | 1.12E-07 | -3.55 |
| SLURP1 | 1.15E-03 | -3.6 |
| LCE2B | 7.38E-06 | -3.66 |
| DSG1 | 1.09E-03 | -3.67 |
| BPIFC | 5.22E-09 | -3.69 |
| CRNN | 9.72E-03 | -3.79 |
| SPINK7 | 6.40E-04 | -3.85 |
| ARG1 | 4.10E-06 | -3.98 |
| FLG | 2.91E-05 | -4.06 |
| KRT76 | 3.49E-07 | -4.15 |
| TGM3 | 4.01E-04 | -4.16 |
| LOR | 1.19E-05 | -4.33 |
| KRT1 | 4.93E-04 | -4.69 |

DEG, differentially expressed genes; FDR, false discovery rate; FC, fold change.

**Supplementary Table 3.** A total of 3271 DEGs were identified between oral squamous cell carcinoma and oral healthy tissue based on the RNA-sequencing dataset GSE186775.

| **Symbol** | **FDR** | **log2 FC** |
| --- | --- | --- |
| DNAH17-AS1 | 7.76E-12 | 5.6778962 |
| S100A7A | 1.24E-07 | 5.62244494 |
| SLCO1B3 | 4.65E-10 | 5.61466264 |
| MMP13 | 8.11E-11 | 5.56304478 |
| SLCO1B3-SLCO1B7 | 2.36E-10 | 5.5386815 |
| MMP10 | 4.18E-08 | 5.37631396 |
| LCAL1 | 2.39E-11 | 5.32922353 |
| SPP1 | 1.25E-11 | 5.21983488 |
| CREG2 | 8.11E-11 | 5.21663718 |
| CASP14 | 2.63E-06 | 5.10107077 |
| LINC00668 | 1.70E-11 | 4.92587993 |
| CNTNAP2 | 4.14E-10 | 4.90545581 |
| LINC01160 | 2.64E-12 | 4.84101786 |
| KRT74 | 6.96E-06 | 4.79254237 |
| MMP1 | 1.72E-07 | 4.71935873 |
| DSC1 | 8.59E-08 | 4.68432527 |
| HOXC11 | 1.08E-08 | 4.63910929 |
| PTHLH | 2.79E-13 | 4.53607214 |
| INHBA | 7.59E-14 | 4.51518354 |
| CDSN | 3.76E-07 | 4.45084349 |
| DUXAP10 | 3.15E-14 | 4.42017316 |
| WFDC12 | 2.13E-05 | 4.36202863 |
| SH2D5 | 1.36E-10 | 4.32351219 |
| LAMC2 | 1.74E-11 | 4.29265801 |
| KRT17 | 1.20E-08 | 4.24963145 |
| DUXAP9 | 3.25E-14 | 4.24894074 |
| NEFL | 1.47E-07 | 4.20926984 |
| AMTN | 4.20E-05 | 4.19814202 |
| PLAC1 | 6.31E-06 | 4.18460389 |
| LCE3D | 2.20E-04 | 4.17245029 |
| HMGA2 | 1.50E-07 | 4.12898373 |
| DUXAP8 | 8.03E-13 | 4.06428157 |
| WNT7A | 4.94E-07 | 4.06075394 |
| PNPLA1 | 1.30E-06 | 4.0431404 |
| WTAPP1 | 1.53E-09 | 4.02950025 |
| COL11A1 | 5.79E-07 | 4.02231568 |
| EN1 | 9.37E-10 | 4.01858452 |
| TNC | 9.37E-12 | 4.01355907 |
| DNAH17 | 3.25E-14 | 3.95779596 |
| PPP4R4 | 4.82E-13 | 3.95679609 |
| IL11 | 4.62E-07 | 3.95185303 |
| HOXC13-AS | 3.46E-05 | 3.91769348 |
| SERPINE1 | 1.36E-09 | 3.91465931 |
| MMP12 | 4.13E-09 | 3.90441882 |
| KRT42P | 2.85E-06 | 3.8627739 |
| ACP7 | 2.54E-06 | 3.86132441 |
| CSMD2 | 5.41E-09 | 3.86014937 |
| IFI6 | 2.56E-13 | 3.81178805 |
| KLHDC7B | 5.44E-08 | 3.80796485 |
| ISG15 | 7.25E-10 | 3.75749192 |
| MMP11 | 2.27E-10 | 3.69442977 |
| IGHGP | 1.19E-04 | 3.67122964 |
| TUBB3 | 2.06E-11 | 3.65681395 |
| TGFBI | 3.25E-14 | 3.61738624 |
| KRT75 | 9.46E-04 | 3.61714754 |
| LCE3E | 4.73E-04 | 3.61587598 |
| SLC7A11 | 2.57E-06 | 3.61377883 |
| TCHH | 1.98E-04 | 3.59666132 |
| TRPV3 | 7.44E-09 | 3.56898438 |
| EPIC1 | 7.31E-06 | 3.56265495 |
| RBP1 | 2.39E-11 | 3.55437833 |
| S100A7 | 1.90E-03 | 3.53106236 |
| ASPRV1 | 1.03E-05 | 3.51966842 |
| ADAM12 | 7.45E-12 | 3.50877135 |
| ZNF114 | 6.10E-13 | 3.5084049 |
| PAX5 | 1.02E-07 | 3.5070742 |
| STEAP1B | 2.28E-07 | 3.49588207 |
| MIR31HG | 1.74E-08 | 3.48270327 |
| NELL2 | 1.66E-06 | 3.47438932 |
| RAB3B | 5.66E-06 | 3.44871406 |
| EPO | 9.68E-06 | 3.4420845 |
| FER1L6 | 2.31E-09 | 3.44064391 |
| SPOCD1 | 2.39E-11 | 3.422775 |
| HMGA2-AS1 | 3.21E-08 | 3.41177858 |
| MYOSLID | 2.29E-09 | 3.40154822 |
| TENM2 | 6.49E-10 | 3.39735217 |
| LAMA1 | 2.38E-08 | 3.38803094 |
| ARTN | 3.20E-09 | 3.38384192 |
| CALB1 | 3.99E-07 | 3.36307867 |
| GPRIN1 | 1.84E-12 | 3.36214016 |
| LEMD1 | 9.50E-07 | 3.36077243 |
| MMP9 | 6.53E-06 | 3.35240847 |
| UCN2 | 1.51E-07 | 3.33278947 |
| CXCL11 | 3.43E-05 | 3.32274352 |
| SPATA22 | 6.15E-09 | 3.32235588 |
| CNGB1 | 4.43E-04 | 3.31812747 |
| KRT77 | 1.02E-03 | 3.31744681 |
| HOXC13 | 1.58E-03 | 3.30375449 |
| RSAD2 | 5.72E-07 | 3.30178205 |
| RPE65 | 1.23E-03 | 3.27168628 |
| ZAN | 8.10E-07 | 3.27005023 |
| CHST2 | 1.24E-06 | 3.26567883 |
| RPLP0P2 | 6.19E-07 | 3.26151771 |
| CYP27C1 | 2.29E-09 | 3.2577766 |
| HOXD10 | 2.06E-04 | 3.25739408 |
| SLC12A5-AS1 | 3.03E-06 | 3.24644585 |
| CELSR3 | 1.97E-11 | 3.2288852 |
| KRT16 | 1.65E-04 | 3.21798368 |
| RIMS2 | 1.42E-06 | 3.21340749 |
| LINC01615 | 2.38E-08 | 3.21238628 |
| LINC01559 | 1.74E-04 | 3.2095278 |
| ULBP2 | 2.34E-07 | 3.20779975 |
| RNASE7 | 1.57E-05 | 3.18569159 |
| KRT6B | 1.46E-04 | 3.16735913 |
| MFAP2 | 2.14E-12 | 3.16377023 |
| PI3 | 1.95E-03 | 3.15784926 |
| KLK5 | 7.41E-05 | 3.1566079 |
| SCAT1 | 3.67E-05 | 3.15565139 |
| GBP5 | 1.38E-09 | 3.14637458 |
| TM4SF19-AS1 | 4.23E-08 | 3.13736266 |
| KRTDAP | 2.69E-03 | 3.13542093 |
| DHRS2 | 9.89E-06 | 3.12658296 |
| SYT12 | 7.99E-09 | 3.10512633 |
| KLK14 | 5.16E-04 | 3.09943356 |
| TM4SF19 | 9.50E-07 | 3.09750523 |
| ALOXE3 | 1.46E-04 | 3.09054606 |
| HEPHL1 | 3.50E-04 | 3.08133028 |
| BBOX1-AS1 | 1.20E-09 | 3.07602444 |
| CFAP251 | 2.74E-13 | 3.07208622 |
| TSPAN10 | 2.62E-08 | 3.06094397 |
| CHP2 | 7.97E-04 | 3.03622585 |
| CXCL10 | 6.49E-05 | 3.02911396 |
| NMRAL2P | 4.16E-05 | 3.02168 |
| RDH16 | 2.93E-08 | 3.01816693 |
| PLEKHG4B | 7.05E-06 | 3.01662563 |
| TREM2 | 6.00E-07 | 3.00123367 |
| LINC01527 | 1.47E-03 | 2.99750175 |
| KRT17P2 | 5.13E-05 | 2.99512349 |
| PLAU | 1.32E-10 | 2.98654224 |
| KLHDC7B-DT | 2.84E-06 | 2.98137314 |
| VNN3P | 6.27E-04 | 2.97689814 |
| KLK6 | 9.27E-04 | 2.96895447 |
| ELAVL2 | 5.73E-06 | 2.93817001 |
| IGF2BP2 | 1.41E-13 | 2.92892867 |
| TM4SF19-DYNLT2B | 2.40E-07 | 2.92212082 |
| CCDC190 | 1.11E-03 | 2.9094603 |
| C10orf55 | 1.59E-10 | 2.90730771 |
| ZFPM2-AS1 | 4.39E-06 | 2.89879977 |
| COL22A1 | 1.18E-05 | 2.88707138 |
| COL4A6 | 4.47E-11 | 2.88615594 |
| ABCA12 | 3.05E-04 | 2.87207494 |
| C9 | 1.08E-04 | 2.86815761 |
| CTSV | 1.21E-05 | 2.86680904 |
| FBN2 | 2.22E-05 | 2.86190556 |
| MMP3 | 2.89E-04 | 2.85053221 |
| IL24 | 1.10E-04 | 2.84618857 |
| WFDC5 | 2.78E-04 | 2.82714158 |
| LINC01605 | 9.04E-10 | 2.82630058 |
| MISP | 2.30E-04 | 2.82518565 |
| GRIN2D | 6.87E-07 | 2.8251647 |
| PGLYRP4 | 9.84E-04 | 2.81788176 |
| AOC1 | 1.60E-04 | 2.81469708 |
| POSTN | 1.43E-06 | 2.81018377 |
| HOXC8 | 4.61E-05 | 2.80954535 |
| KRT14 | 1.35E-04 | 2.79614938 |
| IDO1 | 2.10E-05 | 2.79356761 |
| MATN3 | 1.43E-06 | 2.79149108 |
| TMEM132A | 1.29E-09 | 2.78892104 |
| FADD | 7.39E-11 | 2.78618745 |
| BCAT1 | 2.49E-09 | 2.78517733 |
| IGFL3 | 2.20E-03 | 2.78207191 |
| MYO3B | 1.59E-08 | 2.77768491 |
| CPXM1 | 5.78E-06 | 2.75965722 |
| KCNMB2-AS1 | 1.58E-06 | 2.75364894 |
| ALOX12B | 5.28E-03 | 2.74770856 |
| GPR39 | 2.18E-09 | 2.74194668 |
| SLC6A2 | 3.30E-05 | 2.73899793 |
| LOXL2 | 5.59E-08 | 2.7332223 |
| LAMP5 | 2.97E-06 | 2.73045636 |
| RGS4 | 1.02E-05 | 2.72556838 |
| KIF26B | 1.26E-07 | 2.71733574 |
| CALML5 | 6.33E-03 | 2.71301496 |
| CYP27B1 | 6.27E-08 | 2.71167826 |
| CDKN2A | 8.52E-05 | 2.70903545 |
| SERPINB7 | 8.62E-03 | 2.70102567 |
| FLRT3 | 1.61E-07 | 2.69973035 |
| PSORS1C1 | 2.62E-09 | 2.69625286 |
| ONECUT2 | 2.07E-05 | 2.69134684 |
| SPRR4 | 1.79E-02 | 2.68322562 |
| PLA2G7 | 8.11E-09 | 2.68081497 |
| CCDC87 | 3.05E-11 | 2.67879075 |
| CACNG4 | 1.02E-04 | 2.67585311 |
| KC6 | 6.24E-03 | 2.66741068 |
| FHAD1 | 2.59E-06 | 2.6595916 |
| SDS | 1.75E-06 | 2.65098714 |
| IL36RN | 8.04E-03 | 2.65097709 |
| HOXC6 | 5.68E-05 | 2.64033179 |
| ADAMDEC1 | 8.97E-04 | 2.63791253 |
| IFNE | 2.64E-05 | 2.63669435 |
| OASL | 1.25E-04 | 2.63477869 |
| LINC00491 | 5.62E-03 | 2.63467897 |
| FAM167A | 3.42E-05 | 2.63401342 |
| CST6 | 2.06E-03 | 2.62153251 |
| SALL4 | 5.21E-05 | 2.61940701 |
| ULBP1 | 7.52E-04 | 2.61587703 |
| LINC00519 | 7.65E-06 | 2.6127936 |
| CERNA2 | 1.41E-03 | 2.60945954 |
| LYPD1 | 1.93E-08 | 2.600103 |
| MICAL2 | 2.50E-12 | 2.59063158 |
| FN1 | 1.16E-04 | 2.58284547 |
| CXCL9 | 3.46E-04 | 2.57761977 |
| LYPD5 | 6.15E-04 | 2.57570676 |
| FAP | 4.44E-06 | 2.57270524 |
| HSD17B6 | 1.58E-07 | 2.56669025 |
| GSDMA | 2.05E-03 | 2.56597149 |
| SLC28A3 | 2.49E-04 | 2.56505058 |
| MICALCL | 1.59E-07 | 2.56259561 |
| KLK7 | 2.50E-03 | 2.55966879 |
| MSLN | 7.84E-03 | 2.55495641 |
| CDH3 | 5.95E-05 | 2.55024745 |
| PNLIPRP3 | 6.11E-03 | 2.5439405 |
| ABCA13 | 4.23E-04 | 2.54215691 |
| HOXA1 | 1.35E-04 | 2.53876018 |
| KYNU | 6.35E-08 | 2.53499947 |
| PCSK9 | 3.61E-04 | 2.53442952 |
| EPHB2 | 5.40E-06 | 2.52661498 |
| NXPH4 | 2.23E-05 | 2.5261097 |
| GYG2P1 | 8.35E-05 | 2.52489801 |
| AJAP1 | 5.73E-05 | 2.51981298 |
| FSCN1 | 8.54E-08 | 2.51863082 |
| KRT16P3 | 2.48E-03 | 2.51375169 |
| APCDD1L-DT | 7.06E-04 | 2.51113918 |
| IGHV3-21 | 2.85E-02 | 2.50964884 |
| IL36G | 1.96E-02 | 2.50939454 |
| SLC28A3-AS1 | 3.59E-04 | 2.50831723 |
| TMEM45A | 3.57E-04 | 2.50567189 |
| ADTRP | 2.29E-09 | 2.50323722 |
| S100A12 | 5.79E-03 | 2.50311422 |
| KLK9 | 1.94E-02 | 2.50275046 |
| RGS20 | 1.29E-07 | 2.49406547 |
| VSTM2L | 3.82E-04 | 2.48002187 |
| SCNN1D | 2.19E-05 | 2.47433061 |
| ARSI | 1.59E-04 | 2.46734755 |
| SERPINE2 | 7.00E-07 | 2.4670221 |
| IGF2BP3 | 1.54E-04 | 2.46420439 |
| PMEPA1 | 3.61E-07 | 2.46349924 |
| COL7A1 | 2.39E-06 | 2.46330075 |
| SLC7A8 | 6.76E-09 | 2.45739508 |
| ADAMTS2 | 1.12E-05 | 2.45269006 |
| RUNDC3A | 2.28E-05 | 2.44988564 |
| PLEK2 | 1.19E-05 | 2.44676217 |
| COL1A1 | 5.36E-06 | 2.44148509 |
| COL12A1 | 1.64E-06 | 2.43764071 |
| MUC16 | 4.79E-03 | 2.43754418 |
| BST2 | 2.09E-05 | 2.43675905 |
| DKK1 | 9.78E-05 | 2.43526798 |
| SLC44A5 | 7.13E-05 | 2.43460629 |
| LY6K | 5.20E-07 | 2.43139917 |
| PTPRH | 2.30E-04 | 2.42633904 |
| P3H2 | 1.03E-05 | 2.42063682 |
| NMB | 5.18E-10 | 2.41798004 |
| IFIT1 | 7.33E-06 | 2.41458309 |
| KRT6C | 9.12E-03 | 2.40850245 |
| C5orf34-AS1 | 2.94E-06 | 2.40617825 |
| IGFBP3 | 7.43E-05 | 2.40334474 |
| IGFL2-AS1 | 3.70E-03 | 2.39932756 |
| GJA1 | 3.11E-08 | 2.39797817 |
| GDPD2 | 2.26E-05 | 2.39314944 |
| SLC7A11-AS1 | 7.75E-04 | 2.38841652 |
| EDIL3 | 5.78E-06 | 2.38790113 |
| LAMA3 | 4.52E-10 | 2.38030425 |
| EGFR | 1.07E-05 | 2.37351019 |
| TNFRSF12A | 1.84E-08 | 2.3656871 |
| LINC00707 | 3.43E-04 | 2.36165379 |
| IFIT3 | 3.85E-07 | 2.35999143 |
| GJA3 | 2.86E-04 | 2.35799598 |
| CD177 | 1.02E-02 | 2.35643099 |
| ATP12A | 3.01E-02 | 2.3538207 |
| RASGEF1A | 2.17E-06 | 2.3521499 |
| TNFRSF9 | 1.15E-05 | 2.35214571 |
| GSDME | 1.18E-09 | 2.35133406 |
| PTK7 | 2.80E-09 | 2.35017681 |
| CCN6 | 3.29E-04 | 2.34539565 |
| MSC | 2.27E-04 | 2.3449091 |
| DCBLD1 | 3.07E-14 | 2.34462484 |
| KRT16P2 | 9.63E-03 | 2.34196632 |
| TDO2 | 3.47E-04 | 2.34043864 |
| LIPK | 2.50E-02 | 2.33736877 |
| DNAH5 | 2.11E-03 | 2.33604399 |
| ABCA4 | 5.68E-05 | 2.33251314 |
| C6orf141 | 6.89E-06 | 2.32515115 |
| KRT1 | 3.34E-02 | 2.32312572 |
| TMEM92 | 1.48E-04 | 2.32142663 |
| DDX60 | 2.50E-08 | 2.31668125 |
| ANO1 | 1.06E-04 | 2.31134481 |
| KRT16P1 | 2.44E-03 | 2.30856595 |
| KREMEN2 | 2.43E-04 | 2.30304546 |
| IL31RA | 3.15E-05 | 2.30075353 |
| CHST6 | 2.08E-03 | 2.29845448 |
| ADAMTS14 | 1.14E-05 | 2.29322687 |
| TYMP | 1.02E-05 | 2.29194922 |
| SORCS2 | 1.63E-07 | 2.29157007 |
| KRT34 | 1.25E-03 | 2.29154829 |
| EVA1A | 7.61E-04 | 2.29152797 |
| ULBP3 | 3.83E-07 | 2.29019213 |
| FERMT1 | 2.29E-05 | 2.28893831 |
| IGFL2 | 2.94E-03 | 2.28758594 |
| SHANK2 | 1.92E-03 | 2.28633931 |
| IGF2BP2-AS1 | 6.92E-07 | 2.27770482 |
| PFKFB4 | 2.84E-10 | 2.27538348 |
| COL4A5 | 7.59E-14 | 2.27135079 |
| CT69 | 1.72E-02 | 2.27089193 |
| MIR711 | 1.79E-05 | 2.27000525 |
| SYT7 | 1.68E-04 | 2.26575331 |
| FST | 1.03E-05 | 2.26476463 |
| IFI27 | 2.84E-05 | 2.2633692 |
| EGFR-AS1 | 2.13E-05 | 2.26053106 |
| KRT9 | 5.78E-03 | 2.25922375 |
| CDCA5 | 2.53E-05 | 2.2452169 |
| C1QTNF6 | 2.84E-07 | 2.24341506 |
| S100P | 1.43E-02 | 2.24271028 |
| SHISAL1 | 2.62E-04 | 2.24059022 |
| GPR176 | 2.99E-09 | 2.2391737 |
| SLC2A1 | 2.98E-04 | 2.22842785 |
| CPA6 | 5.57E-04 | 2.22752924 |
| LOX | 1.87E-07 | 2.22362303 |
| RIMS3 | 1.38E-07 | 2.22208288 |
| CHST11 | 2.14E-10 | 2.21656592 |
| LIPG | 4.83E-03 | 2.21408353 |
| EPSTI1 | 8.51E-08 | 2.20582083 |
| CCNA1 | 1.98E-04 | 2.20581477 |
| DPF1 | 7.48E-05 | 2.20514661 |
| PLA2G4E | 1.16E-02 | 2.20242763 |
| PICSAR | 5.76E-04 | 2.20067448 |
| TREX2 | 1.49E-03 | 2.19426385 |
| DLX6 | 4.32E-05 | 2.19271347 |
| NGF | 1.33E-06 | 2.19019244 |
| HTR7 | 2.01E-04 | 2.1888011 |
| AMIGO2 | 1.43E-07 | 2.18527756 |
| SERPINH1 | 1.57E-07 | 2.18402455 |
| DNMT3B | 1.32E-10 | 2.1832078 |
| STRA6 | 1.45E-04 | 2.17779883 |
| PCYT1B | 1.70E-03 | 2.17395767 |
| SNAI2 | 2.20E-11 | 2.17313816 |
| NRG1 | 5.99E-05 | 2.17028891 |
| CEACAM19 | 3.96E-05 | 2.16440289 |
| COL5A1 | 4.43E-05 | 2.16364624 |
| FABP5 | 2.95E-03 | 2.16363036 |
| MYBL2 | 2.89E-04 | 2.16234043 |
| ARL14 | 1.04E-02 | 2.16176134 |
| DISP2 | 5.78E-06 | 2.16130102 |
| TUBA4B | 5.56E-05 | 2.15958934 |
| LNCOC1 | 2.28E-07 | 2.15910138 |
| WNT10A | 1.17E-03 | 2.15728258 |
| DLX6-AS1 | 5.48E-05 | 2.15694392 |
| SLC38A5 | 5.40E-06 | 2.15225105 |
| OAS2 | 1.13E-05 | 2.14432076 |
| CDHR2 | 5.17E-06 | 2.1423195 |
| GPX2 | 3.58E-03 | 2.14175862 |
| PSPH | 1.26E-04 | 2.13810375 |
| IL12RB2 | 2.73E-08 | 2.13770451 |
| VEGFC | 1.31E-05 | 2.13659064 |
| KRT10 | 3.30E-03 | 2.13494573 |
| LPAR3 | 3.49E-04 | 2.13139269 |
| AKR1C2 | 1.38E-03 | 2.12830227 |
| NRIP3 | 1.22E-08 | 2.12784054 |
| FKBP9P1 | 2.32E-04 | 2.12582868 |
| LAMP3 | 3.65E-04 | 2.12582131 |
| HCG4 | 1.18E-04 | 2.1243028 |
| ZNF474 | 1.15E-04 | 2.11800192 |
| TTYH3 | 2.98E-11 | 2.11794554 |
| IGHG1 | 2.14E-02 | 2.1158213 |
| PSAPL1 | 4.58E-04 | 2.11418502 |
| LINC02159 | 3.50E-03 | 2.10817289 |
| CTHRC1 | 3.52E-04 | 2.10556336 |
| C16orf74 | 8.12E-05 | 2.10554977 |
| HLA-V | 1.62E-03 | 2.10471272 |
| ITGA3 | 2.39E-11 | 2.10356557 |
| LRRC15 | 2.04E-03 | 2.0934139 |
| PPFIA1 | 2.59E-08 | 2.09258494 |
| FAT1 | 5.36E-10 | 2.09207749 |
| NIPAL4 | 2.84E-03 | 2.08890439 |
| LINC01415 | 3.78E-07 | 2.08784186 |
| KRT79 | 2.31E-02 | 2.08699924 |
| B4GALNT3 | 2.53E-04 | 2.08049959 |
| POU3F1 | 2.48E-03 | 2.07859658 |
| CLSPN | 1.20E-04 | 2.07060208 |
| OCIAD2 | 1.33E-09 | 2.06700173 |
| LINC00958 | 2.21E-04 | 2.06295001 |
| CCT6A | 1.19E-04 | 2.06144191 |
| LINC00504 | 1.38E-03 | 2.06118606 |
| AKR1B10 | 8.54E-03 | 2.05401419 |
| TRIB3 | 4.72E-05 | 2.05305847 |
| FLRT2-AS1 | 3.85E-05 | 2.05147193 |
| CMPK2 | 1.49E-04 | 2.05014405 |
| PDPN | 2.84E-07 | 2.04919945 |
| ALG1L1P | 5.02E-07 | 2.04630791 |
| DLX2 | 2.21E-03 | 2.04553332 |
| DLX5 | 2.15E-05 | 2.04216167 |
| VAV2 | 2.78E-09 | 2.0413417 |
| WNT7B | 4.93E-03 | 2.03439086 |
| GPR15LG | 3.69E-02 | 2.02898958 |
| RAB31 | 2.53E-09 | 2.0289717 |
| SLC15A1 | 1.27E-02 | 2.02385526 |
| LYNX1-SLURP2 | 2.60E-02 | 2.02214073 |
| APOL1 | 1.05E-06 | 2.02123238 |
| CTTN | 5.74E-06 | 2.0187597 |
| SCO2 | 3.65E-06 | 2.01651331 |
| LINC02700 | 7.68E-03 | 2.01439376 |
| SNX10 | 6.19E-08 | 2.01334121 |
| LAYN | 9.99E-06 | 2.01240008 |
| IFI44L | 2.79E-05 | 2.00908615 |
| HES2 | 1.47E-02 | 2.0086369 |
| PTPRZ1 | 4.55E-04 | 2.00853763 |
| RAG1 | 5.19E-06 | 1.99759114 |
| PLOD2 | 7.21E-07 | 1.99717422 |
| NLRP2 | 4.87E-02 | 1.99274013 |
| ZSCAN12P1 | 5.56E-05 | 1.98378079 |
| AACSP1 | 2.89E-03 | 1.98377132 |
| LINC01094 | 3.07E-06 | 1.97945882 |
| SLC2A1-DT | 6.58E-05 | 1.97901083 |
| TNIP3 | 1.22E-02 | 1.97261103 |
| DHCR7 | 1.09E-05 | 1.96941543 |
| SUMF2 | 2.91E-04 | 1.96812494 |
| ADAMTS15 | 2.40E-04 | 1.96710909 |
| CTXN1 | 3.79E-06 | 1.95640216 |
| COL5A3 | 4.34E-05 | 1.95620142 |
| USP32P3 | 1.91E-05 | 1.95565271 |
| SMOX | 8.75E-09 | 1.95469152 |
| GGH | 1.15E-04 | 1.95197087 |
| KIAA1549L | 2.27E-04 | 1.95013164 |
| NUDT11 | 1.46E-05 | 1.94872541 |
| CMKLR2 | 4.12E-04 | 1.9468468 |
| EFNB1 | 5.26E-07 | 1.94670531 |
| NPY4R | 9.08E-04 | 1.94569561 |
| TEDC2 | 1.07E-05 | 1.94557524 |
| LINC01010 | 8.18E-04 | 1.94554804 |
| TNFRSF18 | 4.34E-04 | 1.94429631 |
| CCL20 | 4.24E-02 | 1.94188568 |
| IFI44 | 9.27E-06 | 1.93983349 |
| GJB6 | 1.78E-02 | 1.93936085 |
| CD80 | 1.20E-05 | 1.93760798 |
| GPR153 | 2.11E-09 | 1.93597482 |
| COL27A1 | 4.58E-08 | 1.93563585 |
| WARS1 | 2.07E-07 | 1.93360445 |
| FOXD1 | 2.02E-05 | 1.93295748 |
| FBXO2 | 9.19E-05 | 1.92623954 |
| SCAND3 | 2.95E-06 | 1.92622393 |
| MIR1182 | 1.23E-05 | 1.92598369 |
| KANK4 | 1.39E-02 | 1.92577524 |
| FBLIM1 | 1.34E-10 | 1.92048634 |
| OAS3 | 9.31E-06 | 1.91660528 |
| TMEM26 | 2.96E-04 | 1.91446976 |
| FADS1 | 1.80E-04 | 1.90985769 |
| BLACAT1 | 5.99E-05 | 1.90697635 |
| BMP2 | 1.17E-05 | 1.90656121 |
| MIR4713HG | 5.78E-03 | 1.9058309 |
| P3H4 | 9.58E-07 | 1.90573285 |
| EPB41L4B | 1.45E-04 | 1.90101487 |
| FAM83A | 2.09E-02 | 1.89867213 |
| COL4A1 | 2.86E-06 | 1.89362863 |
| TPBG | 1.37E-09 | 1.88829933 |
| GLDC | 6.06E-03 | 1.88701789 |
| CDK5R1 | 1.89E-04 | 1.88663059 |
| MYO1B | 1.87E-07 | 1.88616626 |
| OGDHL | 4.19E-02 | 1.88502374 |
| CDK6 | 9.24E-08 | 1.88368595 |
| LAMB3 | 1.94E-03 | 1.88148226 |
| TNFSF10 | 5.87E-07 | 1.88136367 |
| TENM3-AS1 | 3.87E-04 | 1.87667618 |
| CTSC | 1.10E-07 | 1.87415439 |
| IGHM | 2.59E-02 | 1.8735623 |
| GBP1 | 3.47E-05 | 1.87281502 |
| EGLN3 | 1.44E-03 | 1.86541026 |
| CDC20 | 1.40E-03 | 1.86539923 |
| IGKV3-15 | 4.45E-02 | 1.86517939 |
| LGALS7B | 3.68E-02 | 1.86334302 |
| PNPLA3 | 6.33E-04 | 1.86055333 |
| COL5A2 | 1.75E-05 | 1.85965472 |
| BFSP1 | 1.73E-08 | 1.85481483 |
| CXCL1 | 3.75E-03 | 1.85328561 |
| FOXM1 | 6.29E-04 | 1.8479101 |
| CARD17P | 1.11E-02 | 1.84646552 |
| BCL2L10 | 3.60E-03 | 1.8458957 |
| THBS2 | 1.59E-03 | 1.84547794 |
| GLI1 | 5.93E-04 | 1.84402772 |
| MYO10 | 2.90E-09 | 1.843678 |
| EPPK1 | 4.38E-03 | 1.84136128 |
| BATF2 | 2.40E-03 | 1.84017232 |
| LINC01426 | 4.36E-04 | 1.83957276 |
| NCF2 | 1.71E-11 | 1.83879548 |
| RTP4 | 2.50E-05 | 1.83570302 |
| SERPINA1 | 1.55E-05 | 1.83445281 |
| IFI30 | 6.59E-07 | 1.8341835 |
| GOLGA7B | 3.65E-05 | 1.834089 |
| SLC37A2 | 1.95E-04 | 1.83373259 |
| AGRN | 7.64E-11 | 1.8295468 |
| PLA2G4E-AS1 | 3.14E-02 | 1.81891979 |
| CDKN2B-AS1 | 9.31E-05 | 1.81541994 |
| PARP12 | 6.10E-13 | 1.81320761 |
| GLIS1 | 1.25E-03 | 1.81285952 |
| ETV4 | 7.73E-05 | 1.81048853 |
| STAT1 | 8.42E-07 | 1.81043126 |
| PRNP | 3.15E-09 | 1.80987249 |
| USB1 | 2.66E-11 | 1.8078398 |
| BIRC5 | 1.39E-03 | 1.80639757 |
| GPNMB | 3.76E-05 | 1.79926488 |
| KLK8 | 3.46E-02 | 1.79788893 |
| FEZ1 | 2.00E-04 | 1.79750411 |
| FAM89A | 7.17E-06 | 1.79707431 |
| TRIP13 | 5.81E-04 | 1.79131148 |
| IGHV4-34 | 4.96E-02 | 1.79097273 |
| LINC00696 | 1.06E-02 | 1.790184 |
| ARSJ | 2.56E-06 | 1.78785895 |
| FZD2 | 3.15E-07 | 1.78754458 |
| CDH11 | 1.43E-03 | 1.78743013 |
| PLK1 | 4.47E-04 | 1.78677905 |
| MIR4260 | 2.45E-03 | 1.78587688 |
| PLA2G4D | 3.61E-02 | 1.7843376 |
| ABCG4 | 1.07E-02 | 1.78415837 |
| UBE2C | 1.73E-03 | 1.78397193 |
| TGFA | 4.76E-04 | 1.78359447 |
| OLR1 | 2.97E-03 | 1.78348831 |
| CNTN1 | 1.53E-05 | 1.78332253 |
| MX2 | 1.37E-03 | 1.78117578 |
| GDF15 | 8.10E-03 | 1.7806418 |
| LINC02100 | 1.55E-03 | 1.77808471 |
| DDX60L | 8.48E-09 | 1.77700963 |
| RHEX | 8.74E-04 | 1.77657876 |
| STX1A | 1.52E-09 | 1.77652292 |
| P3H1 | 3.52E-06 | 1.77495783 |
| CERCAM | 1.98E-04 | 1.77492447 |
| MTHFD1L | 1.93E-08 | 1.77426031 |
| SDK2 | 3.35E-05 | 1.77335943 |
| CCR8 | 4.16E-03 | 1.76873558 |
| AJUBA | 3.46E-04 | 1.76622874 |
| ACP5 | 2.62E-07 | 1.76306608 |
| SQLE | 7.76E-04 | 1.76079099 |
| C1QTNF12 | 3.17E-03 | 1.75987736 |
| CEP55 | 1.67E-03 | 1.75967582 |
| KLK10 | 3.58E-02 | 1.75791944 |
| ITGA6 | 2.18E-07 | 1.75648633 |
| MMP17 | 2.17E-07 | 1.75489135 |
| FCGR3A | 6.25E-04 | 1.75476546 |
| ANKK1 | 1.48E-03 | 1.75381359 |
| ODC1 | 9.06E-04 | 1.7536335 |
| MMP14 | 3.91E-06 | 1.75128678 |
| PXDN | 1.46E-03 | 1.75068251 |
| BNC1 | 1.04E-02 | 1.7501884 |
| MYO5A | 6.60E-11 | 1.74920182 |
| ALDH3B2 | 3.10E-02 | 1.74905485 |
| PLXNA1 | 1.87E-10 | 1.74889751 |
| RAD51AP1 | 9.47E-05 | 1.74560217 |
| E2F7 | 1.18E-03 | 1.74488647 |
| PRSS23 | 3.99E-05 | 1.74434338 |
| RIGI | 3.99E-07 | 1.74386042 |
| FSTL3 | 1.26E-04 | 1.74336758 |
| ADAMTS7 | 1.36E-04 | 1.74279866 |
| XAF1 | 5.41E-05 | 1.73817036 |
| COX6B2 | 2.31E-03 | 1.73392042 |
| ASCL2 | 3.75E-03 | 1.73273218 |
| ASPHD2 | 7.95E-05 | 1.73149721 |
| PRAG1 | 4.15E-07 | 1.73063156 |
| BPGM | 2.00E-04 | 1.72907703 |
| ITPR3 | 2.20E-07 | 1.72900859 |
| KIF26B-AS1 | 3.08E-03 | 1.7273366 |
| MELK | 2.33E-03 | 1.72698898 |
| VOPP1 | 1.07E-04 | 1.72675251 |
| SIRPA | 4.35E-08 | 1.72670695 |
| S1PR5 | 1.12E-03 | 1.7225753 |
| MIR3606 | 2.44E-03 | 1.71897943 |
| ATP2C2 | 9.59E-04 | 1.71881373 |
| MDFI | 1.04E-04 | 1.71766536 |
| OVOS2 | 1.38E-03 | 1.71754314 |
| ADAMTS12 | 1.46E-04 | 1.71660023 |
| DSG2 | 5.35E-04 | 1.71332138 |
| TPCN2 | 2.54E-05 | 1.71052043 |
| IFIT2 | 1.56E-04 | 1.70823062 |
| COL4A2 | 4.04E-05 | 1.7077769 |
| FJX1 | 6.46E-04 | 1.70562935 |
| SULF2 | 1.58E-06 | 1.70484481 |
| CXCL14 | 3.94E-04 | 1.70442893 |
| P4HA2 | 1.10E-06 | 1.70425214 |
| ERVMER34-1 | 6.71E-03 | 1.70265288 |
| USP18 | 8.53E-07 | 1.69907396 |
| AIM2 | 2.58E-03 | 1.69871206 |
| COL17A1 | 9.99E-03 | 1.69713432 |
| COL4A2-AS1 | 3.53E-05 | 1.69633575 |
| PDZK1IP1 | 2.80E-02 | 1.69368602 |
| C2CD4A | 1.49E-02 | 1.69213311 |
| LY6E | 1.84E-06 | 1.69211024 |
| SCUBE3 | 3.90E-03 | 1.69147054 |
| SLC22A20P | 4.57E-03 | 1.69129685 |
| EDIL3-DT | 3.61E-04 | 1.69091192 |
| TMEM200A | 1.91E-03 | 1.68964555 |
| DGKI | 2.08E-03 | 1.6889723 |
| EEF1AKMT4 | 8.78E-06 | 1.68851208 |
| RASL11B | 3.76E-05 | 1.68828586 |
| UBE2L6 | 2.42E-08 | 1.68363919 |
| SLC39A6 | 4.81E-07 | 1.68341175 |
| RAB32 | 4.34E-07 | 1.68178053 |
| COL16A1 | 1.51E-07 | 1.68173599 |
| TAP1 | 1.65E-05 | 1.68123592 |
| GABRA3 | 2.47E-02 | 1.67909865 |
| SLC6A11 | 1.22E-02 | 1.6785943 |
| IFIH1 | 2.03E-05 | 1.6781614 |
| ADGRE1 | 1.28E-03 | 1.67645861 |
| SLC4A3 | 7.80E-10 | 1.67569852 |
| ICAM5 | 1.88E-04 | 1.67359804 |
| DLEU7 | 1.53E-06 | 1.67338616 |
| BEND6 | 2.77E-05 | 1.66927764 |
| MIR936 | 1.58E-02 | 1.66894248 |
| SLC52A2 | 3.34E-09 | 1.66893056 |
| ZNF469 | 7.81E-04 | 1.66643355 |
| RFX8 | 7.18E-03 | 1.66188063 |
| DLX3 | 2.00E-02 | 1.65964359 |
| TMC7 | 5.59E-08 | 1.65891745 |
| DUSP14 | 2.00E-05 | 1.65764963 |
| ALOX12P2 | 2.41E-05 | 1.65747163 |
| RDH12 | 3.16E-02 | 1.65697409 |
| PLAG1 | 1.38E-04 | 1.6551295 |
| RNFT2 | 5.61E-03 | 1.65498614 |
| PTGFRN | 1.96E-08 | 1.65472469 |
| TGFB1 | 1.81E-08 | 1.6546363 |
| KIF4A | 1.88E-03 | 1.65390395 |
| PI15 | 1.82E-03 | 1.65379156 |
| FAAP24 | 4.81E-09 | 1.65357187 |
| CGB7 | 1.37E-03 | 1.65110726 |
| ITGB4 | 7.00E-07 | 1.6487025 |
| LPCAT1 | 8.28E-09 | 1.64796847 |
| STC2 | 3.76E-03 | 1.64562442 |
| USP41 | 7.26E-05 | 1.64284059 |
| RDH10 | 1.02E-04 | 1.64063406 |
| ACOT7 | 1.05E-04 | 1.63996432 |
| NDRG4 | 2.53E-03 | 1.6377626 |
| CCNE1 | 2.65E-06 | 1.63744945 |
| PPIF | 4.13E-05 | 1.63716028 |
| FAM131C | 3.10E-03 | 1.63678903 |
| HKDC1 | 3.95E-03 | 1.63642986 |
| PPP1R14C | 1.17E-03 | 1.63242501 |
| COL3A1 | 3.53E-03 | 1.628636 |
| KLK1 | 1.76E-02 | 1.62111827 |
| MRPS17 | 3.48E-03 | 1.62038594 |
| PCDHB8 | 5.51E-04 | 1.62028806 |
| PPP2R2C | 1.75E-02 | 1.61975886 |
| PTPRK | 3.89E-09 | 1.61888931 |
| GPR68 | 4.74E-05 | 1.61816444 |
| RPL39L | 8.82E-04 | 1.61706536 |
| EEF1AKMT4-ECE2 | 2.95E-06 | 1.61659157 |
| H3C12 | 2.68E-02 | 1.61633331 |
| ITGA5 | 3.85E-05 | 1.615692 |
| ENAH | 1.49E-11 | 1.61476633 |
| GREM1 | 3.22E-03 | 1.61382525 |
| OLFM2 | 1.88E-04 | 1.61328815 |
| ATP1B3 | 3.84E-05 | 1.61199227 |
| PHF24 | 1.71E-03 | 1.61093552 |
| RN7SL3 | 2.01E-04 | 1.61049734 |
| CDCA8 | 1.34E-03 | 1.60984932 |
| CHST15 | 1.20E-09 | 1.60966111 |
| LINC02323 | 2.95E-02 | 1.60951626 |
| DSG2-AS1 | 1.48E-03 | 1.60779213 |
| SEC61G | 7.78E-04 | 1.60711793 |
| RPS29 | 4.10E-04 | 1.60679121 |
| SEMA7A | 4.40E-04 | 1.60641136 |
| LUCAT1 | 8.39E-03 | 1.60222169 |
| FFAR2 | 2.98E-03 | 1.60203244 |
| CDC25B | 3.94E-05 | 1.60190026 |
| DNAAF3 | 1.55E-03 | 1.60179484 |
| FAM86JP | 1.47E-05 | 1.60124025 |
| TLR2 | 5.17E-05 | 1.60076366 |
| MAGED4 | 1.35E-02 | 1.60068632 |
| CFAP58-DT | 1.64E-03 | 1.59956881 |
| MSR1 | 1.90E-03 | 1.59928605 |
| SAMD9 | 9.72E-03 | 1.59860748 |
| P2RY6 | 6.23E-03 | 1.59767364 |
| MAGED4B | 1.30E-02 | 1.59683349 |
| KIF2C | 1.02E-03 | 1.59596204 |
| FZD6 | 1.04E-06 | 1.59578011 |
| HCP5 | 2.00E-06 | 1.59372651 |
| HPCA | 3.42E-05 | 1.59299569 |
| PRR5L | 1.81E-05 | 1.59227168 |
| MSC-AS1 | 8.95E-04 | 1.58918006 |
| LINC02827 | 3.32E-03 | 1.58672453 |
| TMPRSS13 | 1.44E-02 | 1.58614316 |
| SLC6A15 | 1.32E-02 | 1.58508086 |
| HOTAIRM1 | 6.08E-04 | 1.58467041 |
| TNF | 1.00E-02 | 1.58457883 |
| ADGRG3 | 9.53E-08 | 1.5842555 |
| SOX11 | 3.58E-02 | 1.58424284 |
| MEFV | 2.27E-03 | 1.58327274 |
| H3C8 | 8.04E-03 | 1.5826527 |
| UHRF1 | 1.01E-03 | 1.58244765 |
| DCBLD2 | 1.21E-06 | 1.58120519 |
| P4HA2-AS1 | 1.65E-05 | 1.58112992 |
| IMPDH1 | 2.84E-11 | 1.58037545 |
| DLGAP5 | 5.76E-03 | 1.57862917 |
| ZNF713 | 2.47E-03 | 1.57808434 |
| EIF5A2 | 8.42E-07 | 1.57779349 |
| GNLY | 5.41E-03 | 1.57667751 |
| CDCA4 | 2.23E-05 | 1.57655092 |
| SLITRK6 | 3.03E-02 | 1.57579866 |
| CGAS | 2.74E-07 | 1.57357501 |
| RPS6KA4 | 2.03E-07 | 1.57234711 |
| XRCC2 | 9.76E-04 | 1.56858713 |
| LINC00319 | 7.15E-05 | 1.56698736 |
| CCDC144CP | 1.65E-03 | 1.5668742 |
| KIF18A | 1.18E-03 | 1.56673504 |
| ZBP1 | 8.40E-03 | 1.56659351 |
| AGTRAP | 2.46E-07 | 1.5661898 |
| ADGRG1 | 6.96E-06 | 1.56460035 |
| FAM111B | 2.80E-03 | 1.56043035 |
| ETV7 | 3.48E-03 | 1.55741401 |
| CDC6 | 1.84E-03 | 1.55653478 |
| TRIM36 | 1.26E-04 | 1.55476618 |
| BASP1 | 4.95E-05 | 1.55273685 |
| MEX3A | 1.03E-03 | 1.55137478 |
| HLA-J | 1.14E-03 | 1.54787506 |
| BMP1 | 8.40E-06 | 1.546756 |
| KAZN-AS1 | 3.38E-03 | 1.54633882 |
| AURKB | 1.00E-02 | 1.54409421 |
| KIF14 | 8.13E-03 | 1.54300921 |
| ADAMTS6 | 9.61E-04 | 1.54263198 |
| MSI1 | 3.19E-02 | 1.5419848 |
| PRELID3A | 5.83E-10 | 1.540399 |
| ANLN | 1.04E-02 | 1.5403079 |
| GINS4 | 2.63E-04 | 1.5336639 |
| PMFBP1 | 1.45E-03 | 1.5317642 |
| NCS1 | 2.97E-08 | 1.53005839 |
| LINC00964 | 9.06E-03 | 1.52945073 |
| TRPA1 | 1.78E-03 | 1.52896406 |
| CLDN14-AS1 | 1.76E-03 | 1.52841918 |
| VSIG8 | 4.54E-02 | 1.52831041 |
| GINS1 | 1.58E-03 | 1.52773612 |
| CD101 | 2.59E-07 | 1.52625389 |
| XCL1 | 1.27E-02 | 1.52440838 |
| RNF217-AS1 | 1.11E-09 | 1.52369374 |
| FLRT2 | 5.28E-04 | 1.52308086 |
| LINC01116 | 4.87E-04 | 1.52135546 |
| DEPDC1B | 2.46E-03 | 1.52103704 |
| MSX2 | 1.41E-03 | 1.52083225 |
| SNX10-AS1 | 8.17E-05 | 1.51924847 |
| ARPC1B | 6.67E-08 | 1.51916238 |
| SIRPB1 | 2.32E-06 | 1.51890366 |
| CENPA | 5.59E-03 | 1.51799335 |
| SLC3A2 | 3.15E-06 | 1.51524844 |
| ITGB6 | 7.35E-03 | 1.51484545 |
| PRR11 | 1.55E-03 | 1.51432461 |
| FOXD2-AS1 | 8.48E-06 | 1.51250581 |
| H2BC17 | 9.83E-03 | 1.51235935 |
| NWD2 | 3.49E-02 | 1.51191158 |
| RFLNB | 2.01E-04 | 1.51184637 |
| BRMS1 | 2.64E-09 | 1.51029504 |
| G6PD | 1.97E-03 | 1.50922888 |
| SIGMAR1 | 6.50E-06 | 1.50752958 |
| TPX2 | 4.96E-03 | 1.50742079 |
| IGSF3 | 1.81E-03 | 1.50674211 |
| CAV1 | 1.89E-04 | 1.50472604 |
| GPR84-AS1 | 7.43E-05 | 1.50463076 |
| SCRN1 | 8.29E-09 | 1.50430302 |
| KCNMB2 | 2.35E-03 | 1.50335427 |
| NOCT | 1.13E-05 | 1.50211392 |
| JAG1 | 5.46E-05 | 1.50173722 |
| PCDH7 | 5.89E-04 | 1.50072712 |
| CENPI | 1.01E-04 | 1.49982299 |
| SLC2A6 | 4.55E-05 | 1.49974614 |
| TMEM54 | 1.24E-03 | 1.49935226 |
| NLGN4X | 2.36E-02 | 1.49910892 |
| GALNT18 | 5.99E-13 | 1.49659751 |
| HIF1A-AS2 | 2.07E-05 | 1.49135179 |
| COL10A1 | 1.10E-03 | 1.49098331 |
| PTPRK-AS1 | 8.05E-08 | 1.49088583 |
| CHCHD2 | 3.13E-03 | 1.48994361 |
| CBX2 | 1.12E-03 | 1.4897722 |
| GPC6 | 3.71E-02 | 1.48917402 |
| MARCKSL1 | 9.14E-04 | 1.48880246 |
| HIF1A-AS1 | 8.56E-08 | 1.48854874 |
| ACTN1 | 2.74E-07 | 1.48849898 |
| GJB4 | 2.06E-02 | 1.4884638 |
| DSCAS | 3.70E-02 | 1.48732113 |
| PROCR | 2.80E-05 | 1.48716619 |
| SOCS1 | 5.11E-04 | 1.48657907 |
| SPHK1 | 8.66E-05 | 1.48605963 |
| THSD1 | 1.79E-07 | 1.48440677 |
| MOCOS | 9.98E-04 | 1.48439957 |
| AFAP1L2 | 7.16E-07 | 1.48428238 |
| E2F1 | 5.02E-05 | 1.48384129 |
| MET | 1.21E-06 | 1.48367897 |
| MUC12 | 1.20E-02 | 1.48367306 |
| TMEM158 | 1.26E-03 | 1.48295175 |
| LTO1 | 1.29E-04 | 1.48207645 |
| LRRC8D | 7.78E-06 | 1.48042631 |
| TMSB10 | 1.78E-09 | 1.47856293 |
| CD276 | 5.26E-06 | 1.47787752 |
| CDKN2B | 2.60E-02 | 1.47777412 |
| SPRY4 | 8.70E-07 | 1.47666956 |
| MSN | 6.85E-09 | 1.47538509 |
| POGLUT2 | 6.49E-05 | 1.47430615 |
| HLA-H | 5.40E-06 | 1.47422022 |
| ECT2 | 4.01E-05 | 1.47331988 |
| SLC52A1 | 1.11E-02 | 1.47254344 |
| GABRQ | 4.61E-02 | 1.47202143 |
| AURKA | 1.43E-04 | 1.47026397 |
| SLAMF7 | 3.17E-03 | 1.46945735 |
| SLC16A1 | 4.77E-04 | 1.46943445 |
| GTSE1 | 2.72E-03 | 1.46880804 |
| LINC00842 | 9.87E-03 | 1.46792323 |
| EXO1 | 7.02E-03 | 1.46558168 |
| FKBP10 | 9.88E-03 | 1.46539548 |
| HTATIP2 | 2.40E-07 | 1.46504459 |
| ADAP2 | 1.13E-05 | 1.46074108 |
| ZNF812P | 2.90E-02 | 1.46047223 |
| KLHL4 | 1.74E-02 | 1.45846911 |
| BCAS4 | 2.19E-04 | 1.45626649 |
| C1orf226 | 8.74E-03 | 1.45529236 |
| MIR6887 | 8.27E-03 | 1.45511614 |
| NOD2 | 1.03E-02 | 1.45420421 |
| HLA-B | 4.35E-06 | 1.45345777 |
| DKK3 | 2.29E-05 | 1.45339318 |
| SYNDIG1 | 2.99E-02 | 1.45190266 |
| LILRB4 | 9.09E-03 | 1.45146425 |
| GPR87 | 3.77E-02 | 1.45028385 |
| GJC1 | 1.42E-04 | 1.45016049 |
| ROR2 | 6.70E-03 | 1.4491046 |
| STON2 | 8.05E-06 | 1.44746872 |
| ADAM9 | 3.27E-05 | 1.44692967 |
| ASTN2-AS1 | 1.80E-03 | 1.44382185 |
| NDC80 | 5.83E-03 | 1.44343463 |
| CDCA2 | 1.33E-02 | 1.44244872 |
| IRX2-DT | 8.74E-03 | 1.44035199 |
| HMGB3 | 3.71E-06 | 1.43959465 |
| NAGS | 6.46E-05 | 1.43914509 |
| CDKN3 | 1.12E-03 | 1.43806417 |
| HLA-F | 9.48E-07 | 1.43648074 |
| FEN1 | 6.49E-05 | 1.43561926 |
| PIF1 | 3.70E-04 | 1.43500126 |
| PHEX | 4.26E-04 | 1.43418654 |
| COL4A2-AS2 | 4.77E-04 | 1.43417775 |
| KIF23 | 6.48E-03 | 1.43407473 |
| CLEC7A | 7.99E-03 | 1.43224757 |
| HELZ2 | 6.39E-05 | 1.43196494 |
| DLEU1-AS1 | 4.63E-03 | 1.43181425 |
| SLC47A2 | 2.54E-02 | 1.43055564 |
| NPY4R2 | 6.85E-03 | 1.4296535 |
| LYPD6B | 2.41E-02 | 1.42707523 |
| HJURP | 9.91E-03 | 1.42699486 |
| HLA-A | 2.76E-06 | 1.42599831 |
| FRMD8 | 7.39E-06 | 1.42552587 |
| MIR1915HG | 1.95E-03 | 1.42441984 |
| LUARIS | 5.77E-03 | 1.42097176 |
| F2RL1 | 2.74E-03 | 1.42078756 |
| SLC39A4 | 3.02E-05 | 1.41966397 |
| ENTPD7 | 1.52E-05 | 1.41906637 |
| PIK3CD | 1.12E-07 | 1.41898828 |
| COL8A1 | 3.48E-02 | 1.41871758 |
| SLC15A3 | 3.83E-07 | 1.41551052 |
| CCNF | 1.26E-04 | 1.41477508 |
| VANGL2 | 5.00E-05 | 1.41221224 |
| RNF17 | 1.00E-02 | 1.41186824 |
| KIF3C | 7.94E-07 | 1.41055201 |
| CHPF | 2.14E-04 | 1.40823823 |
| WDHD1 | 2.99E-04 | 1.40723843 |
| FBXO41 | 8.70E-05 | 1.4070863 |
| CCNJL | 1.64E-03 | 1.40447804 |
| MX1 | 7.94E-03 | 1.40373267 |
| APLN | 1.40E-02 | 1.40265805 |
| APOE | 1.82E-02 | 1.40091139 |
| PPP1R14B | 1.03E-04 | 1.39735826 |
| TTPAL | 7.45E-12 | 1.39708314 |
| NCAPH | 6.06E-03 | 1.39706741 |
| BCL11B | 7.09E-03 | 1.39275225 |
| CDH24 | 1.06E-06 | 1.39254759 |
| CASK | 2.42E-08 | 1.39121481 |
| GNA12 | 8.03E-07 | 1.39031817 |
| H3C3 | 1.93E-02 | 1.39003138 |
| HOMER3 | 1.28E-04 | 1.38981483 |
| FCRL3 | 4.15E-02 | 1.38778071 |
| PKMYT1 | 9.55E-05 | 1.38719152 |
| TANC2 | 1.04E-08 | 1.38144343 |
| NRCAM | 1.30E-02 | 1.38143568 |
| WDR54 | 2.59E-07 | 1.38037235 |
| ZP3 | 1.70E-04 | 1.37874983 |
| SUGCT | 1.13E-03 | 1.37861949 |
| PSMB8-AS1 | 1.12E-04 | 1.37757887 |
| CELSR2 | 3.92E-03 | 1.3774431 |
| HERC5 | 7.27E-06 | 1.37680043 |
| ASF1B | 1.03E-02 | 1.37602053 |
| FANCB | 2.15E-04 | 1.37540507 |
| MICALL1 | 2.39E-04 | 1.37407405 |
| PC | 4.35E-04 | 1.37337489 |
| H2AC16 | 1.91E-02 | 1.37334707 |
| MFHAS1 | 5.16E-07 | 1.37232279 |
| CDC27P11 | 4.28E-02 | 1.37209051 |
| SKA1 | 2.49E-02 | 1.36874241 |
| IL2RB | 4.33E-03 | 1.36732682 |
| CCL24 | 4.81E-02 | 1.36689648 |
| PYCARD | 2.04E-03 | 1.36646752 |
| SPINDOC | 4.96E-09 | 1.36467175 |
| KPNA2 | 2.28E-04 | 1.36257649 |
| TLL1 | 2.07E-02 | 1.3625129 |
| MEAK7 | 7.09E-04 | 1.36041252 |
| CFL1 | 6.67E-08 | 1.3601495 |
| PHLDB2 | 2.34E-04 | 1.35961074 |
| LINC01503 | 1.89E-04 | 1.35956768 |
| RAI14 | 1.30E-04 | 1.35933507 |
| SOX4 | 2.75E-05 | 1.35854779 |
| IER5L | 1.46E-05 | 1.35811767 |
| PDIA4 | 7.14E-06 | 1.35747731 |
| GRIA3 | 3.12E-02 | 1.35346397 |
| SP110 | 5.52E-06 | 1.35303913 |
| CDCA3 | 1.67E-03 | 1.35261148 |
| SLC9C1 | 1.61E-03 | 1.35195027 |
| NETO2 | 6.17E-03 | 1.3505899 |
| SH2D2A | 4.66E-04 | 1.35046408 |
| STK17A | 6.25E-07 | 1.34977437 |
| MIR671 | 3.19E-06 | 1.3467383 |
| ELFN1 | 8.41E-03 | 1.3464729 |
| SDK1 | 4.55E-03 | 1.3461454 |
| NAV1 | 3.65E-06 | 1.34565381 |
| MAP4K2 | 4.66E-07 | 1.34520283 |
| ORC1 | 8.65E-03 | 1.3451226 |
| PTGS1 | 3.94E-03 | 1.34385233 |
| TNFAIP3 | 1.26E-02 | 1.34376543 |
| TMEM265 | 1.39E-04 | 1.34286165 |
| SKA3 | 4.91E-03 | 1.34240555 |
| IL22RA1 | 3.09E-02 | 1.3410439 |
| MCM2 | 8.22E-04 | 1.34087994 |
| PARP14 | 3.22E-05 | 1.34067075 |
| PARP9 | 4.18E-04 | 1.33717413 |
| LINC01271 | 1.18E-04 | 1.33516535 |
| LTBP1 | 2.12E-04 | 1.33511902 |
| AHNAK2 | 8.30E-03 | 1.3342057 |
| ATP13A2 | 2.11E-04 | 1.33242678 |
| FLJ32255 | 2.58E-03 | 1.33199815 |
| KCNS1 | 1.03E-02 | 1.33100702 |
| MB21D2 | 5.52E-06 | 1.3292841 |
| GALNS | 6.22E-08 | 1.32883596 |
| PPP1R14B-AS1 | 4.29E-03 | 1.32765515 |
| HIF1A | 1.23E-04 | 1.32743621 |
| PCAT6 | 7.39E-03 | 1.32743399 |
| H2AC13 | 3.03E-02 | 1.32706414 |
| KPNA7 | 5.63E-03 | 1.32676435 |
| CXCR1 | 4.49E-02 | 1.32558308 |
| CCDC86 | 1.93E-05 | 1.32545007 |
| RMI2 | 3.71E-03 | 1.32371823 |
| CCL22 | 1.94E-02 | 1.32310097 |
| H2BC3 | 2.27E-02 | 1.32288551 |
| RHOD | 7.44E-03 | 1.32256726 |
| KRT10-AS1 | 2.42E-03 | 1.32191968 |
| IRF7 | 3.19E-04 | 1.32161147 |
| FAM241B | 2.10E-02 | 1.32130703 |
| TCEAL9 | 9.67E-10 | 1.32060565 |
| TK1 | 1.02E-03 | 1.32045848 |
| PRICKLE1 | 1.64E-04 | 1.32008322 |
| SPATA46 | 3.10E-02 | 1.32001183 |
| KHDC1 | 1.09E-07 | 1.31906847 |
| DEPDC1 | 2.32E-02 | 1.31857467 |
| OAS1 | 1.89E-02 | 1.31762219 |
| CFAP53 | 1.51E-03 | 1.31752803 |
| RAB38 | 3.03E-02 | 1.31688884 |
| PYCARD-AS1 | 3.13E-03 | 1.31556689 |
| HLA-C | 4.01E-06 | 1.31506116 |
| ABCC1 | 1.95E-04 | 1.3116438 |
| CAV2 | 1.41E-06 | 1.3078775 |
| LINC01679 | 7.72E-04 | 1.30767922 |
| GNB5 | 2.33E-11 | 1.30728616 |
| GLI2 | 2.17E-02 | 1.30615923 |
| KCNJ15 | 5.65E-04 | 1.30558638 |
| SERINC2 | 6.28E-03 | 1.30542601 |
| HOXD9 | 5.85E-03 | 1.3047771 |
| C12orf75 | 5.80E-03 | 1.30464269 |
| RNF217 | 6.69E-11 | 1.30412654 |
| H2BC11 | 4.73E-03 | 1.30382171 |
| LANCL2 | 4.95E-03 | 1.30364598 |
| ZSCAN31 | 3.15E-04 | 1.30312226 |
| CXCL8 | 4.13E-02 | 1.30269614 |
| TOX-DT | 2.47E-02 | 1.30264287 |
| CLXN | 1.03E-02 | 1.3020433 |
| KIFC3 | 4.51E-05 | 1.30156506 |
| CYB5R2 | 5.53E-04 | 1.30137398 |
| IRF9 | 1.21E-06 | 1.30122561 |
| H3C11 | 3.76E-02 | 1.30072109 |
| LRFN4 | 8.24E-06 | 1.29942059 |
| FBXO45 | 1.78E-05 | 1.29890087 |
| BMAL2 | 1.29E-02 | 1.29849233 |
| MEX3D | 5.26E-06 | 1.29714285 |
| SHCBP1 | 4.60E-03 | 1.29656458 |
| TPRG1-AS1 | 1.22E-02 | 1.29380129 |
| CDC45 | 1.44E-02 | 1.29321691 |
| H2BC12 | 1.62E-03 | 1.29142762 |
| PLOD3 | 5.39E-04 | 1.29093552 |
| MIR5090 | 1.17E-04 | 1.29070548 |
| LRP8 | 1.44E-02 | 1.29020119 |
| SLC38A7 | 1.36E-10 | 1.28900058 |
| PODNL1 | 1.30E-03 | 1.28898582 |
| CIB2 | 1.89E-04 | 1.28867983 |
| AQP9 | 1.79E-02 | 1.28852923 |
| RRAS2 | 3.78E-08 | 1.28783707 |
| GPAT4-AS1 | 1.52E-03 | 1.28679042 |
| HASPIN | 2.03E-03 | 1.28674956 |
| WDR53 | 2.97E-07 | 1.28646704 |
| MIR205 | 3.01E-02 | 1.28498158 |
| ITGB8 | 5.11E-04 | 1.28477424 |
| SRXN1 | 2.09E-03 | 1.28430836 |
| PYGL | 2.27E-03 | 1.28250641 |
| H2AC4 | 2.23E-02 | 1.28190244 |
| ALG3 | 3.54E-06 | 1.28019594 |
| PDLIM4 | 1.46E-04 | 1.28009681 |
| IL4I1 | 3.12E-07 | 1.27963002 |
| TFRC | 2.00E-04 | 1.2793422 |
| LINC02316 | 9.78E-03 | 1.2791291 |
| TP63 | 1.89E-02 | 1.27659873 |
| EML5 | 8.53E-05 | 1.27433298 |
| CD109 | 3.36E-05 | 1.27395214 |
| ITGAX | 6.96E-04 | 1.27343166 |
| KIF17 | 3.03E-03 | 1.2731055 |
| BGN | 8.70E-03 | 1.27259366 |
| TUBA1C | 3.50E-03 | 1.27131655 |
| GPC2 | 5.44E-04 | 1.27086102 |
| MIAT | 1.02E-02 | 1.27069388 |
| NID1 | 5.67E-03 | 1.27060696 |
| SLC2A9 | 1.15E-04 | 1.2694612 |
| RN7SL1 | 8.63E-04 | 1.2690776 |
| RAC3 | 1.81E-03 | 1.26878307 |
| SLC52A3 | 6.81E-04 | 1.26877529 |
| CCND2 | 1.04E-02 | 1.26811126 |
| MFSD10 | 1.59E-08 | 1.26734122 |
| TRAM2 | 7.60E-05 | 1.2669519 |
| ORC6 | 1.83E-03 | 1.26627915 |
| TREM1 | 1.53E-02 | 1.26411605 |
| SLC26A6 | 2.25E-07 | 1.26240837 |
| FRMD5 | 1.11E-02 | 1.26169742 |
| LINC01572 | 1.26E-04 | 1.26109379 |
| B3GNT7 | 1.71E-03 | 1.26025572 |
| TSHZ3 | 1.32E-04 | 1.25998399 |
| CCDC88B | 3.09E-04 | 1.258572 |
| ERCC6L | 3.19E-03 | 1.25768635 |
| LAPTM4B | 7.27E-05 | 1.25753905 |
| PYGB | 1.18E-04 | 1.25645018 |
| LURAP1L | 1.24E-02 | 1.25568379 |
| DPP3 | 9.54E-07 | 1.25563194 |
| SNORD86 | 3.28E-04 | 1.25521863 |
| LINC02966 | 9.09E-03 | 1.25489292 |
| SPECC1 | 5.66E-08 | 1.25413749 |
| LINC02678 | 2.46E-02 | 1.25398596 |
| STARD4 | 7.73E-05 | 1.25333378 |
| TMEM138 | 8.02E-11 | 1.25235996 |
| IGHMBP2 | 4.98E-04 | 1.25125378 |
| RNF128 | 2.08E-02 | 1.25013959 |
| FADS3 | 1.26E-03 | 1.24833492 |
| IRS1 | 5.69E-04 | 1.24737621 |
| APMAP | 1.46E-08 | 1.24669166 |
| YEATS2 | 1.10E-10 | 1.24549431 |
| TENM3 | 9.72E-03 | 1.24525383 |
| OLFML2A | 3.56E-03 | 1.2446449 |
| FAM43A | 5.44E-03 | 1.24330913 |
| PLA2G4F | 4.57E-02 | 1.24323219 |
| PCLAF | 2.14E-02 | 1.24315779 |
| NID2 | 2.22E-02 | 1.24241397 |
| ATAD2 | 1.27E-03 | 1.24111306 |
| ITGAV | 8.86E-07 | 1.24070877 |
| C3orf52 | 4.13E-03 | 1.24018066 |
| MTSS1 | 1.66E-05 | 1.23953982 |
| PLCXD2 | 1.58E-04 | 1.23875481 |
| H2BC9 | 2.72E-02 | 1.23861516 |
| RNA5-8SN4 | 3.07E-04 | 1.23835452 |
| NDRG1 | 1.71E-02 | 1.23786702 |
| AMMECR1 | 1.82E-03 | 1.2372354 |
| KIF18B | 4.70E-02 | 1.23681966 |
| STX3 | 2.62E-08 | 1.23668655 |
| RNA5-8SN3 | 1.08E-04 | 1.23621584 |
| MSL3P1 | 8.24E-03 | 1.23594894 |
| TMEM86A | 4.94E-03 | 1.23572657 |
| ST6GALNAC2 | 3.85E-04 | 1.23525646 |
| SEC14L2 | 2.50E-03 | 1.23495646 |
| TBILA | 4.02E-03 | 1.23278287 |
| SLC25A22 | 3.08E-05 | 1.2319654 |
| TOR4A | 1.34E-03 | 1.23158165 |
| ARHGAP11A-SCG5 | 3.38E-03 | 1.23157016 |
| TUSC3 | 5.16E-03 | 1.23125349 |
| PRSS53 | 2.30E-04 | 1.23064956 |
| ALDH1L2 | 2.43E-02 | 1.23012793 |
| FPR3 | 3.19E-03 | 1.22921665 |
| KMO | 7.99E-06 | 1.22903526 |
| CASP1P2 | 4.85E-02 | 1.22689856 |
| KRBA1 | 2.42E-06 | 1.22590478 |
| COLGALT1 | 6.35E-08 | 1.22532516 |
| BLOC1S3 | 3.99E-07 | 1.22514998 |
| UBASH3B | 1.19E-04 | 1.22504537 |
| RUNX1-IT1 | 1.88E-02 | 1.22485993 |
| UNC5B | 1.39E-02 | 1.22236494 |
| WNT6 | 2.10E-02 | 1.22021364 |
| MICB | 3.52E-03 | 1.21952487 |
| TOP1MT | 1.01E-06 | 1.21792186 |
| SKIL | 9.91E-08 | 1.21772225 |
| ZFP92 | 1.05E-02 | 1.21721434 |
| BLM | 1.85E-03 | 1.2164955 |
| FAM110A | 1.91E-03 | 1.21547174 |
| CD22 | 1.75E-03 | 1.21516726 |
| H3C7 | 2.72E-02 | 1.21422902 |
| BUB1 | 2.29E-02 | 1.21388897 |
| SPRED3 | 3.91E-04 | 1.21345626 |
| HTRA1 | 6.94E-03 | 1.21307072 |
| CKS2 | 5.53E-03 | 1.21297376 |
| SH3D21 | 1.63E-02 | 1.21185632 |
| CKAP2 | 2.69E-04 | 1.21098122 |
| TFAP2A-AS2 | 1.16E-02 | 1.21083546 |
| CD300LF | 6.97E-03 | 1.21077586 |
| NEIL3 | 2.29E-02 | 1.21009434 |
| RCC2 | 2.35E-04 | 1.20962336 |
| CKAP2L | 3.04E-02 | 1.20929602 |
| BEX3 | 6.10E-07 | 1.20843939 |
| MTCL1 | 3.60E-03 | 1.20812018 |
| RNASEH2A | 2.60E-04 | 1.20809107 |
| EPHB4 | 9.92E-06 | 1.20772282 |
| CENPW | 6.47E-03 | 1.20676328 |
| TOP2A | 4.23E-02 | 1.20618154 |
| LIMK1 | 2.82E-06 | 1.20567324 |
| FKBP14 | 6.84E-04 | 1.20562237 |
| LGALS3BP | 5.87E-06 | 1.20555752 |
| ENO2 | 2.74E-03 | 1.20536417 |
| MCM10 | 1.46E-02 | 1.20522645 |
| TMEM249 | 8.03E-06 | 1.2039246 |
| MDK | 9.72E-03 | 1.20356204 |
| DIAPH3 | 2.10E-02 | 1.20324419 |
| CENPE | 2.93E-02 | 1.20294511 |
| SMIM3 | 1.36E-03 | 1.20258675 |
| RUNX2 | 1.53E-03 | 1.20253456 |
| PLOD1 | 1.20E-04 | 1.20117352 |
| CYB561A3 | 2.56E-05 | 1.20115724 |
| H3C10 | 2.92E-02 | 1.2011236 |
| ASNS | 1.55E-03 | 1.20088943 |
| CALHM6 | 1.41E-02 | 1.20071776 |
| IFNLR1 | 1.40E-03 | 1.1988617 |
| FGF11 | 3.75E-02 | 1.19797407 |
| ASTN2 | 1.43E-02 | 1.19695195 |
| H2AC12 | 3.42E-02 | 1.19549131 |
| B4GALT4 | 5.93E-04 | 1.19510231 |
| EIF4EBP1 | 5.48E-04 | 1.19459875 |
| GALNT2 | 5.12E-07 | 1.19420477 |
| BAK1 | 3.78E-04 | 1.19328027 |
| NOX5 | 6.52E-03 | 1.19194852 |
| SHC1 | 5.70E-09 | 1.19189853 |
| ANKEF1 | 7.38E-05 | 1.19169473 |
| PSRC1 | 6.58E-05 | 1.19032589 |
| PCNX3 | 1.02E-05 | 1.19019023 |
| OTUB2 | 2.77E-03 | 1.18993028 |
| STAT2 | 4.51E-06 | 1.18949748 |
| TAGLN2 | 1.44E-06 | 1.18876514 |
| B4GALNT1 | 2.44E-02 | 1.18736014 |
| CLIC4 | 3.61E-05 | 1.18571612 |
| H3C14 | 4.04E-02 | 1.18317026 |
| MCM4 | 2.49E-03 | 1.18286976 |
| CFB | 1.56E-02 | 1.18275278 |
| XPR1 | 2.45E-08 | 1.18171273 |
| WAKMAR2 | 1.58E-02 | 1.18148945 |
| PSMA6 | 6.21E-05 | 1.18132736 |
| MARVELD3 | 1.56E-02 | 1.18085124 |
| APEX2 | 1.64E-04 | 1.18068509 |
| SNX32 | 4.43E-06 | 1.18034449 |
| TAP2 | 5.31E-04 | 1.17970535 |
| OPN3 | 1.70E-05 | 1.17963845 |
| F2R | 4.27E-03 | 1.17870496 |
| KLF7 | 9.04E-06 | 1.17835356 |
| FAM83H | 4.74E-02 | 1.17825232 |
| SFXN3 | 3.34E-08 | 1.17822717 |
| PON3 | 2.77E-02 | 1.1776075 |
| H3C15 | 4.36E-02 | 1.17720008 |
| TNK1 | 2.65E-04 | 1.17675524 |
| H2BC7 | 4.16E-02 | 1.17665338 |
| DIPK1A | 2.91E-06 | 1.17606207 |
| RNA18SN5 | 4.65E-02 | 1.17582571 |
| SLC7A5 | 3.61E-02 | 1.17368988 |
| DIAPH3-AS1 | 1.12E-02 | 1.17366607 |
| MARVELD1 | 7.27E-05 | 1.17351412 |
| OSBPL3 | 1.79E-04 | 1.17325401 |
| ITPR3-AS1 | 3.82E-03 | 1.17315423 |
| ZNF707 | 4.01E-12 | 1.17266711 |
| YKT6 | 5.78E-07 | 1.17260074 |
| ADM-DT | 6.83E-03 | 1.17238166 |
| ACTL6A | 2.87E-05 | 1.17183938 |
| TTC39B | 1.53E-03 | 1.17159222 |
| RHBDF2 | 1.09E-04 | 1.17118641 |
| TOX | 1.13E-02 | 1.17115367 |
| COTL1 | 2.51E-04 | 1.17052911 |
| CALD1 | 5.53E-03 | 1.17016959 |
| COL13A1 | 7.73E-03 | 1.16962727 |
| FBXL6 | 3.17E-07 | 1.16958006 |
| SPTSSA | 1.71E-04 | 1.16956401 |
| MIR3652 | 3.45E-05 | 1.16769193 |
| B3GAT3 | 2.32E-06 | 1.16760191 |
| IKBIP | 3.85E-04 | 1.16658626 |
| BUB1B | 4.32E-02 | 1.16657072 |
| FLNA | 1.89E-04 | 1.16575398 |
| C18orf54 | 1.01E-04 | 1.1655255 |
| PPARD | 4.83E-04 | 1.16501061 |
| MTBP | 6.51E-05 | 1.16465456 |
| LINC00857 | 1.92E-02 | 1.1643007 |
| PIP4K2C | 7.94E-06 | 1.16420443 |
| BISPR | 5.65E-03 | 1.16416216 |
| ELF4 | 3.87E-04 | 1.16326504 |
| PGF | 5.77E-04 | 1.16289114 |
| RGS17 | 1.40E-02 | 1.16239871 |
| PFN2 | 4.75E-03 | 1.16203868 |
| APBA2 | 1.76E-02 | 1.16200816 |
| TCF3 | 1.71E-06 | 1.16194037 |
| TFAP2A | 3.17E-02 | 1.16173005 |
| SEMA3C | 1.54E-03 | 1.16040685 |
| HIF1A-AS3 | 1.55E-02 | 1.16021278 |
| VANGL1 | 2.18E-04 | 1.15919748 |
| AMZ1 | 3.61E-03 | 1.15905852 |
| LRRC8C | 4.06E-04 | 1.15806366 |
| CMTM1 | 2.66E-07 | 1.15798207 |
| IGSF9 | 2.91E-02 | 1.15795489 |
| EVA1B | 1.46E-03 | 1.15704698 |
| SPAG5 | 6.83E-03 | 1.15631319 |
| DUSP4 | 4.63E-03 | 1.15530779 |
| GPC1 | 3.29E-03 | 1.15522174 |
| FUT1 | 3.41E-03 | 1.15513301 |
| POLE2 | 5.45E-03 | 1.15497035 |
| KIRREL1 | 2.38E-04 | 1.15457075 |
| DTL | 1.56E-02 | 1.15423279 |
| MTAP | 1.27E-05 | 1.15391897 |
| STIL | 1.50E-03 | 1.15331084 |
| CARD11 | 2.51E-02 | 1.15279936 |
| SCN8A | 3.80E-02 | 1.15167017 |
| PAPLN-AS1 | 3.09E-04 | 1.15133272 |
| DTD2 | 8.70E-06 | 1.15110088 |
| WNT10B | 2.52E-02 | 1.15099609 |
| C19orf48 | 1.89E-04 | 1.14875783 |
| CDK18 | 1.05E-05 | 1.14846861 |
| F2RL2 | 1.80E-02 | 1.14832477 |
| EHD1 | 5.26E-06 | 1.14828798 |
| CCND2-AS1 | 1.55E-02 | 1.14811764 |
| NRP2 | 2.56E-03 | 1.14727531 |
| DSCC1 | 6.33E-04 | 1.1470387 |
| ATP13A3 | 2.37E-08 | 1.14685973 |
| SMCO2 | 2.19E-02 | 1.14645195 |
| GSEC | 8.54E-03 | 1.14633744 |
| ZWINT | 1.24E-02 | 1.14632449 |
| DDX11-AS1 | 1.70E-03 | 1.14625553 |
| ZBED10P | 2.82E-03 | 1.14466778 |
| BICD2 | 6.85E-03 | 1.14446284 |
| H2BC8 | 1.89E-02 | 1.14338536 |
| KCNAB3 | 1.71E-03 | 1.14314887 |
| PYCR3 | 7.48E-06 | 1.14298726 |
| MYH10 | 6.40E-07 | 1.14274826 |
| NCLN | 1.22E-07 | 1.14217358 |
| LINC00638 | 2.62E-02 | 1.1418503 |
| EFNA1 | 1.26E-04 | 1.14026714 |
| NRSN2 | 1.41E-04 | 1.14009592 |
| BCAN-AS1 | 3.86E-02 | 1.13872057 |
| APCDD1 | 1.14E-03 | 1.13687116 |
| NTRK1 | 3.14E-02 | 1.13579127 |
| HILPDA-AS1 | 1.98E-02 | 1.13538443 |
| AKR1C3 | 4.14E-02 | 1.13506001 |
| CDT1 | 1.95E-02 | 1.13501016 |
| PLSCR3 | 5.16E-06 | 1.13497848 |
| DTX3L | 3.12E-05 | 1.13472614 |
| ATP10D | 2.22E-04 | 1.13346457 |
| ABL2 | 1.31E-05 | 1.13323217 |
| TFAP2A-AS1 | 3.13E-02 | 1.13295501 |
| EGFL6 | 4.22E-02 | 1.13244941 |
| TMTC3 | 1.81E-04 | 1.13238456 |
| GNAI1 | 2.62E-04 | 1.132279 |
| PIEZO1 | 1.63E-06 | 1.13193866 |
| APP | 3.95E-04 | 1.13155975 |
| TROAP | 3.77E-02 | 1.13145263 |
| B3GNT9 | 4.96E-05 | 1.13097023 |
| LILRB1 | 1.76E-02 | 1.13006807 |
| CCNB2 | 2.50E-02 | 1.12973774 |
| NPAS1 | 1.05E-03 | 1.12953484 |
| FURIN | 2.56E-05 | 1.12881757 |
| NCDN | 3.69E-06 | 1.12829987 |
| CDK1 | 3.67E-02 | 1.12802054 |
| FER1L4 | 2.33E-02 | 1.12734502 |
| MED10 | 4.66E-07 | 1.12667979 |
| POLA2 | 4.29E-06 | 1.12527577 |
| LIMA1 | 7.92E-06 | 1.1233704 |
| MAPK6 | 3.18E-04 | 1.12296894 |
| GZMB | 2.70E-02 | 1.11981524 |
| SNORA71A | 1.77E-02 | 1.11944667 |
| ZFP64 | 9.96E-06 | 1.11937424 |
| GPR137B | 1.36E-04 | 1.11911021 |
| YIF1A | 1.59E-05 | 1.11868633 |
| IQCG | 2.02E-05 | 1.1184908 |
| PSMB9 | 1.50E-03 | 1.118415 |
| IPO9-AS1 | 2.11E-08 | 1.11795552 |
| VDR | 9.32E-03 | 1.11765251 |
| MINPP1 | 4.19E-09 | 1.11614288 |
| MRGBP | 1.64E-06 | 1.11592075 |
| KTN1-AS1 | 6.33E-04 | 1.11574647 |
| TDRP | 1.92E-05 | 1.11522247 |
| RASSF10 | 2.51E-02 | 1.11514076 |
| DUSP10 | 1.51E-03 | 1.11458817 |
| CDYL2 | 9.54E-03 | 1.11450285 |
| BMAL2-AS1 | 3.73E-02 | 1.11372536 |
| DYRK3 | 3.33E-03 | 1.11343399 |
| CDR2L | 4.91E-05 | 1.11272943 |
| PON2 | 5.39E-07 | 1.11224186 |
| RDH11 | 2.64E-05 | 1.11090122 |
| TACC3 | 3.48E-03 | 1.10967957 |
| DPH3P1 | 1.39E-05 | 1.10929391 |
| HR | 2.17E-02 | 1.10911578 |
| FAM91A1 | 2.57E-06 | 1.10731429 |
| SGIP1 | 1.08E-02 | 1.10701239 |
| CD274 | 4.78E-03 | 1.10677582 |
| TIMELESS | 1.19E-03 | 1.10644995 |
| LMNB2 | 2.13E-03 | 1.10607621 |
| H2BC20P | 1.17E-03 | 1.10312246 |
| NLRC5 | 2.63E-03 | 1.10231659 |
| GINS2 | 8.53E-03 | 1.10212692 |
| GLI3 | 1.05E-05 | 1.10176187 |
| CKAP4 | 1.98E-04 | 1.09971469 |
| NUF2 | 3.10E-02 | 1.09932502 |
| RIN1 | 4.12E-03 | 1.09929175 |
| GM2A | 5.70E-03 | 1.09909854 |
| NPNT | 5.75E-03 | 1.0985533 |
| IFI27L2 | 7.13E-05 | 1.09737965 |
| CYP19A1 | 4.31E-02 | 1.09678033 |
| PNMA1 | 1.09E-04 | 1.09627092 |
| A1BG-AS1 | 3.48E-04 | 1.0932036 |
| MACF1 | 4.29E-10 | 1.09236466 |
| SLC26A10P | 4.45E-02 | 1.09233772 |
| TRMT61A | 5.05E-06 | 1.091789 |
| GPR132 | 1.10E-03 | 1.08962126 |
| MCM7 | 1.38E-03 | 1.08906577 |
| ADAM8 | 3.03E-03 | 1.08903341 |
| CAP1 | 1.93E-05 | 1.08898773 |
| FCGR2A | 1.95E-03 | 1.08888952 |
| ARHGAP11A | 1.60E-02 | 1.08791171 |
| LHFPL2 | 2.60E-04 | 1.08766695 |
| CHPF2 | 2.82E-07 | 1.08739524 |
| HERC6 | 4.61E-02 | 1.08736023 |
| ADAR | 1.38E-07 | 1.08709647 |
| SOX12 | 1.48E-04 | 1.08590105 |
| C2orf81 | 1.23E-02 | 1.08547109 |
| EIF2AK2 | 4.15E-10 | 1.08541953 |
| MIR9-3HG | 1.42E-02 | 1.08405836 |
| PELATON | 3.91E-03 | 1.08332771 |
| AFAP1-AS1 | 1.34E-02 | 1.08115197 |
| CALU | 4.77E-04 | 1.08095077 |
| TNFRSF4 | 6.44E-03 | 1.08061463 |
| PLD4 | 1.38E-02 | 1.08025669 |
| H4C9 | 2.46E-02 | 1.08021997 |
| DLG5-AS1 | 1.48E-02 | 1.08020284 |
| JPT2 | 8.25E-05 | 1.08017758 |
| DDIAS | 1.86E-02 | 1.07951529 |
| AP1S1 | 3.59E-05 | 1.07841392 |
| CRACR2A | 2.83E-02 | 1.07801227 |
| TRIM21 | 7.71E-05 | 1.0765821 |
| CPNE1 | 1.08E-03 | 1.07543969 |
| PAWR | 1.20E-04 | 1.0752267 |
| CBX8 | 6.62E-07 | 1.07489354 |
| ZDHHC12 | 4.18E-04 | 1.07477134 |
| CORIN | 4.74E-02 | 1.07337204 |
| CALHM5 | 2.95E-02 | 1.07324762 |
| SLC2A9-AS1 | 4.50E-03 | 1.07270742 |
| TRIM59 | 5.34E-03 | 1.07078904 |
| PIN4P1 | 1.27E-02 | 1.07062526 |
| BAX | 2.62E-05 | 1.07043593 |
| PIMREG | 1.38E-02 | 1.07032568 |
| C14orf119 | 3.45E-08 | 1.06997914 |
| ZNF200 | 1.72E-12 | 1.06902282 |
| NCAPG | 1.87E-02 | 1.06843116 |
| B4GALT4-AS1 | 2.88E-03 | 1.06781877 |
| BOLA2B | 3.48E-05 | 1.06743176 |
| CCDC137 | 1.81E-04 | 1.06733179 |
| LRP12 | 9.66E-04 | 1.06708828 |
| EHD2 | 2.55E-05 | 1.06685833 |
| TRAF7 | 7.74E-06 | 1.06600524 |
| CAD | 1.63E-04 | 1.06567553 |
| RNA5-8SN2 | 1.57E-03 | 1.06557255 |
| DYNLT2B | 8.02E-03 | 1.06531329 |
| PLCB3 | 9.37E-04 | 1.06406407 |
| UBE2S | 1.51E-03 | 1.06360254 |
| ITGB8-AS1 | 9.19E-03 | 1.06190539 |
| HHIPL2 | 2.94E-02 | 1.06185 |
| RNF152 | 5.74E-04 | 1.06154522 |
| PCDHB9 | 6.07E-03 | 1.06151518 |
| LARGE2 | 4.25E-02 | 1.06064957 |
| ADGRE2 | 1.93E-04 | 1.05994419 |
| PEDS1 | 6.84E-05 | 1.05971324 |
| SNRPB | 5.29E-05 | 1.05890902 |
| SCD5 | 9.35E-03 | 1.05836536 |
| TMEM106C | 8.34E-05 | 1.0582631 |
| SH3PXD2B | 4.05E-04 | 1.0578693 |
| AP5Z1 | 1.38E-07 | 1.05695428 |
| FOXP3 | 9.81E-04 | 1.05689864 |
| CD109-AS1 | 1.56E-04 | 1.05681167 |
| DNAJB11 | 5.59E-05 | 1.05678454 |
| CCL5 | 4.79E-02 | 1.05673624 |
| LRRC8A | 1.63E-03 | 1.05661551 |
| ANKRD29 | 3.72E-03 | 1.05602546 |
| FAM3C | 2.10E-05 | 1.05557521 |
| CLDN18 | 8.95E-03 | 1.05408913 |
| IFI35 | 2.70E-04 | 1.05351266 |
| PRKDC | 1.14E-04 | 1.05303567 |
| YDJC | 1.90E-03 | 1.05296849 |
| EBNA1BP2 | 1.85E-04 | 1.05255618 |
| SAC3D1 | 6.51E-04 | 1.05245573 |
| DHX58 | 1.48E-04 | 1.05222337 |
| GSTP1 | 1.66E-02 | 1.0518396 |
| H2AC7 | 4.02E-02 | 1.05143967 |
| SPAG1 | 1.32E-03 | 1.05074895 |
| POLR2G | 3.67E-06 | 1.04958835 |
| LINC01232 | 9.95E-06 | 1.04924372 |
| TLCD3A | 4.26E-03 | 1.04906791 |
| NFKBIE | 3.91E-03 | 1.04903097 |
| FBXL18 | 7.12E-05 | 1.04875384 |
| SLC44A1 | 1.06E-05 | 1.04865831 |
| C11orf24 | 2.58E-03 | 1.04807406 |
| FMNL3 | 4.00E-07 | 1.04636618 |
| HSF2BP | 1.11E-02 | 1.04616504 |
| PCK2 | 6.55E-04 | 1.04611481 |
| CELSR1 | 1.43E-02 | 1.04508218 |
| CYTOR | 1.54E-02 | 1.04490219 |
| CCDC103 | 4.08E-03 | 1.04312802 |
| NOP14-AS1 | 9.99E-05 | 1.04003341 |
| SSC4D | 6.81E-03 | 1.03961628 |
| YWHAZ | 9.35E-03 | 1.03948924 |
| SNORA79B | 2.01E-03 | 1.03918848 |
| DOLPP1 | 2.92E-04 | 1.0388771 |
| NUP62CL | 2.50E-03 | 1.03884522 |
| ATOSB | 2.83E-03 | 1.03861448 |
| LINC00839 | 4.55E-02 | 1.03859995 |
| LRRC59 | 1.05E-04 | 1.03856212 |
| ZC3H12A-DT | 2.44E-03 | 1.03846605 |
| TNFAIP1 | 2.11E-05 | 1.03830151 |
| H3C1 | 2.50E-02 | 1.03800901 |
| TSPAN5 | 2.51E-04 | 1.03580616 |
| TUBA4A | 1.36E-02 | 1.035542 |
| REEP3 | 3.94E-06 | 1.03548077 |
| PSMB8 | 6.70E-04 | 1.03544615 |
| CCNE2 | 7.87E-03 | 1.03531792 |
| RNA5-8SN5 | 2.00E-03 | 1.03523489 |
| RHEBL1 | 1.49E-03 | 1.03482033 |
| ANKLE2 | 3.81E-06 | 1.03476955 |
| GASAL1 | 4.13E-02 | 1.03388373 |
| KIF20A | 4.02E-02 | 1.03380163 |
| N4BP1 | 1.25E-04 | 1.03286692 |
| RAD51 | 1.03E-02 | 1.03262989 |
| CHRNB2 | 4.63E-07 | 1.03171161 |
| CCT5 | 2.16E-04 | 1.03035004 |
| CCNB1 | 3.06E-02 | 1.0299811 |
| TMEM229B | 3.62E-02 | 1.0296358 |
| C20orf27 | 1.67E-05 | 1.02949852 |
| GANAB | 3.14E-06 | 1.02919644 |
| ITPA | 1.53E-06 | 1.02907433 |
| SLC22A23 | 5.04E-03 | 1.02849896 |
| KCTD11 | 1.24E-02 | 1.02812106 |
| NUDT1 | 6.35E-04 | 1.02778696 |
| FLNB | 3.37E-06 | 1.0271466 |
| CH507-42P11.6 | 1.85E-02 | 1.02677376 |
| SPON2 | 6.36E-03 | 1.02450998 |
| FAM86C1P | 7.43E-04 | 1.02442734 |
| SDSL | 2.17E-02 | 1.0243429 |
| CLBA1 | 2.08E-05 | 1.02290469 |
| FTL | 2.04E-03 | 1.02268252 |
| CZ1P-ASNS | 4.67E-03 | 1.02142486 |
| NKILA | 2.50E-02 | 1.02041553 |
| IFNGR1 | 3.24E-04 | 1.0201847 |
| TUBB2A | 2.53E-02 | 1.01962014 |
| PIP5KL1 | 2.99E-02 | 1.01924152 |
| LRWD1 | 2.15E-05 | 1.01920572 |
| AGBL2 | 6.35E-04 | 1.01889956 |
| CCDC34 | 5.01E-04 | 1.01887992 |
| ZNF697 | 1.85E-03 | 1.01885203 |
| BOP1 | 3.28E-04 | 1.01782938 |
| LYN | 7.42E-03 | 1.01682656 |
| ACLY | 1.58E-06 | 1.01666894 |
| SPTLC3 | 8.06E-03 | 1.01644706 |
| PATL1 | 6.18E-06 | 1.01632072 |
| CSPG4 | 4.51E-02 | 1.01605702 |
| YEATS2-AS1 | 5.73E-08 | 1.01600695 |
| SLC29A3 | 6.47E-03 | 1.015326 |
| EME1 | 1.31E-04 | 1.0149819 |
| PTGES | 3.82E-02 | 1.01491478 |
| CMIP | 2.52E-04 | 1.01476279 |
| CLCN7 | 1.04E-08 | 1.01442293 |
| CLDN22 | 4.09E-02 | 1.01407242 |
| LAMB1 | 1.13E-03 | 1.01386059 |
| PSMG3 | 1.20E-05 | 1.01347689 |
| RAB42 | 1.22E-02 | 1.01346799 |
| CHTF18 | 2.67E-03 | 1.01310737 |
| TLR6 | 3.32E-04 | 1.01177291 |
| KLHL6 | 1.35E-02 | 1.01132489 |
| PPT1 | 6.10E-07 | 1.01084539 |
| TSPAN15 | 2.45E-03 | 1.01040861 |
| MAD2L1 | 2.08E-02 | 1.00961061 |
| NRBF2 | 3.10E-04 | 1.00946723 |
| TMX1 | 1.23E-05 | 1.00894326 |
| CHST7 | 8.78E-03 | 1.0083783 |
| GCSAM | 2.93E-02 | 1.00810328 |
| SKP2 | 4.56E-03 | 1.00766758 |
| DZIP1L | 5.34E-03 | 1.00748633 |
| MEN1 | 9.95E-06 | 1.00744005 |
| ZNHIT2 | 8.95E-04 | 1.00678773 |
| TMEM63B | 3.29E-04 | 1.00595011 |
| TMEFF1 | 1.41E-02 | 1.00566776 |
| UNC119 | 2.00E-04 | 1.00515498 |
| MAFB | 3.50E-03 | 1.00474727 |
| GPR161 | 6.92E-04 | 1.00445347 |
| NCAPG2 | 1.79E-03 | 1.00422783 |
| MYH9 | 1.45E-05 | 1.00409477 |
| FXYD5 | 2.29E-02 | 1.00360416 |
| ADCK5 | 8.54E-06 | 1.00335675 |
| RFC4 | 1.54E-03 | 1.00327186 |
| CTPS1 | 1.08E-04 | 1.00317124 |
| IFITM3 | 1.69E-04 | 1.00292606 |
| ATRN | 1.66E-05 | 1.00286324 |
| FLVCR2 | 1.56E-02 | 1.00258668 |
| POC1A | 1.31E-03 | 1.00121356 |
| SKAP2 | 1.30E-03 | 1.00118764 |
| RUVBL1 | 5.25E-05 | 1.00104101 |
| SLC29A4 | 2.11E-02 | 1.00086191 |
| DDIT4 | 3.40E-02 | 1.0001839 |
| ACE | 1.09E-02 | -1.00041083 |
| CRIP1 | 1.27E-02 | -1.00111529 |
| PIK3R1 | 8.81E-03 | -1.00165019 |
| IRAG1 | 1.27E-02 | -1.00178314 |
| ELOVL6 | 2.26E-02 | -1.00201919 |
| ACOT1 | 4.12E-03 | -1.00320251 |
| MIR6511B1 | 1.67E-03 | -1.00471096 |
| PCAT19 | 1.94E-02 | -1.00473997 |
| MIR5006 | 2.14E-04 | -1.00681067 |
| GNA14 | 3.17E-02 | -1.00705185 |
| TRIM45 | 2.13E-03 | -1.0073105 |
| SYN1 | 8.13E-03 | -1.00783243 |
| TMX4 | 5.25E-03 | -1.00818172 |
| ADCY9 | 7.21E-04 | -1.01075025 |
| ARHGEF10L | 3.68E-04 | -1.01098205 |
| L3MBTL1 | 6.39E-03 | -1.01123825 |
| UQCRC2 | 1.91E-05 | -1.01136625 |
| H3P4 | 1.15E-02 | -1.01189515 |
| ZSCAN16-AS1 | 4.73E-03 | -1.01240679 |
| ARHGEF26 | 3.52E-02 | -1.01424924 |
| ZNF257 | 3.71E-02 | -1.01436419 |
| ZNF253 | 1.41E-02 | -1.01461251 |
| TRNG | 7.35E-03 | -1.01489675 |
| FLT3 | 2.65E-02 | -1.01547679 |
| TAFA2 | 3.19E-02 | -1.01575359 |
| IQCK | 1.15E-03 | -1.0163203 |
| MAP3K8 | 3.37E-02 | -1.01692261 |
| FAHD2B | 2.52E-02 | -1.0176139 |
| CEP112 | 2.43E-02 | -1.01820399 |
| EPHX2 | 3.90E-03 | -1.01984216 |
| P4HTM | 8.74E-03 | -1.02010741 |
| SSPN | 3.12E-02 | -1.02014999 |
| PBXIP1 | 4.67E-03 | -1.02030914 |
| KIF1C-AS1 | 7.10E-04 | -1.02037004 |
| XK | 1.61E-02 | -1.02085832 |
| SNHG14 | 2.57E-02 | -1.0212363 |
| TET2-AS1 | 1.13E-02 | -1.0220246 |
| GRK6P1 | 3.82E-02 | -1.02357611 |
| SNORD50B | 1.29E-03 | -1.02358747 |
| CNRIP1 | 4.74E-02 | -1.02363202 |
| TRABD2B | 2.91E-02 | -1.02392293 |
| NOXA1 | 2.91E-02 | -1.0248385 |
| DICER1-AS1 | 1.00E-02 | -1.02520622 |
| CLMN | 1.57E-02 | -1.02529602 |
| CATSPERG | 1.53E-02 | -1.02752064 |
| KIF1C | 1.13E-03 | -1.02756273 |
| RPP14 | 2.12E-05 | -1.02783189 |
| PROX1 | 2.96E-02 | -1.02939629 |
| DPEP2 | 3.79E-02 | -1.02950229 |
| ZC3H6 | 4.10E-04 | -1.02985727 |
| ABCB4 | 1.83E-05 | -1.03035244 |
| EFCAB2 | 5.57E-03 | -1.03052092 |
| CD55 | 1.65E-02 | -1.03091821 |
| SLC3A1 | 3.79E-04 | -1.03123948 |
| CCNI2 | 1.05E-02 | -1.03234343 |
| TSPYL6 | 3.25E-02 | -1.03276771 |
| AFF1-AS1 | 1.19E-03 | -1.03309699 |
| NEURL2 | 2.10E-02 | -1.03319485 |
| SHMT1 | 3.83E-04 | -1.03522764 |
| UQCRC1 | 2.12E-04 | -1.03561892 |
| TACC1 | 1.16E-03 | -1.03636196 |
| LINC00663 | 1.75E-02 | -1.03645396 |
| ATP2A2 | 7.36E-04 | -1.03773477 |
| KIAA0232 | 8.42E-08 | -1.03835778 |
| TAL1 | 3.19E-02 | -1.03870338 |
| MAN2A2 | 1.88E-04 | -1.03924473 |
| LINC03011 | 1.80E-02 | -1.04019977 |
| KHK | 1.27E-02 | -1.04126578 |
| VWCE | 3.24E-02 | -1.0415934 |
| UBAC1 | 1.00E-03 | -1.0416484 |
| ZNF519 | 1.85E-03 | -1.04173509 |
| ZNF385C | 1.66E-02 | -1.04201304 |
| TNRC6B-DT | 4.72E-02 | -1.04211071 |
| ATP5F1A | 7.24E-05 | -1.04216027 |
| ATP8 | 1.52E-02 | -1.04279229 |
| LINC01963 | 9.89E-04 | -1.04283044 |
| MSRA | 1.38E-04 | -1.04317817 |
| PID1 | 3.44E-02 | -1.04339439 |
| SPATA18 | 1.20E-02 | -1.04471666 |
| MGST3 | 1.02E-03 | -1.04477223 |
| PRADC1 | 9.70E-04 | -1.04511751 |
| PHPT1 | 6.09E-04 | -1.04557783 |
| CELF2 | 1.34E-02 | -1.04565328 |
| IRF1-AS1 | 2.58E-02 | -1.04602121 |
| CD209 | 3.93E-02 | -1.04607641 |
| PRIMA1 | 4.71E-02 | -1.0468369 |
| ZNF382 | 1.64E-02 | -1.04685186 |
| HSPB2-C11orf52 | 2.11E-02 | -1.04715321 |
| ANKRD37 | 1.68E-03 | -1.04936364 |
| MIR27B | 2.54E-02 | -1.04945903 |
| MIR100HG | 3.37E-02 | -1.04951935 |
| MAP4 | 2.03E-03 | -1.05374243 |
| FAM85B | 2.66E-02 | -1.05386838 |
| MCF2L | 2.54E-02 | -1.05406257 |
| ADAMTSL4 | 3.67E-03 | -1.05446456 |
| SPRY1 | 3.06E-02 | -1.0554267 |
| HDAC4 | 3.08E-04 | -1.0560838 |
| PDZD9 | 4.77E-06 | -1.05658566 |
| PCSK4 | 4.16E-03 | -1.05754303 |
| SNRPN | 1.37E-02 | -1.05766368 |
| CCT6B | 8.51E-04 | -1.06152818 |
| SLC16A14 | 8.49E-03 | -1.06176411 |
| ST3GAL3 | 6.42E-03 | -1.06213877 |
| EDA2R | 2.03E-02 | -1.06233769 |
| UBAP1L | 5.51E-04 | -1.0623746 |
| COX5B | 6.94E-05 | -1.06243428 |
| ARHGAP44 | 1.24E-02 | -1.06261935 |
| EEPD1 | 1.81E-03 | -1.06337684 |
| MGLL | 5.03E-03 | -1.06363068 |
| LINC02615 | 7.02E-03 | -1.06465626 |
| PHYHD1 | 3.51E-02 | -1.06472019 |
| KCNMB1 | 8.90E-03 | -1.06551512 |
| ZNF25 | 6.21E-03 | -1.06646411 |
| MGST2 | 6.87E-05 | -1.06666114 |
| BTBD19 | 4.21E-02 | -1.06676109 |
| MYCT1 | 4.26E-02 | -1.0671024 |
| MIR29B2CHG | 3.36E-02 | -1.06718773 |
| ZNF578 | 3.44E-02 | -1.06736535 |
| C10orf105 | 3.79E-02 | -1.06742487 |
| PSMG3-AS1 | 9.08E-03 | -1.06757548 |
| ZNF366 | 9.61E-03 | -1.06850774 |
| LPAR1 | 2.11E-02 | -1.06866397 |
| LONRF1 | 3.57E-03 | -1.0686942 |
| CD200R1 | 2.06E-02 | -1.07037608 |
| MLANA | 3.02E-03 | -1.07044469 |
| EPB41L1-AS1 | 4.36E-02 | -1.07111057 |
| SAR1B | 4.04E-05 | -1.07113755 |
| MRNIP-DT | 1.88E-02 | -1.07138765 |
| PRKAR2A-AS1 | 3.48E-03 | -1.07250234 |
| ZNF181 | 2.74E-03 | -1.07294983 |
| SERPINI1 | 2.54E-03 | -1.07310481 |
| CEBPA-DT | 1.06E-02 | -1.07544635 |
| TSTD2 | 9.59E-04 | -1.07694445 |
| VSIG10 | 5.85E-05 | -1.07706867 |
| MCRIP2 | 6.81E-03 | -1.07922162 |
| ATP5PO | 1.91E-06 | -1.07933865 |
| TBC1D4 | 2.75E-05 | -1.07951124 |
| ZNF662 | 3.46E-02 | -1.08243463 |
| ALDH2 | 8.96E-05 | -1.08383148 |
| GPR3 | 3.03E-02 | -1.08420367 |
| ITPRIP-AS1 | 4.07E-02 | -1.08455726 |
| KCNIP3 | 3.93E-02 | -1.08648819 |
| BRSK2 | 1.02E-02 | -1.08758321 |
| FUNDC2 | 8.74E-04 | -1.08781212 |
| TRNL1 | 7.04E-03 | -1.08851617 |
| RUSC2 | 6.05E-03 | -1.08889212 |
| FBXL17 | 1.55E-06 | -1.08924476 |
| ZNF358 | 6.76E-03 | -1.08928963 |
| PLLP | 2.17E-02 | -1.08998237 |
| C8orf88 | 2.04E-02 | -1.0901309 |
| MAPKAPK3 | 1.78E-04 | -1.09023183 |
| NDUFA5 | 1.03E-05 | -1.09084718 |
| GYS1 | 1.76E-03 | -1.09160996 |
| TIAF1 | 3.21E-04 | -1.09168596 |
| RPUSD4 | 2.88E-04 | -1.09321387 |
| CLIP1-AS1 | 2.16E-02 | -1.09412409 |
| KANK3 | 1.72E-03 | -1.09433938 |
| TBX2-AS1 | 2.90E-02 | -1.09459016 |
| KCTD6 | 4.62E-07 | -1.09484514 |
| PPIL6 | 4.18E-07 | -1.09585904 |
| NODAL | 2.08E-03 | -1.09662643 |
| PECAM1 | 1.86E-02 | -1.0966891 |
| IL3RA | 1.93E-03 | -1.09713398 |
| MGAT3 | 1.95E-02 | -1.09801652 |
| PDE8B | 2.29E-02 | -1.09969208 |
| VAMP5 | 2.30E-03 | -1.09994679 |
| GNG11 | 6.28E-03 | -1.1015754 |
| ZNF436-AS1 | 3.22E-03 | -1.10252276 |
| CHRNA7 | 4.12E-03 | -1.10259883 |
| LRRK2 | 7.54E-03 | -1.10372355 |
| SNORD50A | 4.52E-03 | -1.10472584 |
| UNC79 | 1.86E-04 | -1.10579701 |
| SUCLG2 | 4.79E-08 | -1.10583355 |
| RMND5B | 1.83E-05 | -1.10617911 |
| KIT | 4.61E-02 | -1.10696622 |
| SNURF | 1.08E-02 | -1.10704534 |
| GOLGA6L9 | 1.05E-02 | -1.1089591 |
| PLIN2 | 3.99E-02 | -1.10951823 |
| PPM1J | 5.32E-03 | -1.10957797 |
| UCN | 4.38E-02 | -1.10964629 |
| TFPI | 3.09E-02 | -1.1097239 |
| DLGAP1 | 4.13E-02 | -1.10978923 |
| CIPC | 7.34E-05 | -1.11007407 |
| RALGPS1 | 1.57E-04 | -1.11154975 |
| TNFRSF11A | 1.41E-02 | -1.11264706 |
| CYBRD1 | 1.59E-02 | -1.11289571 |
| C19orf47 | 1.49E-04 | -1.11501743 |
| TEK | 3.40E-02 | -1.11521535 |
| PRICKLE4 | 1.35E-04 | -1.11545136 |
| RNU5B-1 | 4.01E-02 | -1.11589293 |
| SYDE2 | 8.33E-03 | -1.11643086 |
| ZNF470 | 2.46E-02 | -1.11711617 |
| DYNLT4 | 4.34E-02 | -1.11776909 |
| SAMD14 | 5.42E-03 | -1.11781391 |
| FBLN1 | 2.11E-02 | -1.11988059 |
| C22orf39 | 1.14E-04 | -1.12044987 |
| LINC01786 | 2.20E-03 | -1.1211717 |
| DUSP28 | 1.74E-05 | -1.12207186 |
| GIMAP1-GIMAP5 | 7.93E-03 | -1.12220005 |
| ATPAF1 | 6.66E-05 | -1.12266085 |
| MID1IP1 | 1.63E-03 | -1.12318744 |
| MIR4257 | 1.81E-02 | -1.12331137 |
| MACC1 | 4.65E-02 | -1.12442611 |
| RGS16 | 4.88E-02 | -1.12564904 |
| FGL2 | 6.69E-03 | -1.12661402 |
| GIMAP5 | 8.53E-03 | -1.12765359 |
| ZFP28 | 4.73E-02 | -1.12777979 |
| UBR3 | 2.06E-05 | -1.12797206 |
| DIAPH1-AS1 | 1.10E-02 | -1.12953751 |
| DUSP1 | 8.81E-03 | -1.13213324 |
| ISOC1 | 1.23E-08 | -1.1326474 |
| GUCY2C | 6.13E-03 | -1.13416927 |
| MTFR1L | 2.81E-05 | -1.13583059 |
| LYRM7 | 5.59E-06 | -1.13588259 |
| SYCP3 | 5.94E-03 | -1.13638111 |
| ETFDH | 2.63E-06 | -1.13653504 |
| LRRFIP2 | 1.36E-07 | -1.13729224 |
| RAMP3 | 2.21E-02 | -1.1377583 |
| ABLIM3 | 3.80E-03 | -1.13789912 |
| ZNF429 | 3.53E-03 | -1.13952397 |
| LINC01504 | 3.79E-02 | -1.13975899 |
| POU6F1 | 3.70E-03 | -1.14052749 |
| LINC02256 | 3.86E-03 | -1.1408469 |
| FGD5 | 5.75E-03 | -1.14185783 |
| SASH1 | 4.87E-04 | -1.14316587 |
| PARVG | 1.86E-03 | -1.14367768 |
| PM20D2 | 3.58E-05 | -1.14376077 |
| GIMAP6 | 5.62E-03 | -1.14407454 |
| RNF125 | 1.21E-02 | -1.14438194 |
| METTL27 | 3.65E-02 | -1.14696055 |
| TBX3 | 1.12E-02 | -1.14708468 |
| SPATA6 | 3.54E-02 | -1.14747796 |
| PDK2 | 3.28E-03 | -1.14810282 |
| LINC01589 | 2.24E-02 | -1.14821388 |
| NOVA2 | 1.09E-02 | -1.14979402 |
| CRY2 | 1.04E-04 | -1.15163072 |
| EPB41L3 | 4.18E-03 | -1.15526795 |
| DENND2A | 1.92E-02 | -1.15578095 |
| MDH1 | 6.87E-05 | -1.1565757 |
| SPRY2 | 9.93E-04 | -1.1569081 |
| SULT1C4 | 4.64E-02 | -1.15815499 |
| SMDT1 | 1.04E-08 | -1.15821711 |
| SEMA6A-AS1 | 6.91E-03 | -1.15825704 |
| PDHB | 1.50E-06 | -1.15837794 |
| NDUFC1 | 1.37E-05 | -1.1590173 |
| TSPOAP1 | 1.92E-02 | -1.15903669 |
| SYT15 | 5.93E-03 | -1.15962075 |
| SATB1 | 1.02E-03 | -1.15964682 |
| ZNF486 | 1.34E-02 | -1.16083547 |
| TMEM38B | 2.66E-03 | -1.16095678 |
| CMA1 | 9.95E-03 | -1.1640643 |
| GHR | 6.32E-03 | -1.16407493 |
| KBTBD11 | 1.25E-02 | -1.16410699 |
| RNVU1-18 | 3.67E-02 | -1.16412112 |
| SSBP2 | 6.17E-03 | -1.16710795 |
| KLHDC1 | 2.56E-02 | -1.16756536 |
| SHROOM3 | 4.27E-02 | -1.1684614 |
| DNAJB5 | 9.72E-03 | -1.16871199 |
| ZNF91 | 1.17E-02 | -1.16872129 |
| HYMAI | 1.89E-02 | -1.16944152 |
| GIMAP8 | 5.72E-03 | -1.16961696 |
| SNORD118 | 1.61E-03 | -1.17047164 |
| PNPLA7 | 4.30E-02 | -1.17181218 |
| DDAH1 | 2.07E-02 | -1.17319576 |
| TMEM220 | 4.80E-04 | -1.17365623 |
| ZNF583 | 1.89E-02 | -1.17416242 |
| LINC02175 | 6.32E-03 | -1.1750925 |
| HSDL2 | 1.38E-06 | -1.17517558 |
| FHIT | 1.12E-02 | -1.17546375 |
| ANGPTL7 | 1.39E-02 | -1.17651588 |
| MAP3K20-AS1 | 2.39E-03 | -1.176932 |
| SLC5A4 | 3.32E-04 | -1.17727871 |
| PKIG | 7.86E-03 | -1.17746864 |
| TRNI | 5.88E-03 | -1.17750352 |
| BCL2 | 2.52E-03 | -1.17782594 |
| GIMAP7 | 8.53E-03 | -1.17807697 |
| BST1 | 1.92E-02 | -1.17813247 |
| METTL7A | 1.79E-02 | -1.17816107 |
| SYT15B | 1.67E-03 | -1.17850212 |
| COX7C | 9.13E-08 | -1.18057045 |
| KRBA2 | 6.69E-04 | -1.18101766 |
| MTARC2 | 1.77E-02 | -1.18149638 |
| EDRF1-DT | 1.58E-03 | -1.18213271 |
| ZNF208 | 4.56E-02 | -1.1831588 |
| FAM86B2-DT | 9.68E-03 | -1.18334012 |
| GOT1-DT | 3.99E-03 | -1.18445703 |
| IFFO1 | 6.80E-03 | -1.18483893 |
| PWAR6 | 4.12E-02 | -1.18586709 |
| LTC4S | 4.60E-03 | -1.18959926 |
| SYT2 | 2.81E-03 | -1.18996512 |
| ZNF717 | 1.69E-02 | -1.19011431 |
| CEP85L | 4.95E-03 | -1.19079948 |
| TRIL | 2.95E-02 | -1.19156294 |
| PCDHB4 | 3.35E-02 | -1.19192845 |
| UBE2D4 | 3.69E-06 | -1.19206196 |
| TLL2 | 3.53E-02 | -1.19213053 |
| UBE2Q2P2 | 8.27E-03 | -1.19388586 |
| IDI2-AS1 | 3.43E-05 | -1.19447318 |
| PLAGL1 | 5.05E-03 | -1.19533691 |
| CAPN5 | 6.48E-03 | -1.19595587 |
| GRAMD1B | 6.89E-03 | -1.19655469 |
| HSPA12B | 5.37E-03 | -1.19913891 |
| EIF1AY | 3.22E-02 | -1.19960686 |
| TRNS2 | 3.51E-03 | -1.19974464 |
| ZNF568 | 1.41E-02 | -1.19983482 |
| EPB41L4A-DT | 3.34E-02 | -1.20041984 |
| AKAP1 | 1.26E-05 | -1.20168889 |
| ANXA11 | 3.89E-09 | -1.20282608 |
| CRLF1 | 1.06E-02 | -1.2032595 |
| TNS1-AS1 | 4.25E-03 | -1.20365229 |
| LINC02019 | 1.27E-03 | -1.203859 |
| GFRA2 | 1.24E-02 | -1.20432863 |
| MAML3 | 3.92E-05 | -1.20506016 |
| SOCS3 | 3.83E-02 | -1.20513741 |
| SIRT2 | 4.98E-04 | -1.20581908 |
| SLCO4A1-AS2 | 1.02E-02 | -1.20627341 |
| ABCD2 | 7.92E-03 | -1.20684922 |
| CRACDL | 1.87E-03 | -1.20887896 |
| TMEM273 | 4.40E-03 | -1.20923019 |
| ZFP3 | 1.74E-03 | -1.20971313 |
| NR2E3 | 2.11E-02 | -1.21026308 |
| RNU4ATAC | 1.32E-02 | -1.21057322 |
| TRIP10 | 2.32E-06 | -1.21084617 |
| RNF123 | 1.15E-04 | -1.21089168 |
| PRLR | 4.32E-02 | -1.21176793 |
| NNT-AS1 | 6.30E-03 | -1.21233783 |
| LRIG1 | 9.59E-03 | -1.21290951 |
| MEIS1 | 1.22E-03 | -1.21420176 |
| ECHDC2 | 1.79E-03 | -1.21421418 |
| DCAF6 | 3.63E-04 | -1.21524224 |
| PRR7-AS1 | 2.47E-03 | -1.2169288 |
| C22orf15 | 5.74E-03 | -1.21716285 |
| CTSF | 2.58E-02 | -1.21721612 |
| S1PR1 | 1.38E-02 | -1.2172256 |
| CTSG | 1.60E-02 | -1.21775705 |
| MIR4273 | 1.88E-02 | -1.21821664 |
| ADCY1 | 3.66E-02 | -1.21849102 |
| MYO18A | 1.12E-04 | -1.21869751 |
| ALDH6A1 | 1.13E-03 | -1.21968095 |
| PRKG2 | 1.26E-02 | -1.2199571 |
| CFAP100 | 1.60E-03 | -1.22014305 |
| SLC10A5 | 1.10E-02 | -1.22050111 |
| SAMD13 | 2.87E-02 | -1.22126488 |
| C1orf21 | 4.34E-07 | -1.22291763 |
| FSD1L | 6.58E-03 | -1.22529798 |
| PLEKHA7 | 3.26E-03 | -1.225773 |
| FNDC4 | 4.89E-03 | -1.22896178 |
| MIR637 | 1.35E-02 | -1.22919032 |
| TNNI3K | 4.59E-02 | -1.23026648 |
| ITGAD | 1.68E-02 | -1.23044481 |
| KCNIP2-AS1 | 1.49E-03 | -1.2307583 |
| ZYG11B | 9.49E-06 | -1.23172584 |
| MAP3K14 | 1.57E-07 | -1.23216521 |
| THRB | 3.38E-03 | -1.23257376 |
| FBXO32 | 2.70E-02 | -1.23337264 |
| HAAO | 9.71E-04 | -1.23390815 |
| ECSCR | 1.51E-02 | -1.2341458 |
| TRNQ | 7.68E-03 | -1.23629816 |
| SERPINB9P1 | 1.77E-02 | -1.23905426 |
| MAP10 | 8.60E-03 | -1.23942877 |
| PRKG1 | 2.35E-02 | -1.23963681 |
| PROB1 | 3.35E-02 | -1.23988011 |
| GPRC5C | 1.91E-02 | -1.24042976 |
| SAMD4A | 8.24E-03 | -1.24109257 |
| NUP210L | 5.71E-04 | -1.24464582 |
| AP1S2 | 3.78E-03 | -1.24507074 |
| PBX1 | 1.98E-03 | -1.24593331 |
| OLFML1 | 2.02E-02 | -1.24614278 |
| NDUFS7 | 1.15E-04 | -1.24654558 |
| CMAHP | 2.45E-03 | -1.24664034 |
| S100A4 | 6.48E-05 | -1.24811502 |
| MYO15B | 2.72E-04 | -1.24895496 |
| FUT5 | 4.61E-02 | -1.2522403 |
| NFIC | 2.68E-06 | -1.25289732 |
| ECH1 | 4.29E-05 | -1.25328639 |
| PRX | 6.51E-03 | -1.25364232 |
| ASB16-AS1 | 4.98E-04 | -1.25365768 |
| FGFR4 | 1.93E-02 | -1.25439995 |
| PDE9A | 3.38E-03 | -1.25500057 |
| SLC45A1 | 1.59E-02 | -1.25956597 |
| GBE1 | 2.26E-05 | -1.26061259 |
| CHRNG | 1.79E-02 | -1.26107051 |
| EPIST | 4.62E-02 | -1.26271879 |
| CASP4LP | 1.20E-02 | -1.2637285 |
| SRRM2-AS1 | 4.46E-04 | -1.2637694 |
| DNASE1L3 | 2.71E-02 | -1.26420103 |
| MSS51 | 2.94E-03 | -1.26422852 |
| GATD3 | 1.88E-06 | -1.26442183 |
| SLC1A1 | 1.24E-02 | -1.26500636 |
| PLCD4 | 3.33E-03 | -1.2655694 |
| HBEGF | 2.47E-02 | -1.26895901 |
| LINC00957 | 1.11E-04 | -1.27017388 |
| MTURN | 5.88E-03 | -1.2710534 |
| GALNT12 | 3.85E-02 | -1.27110835 |
| RNF115 | 1.46E-05 | -1.27123409 |
| SLC16A12 | 4.58E-02 | -1.271982 |
| NFIA | 1.06E-05 | -1.27217743 |
| C3orf49 | 4.39E-05 | -1.27357942 |
| LURAP1 | 1.09E-03 | -1.2743639 |
| ADM5 | 1.13E-03 | -1.27480632 |
| BVES | 1.95E-02 | -1.27561067 |
| ACSM6 | 3.90E-03 | -1.27584869 |
| RASA4DP | 1.52E-03 | -1.27628678 |
| CD79B | 7.26E-03 | -1.27709773 |
| RNVU1-6 | 1.45E-02 | -1.27829583 |
| ZNF418 | 1.40E-02 | -1.27846034 |
| SESN1 | 1.85E-04 | -1.27856986 |
| C15orf61 | 1.06E-03 | -1.28113671 |
| NOSTRIN | 1.04E-02 | -1.2816286 |
| ARHGEF6 | 3.58E-03 | -1.28224337 |
| KLF9 | 6.30E-03 | -1.28251822 |
| PHYHIPL | 4.88E-02 | -1.28450227 |
| ZNF300P1 | 2.74E-02 | -1.2884555 |
| CFP | 3.29E-04 | -1.28866732 |
| ERVH48-1 | 4.35E-03 | -1.29027207 |
| ITIH5 | 1.91E-02 | -1.2910245 |
| REEP1 | 1.89E-02 | -1.29156648 |
| RORB | 2.45E-02 | -1.29224768 |
| MYB | 3.30E-03 | -1.29229982 |
| IGSF9B | 2.92E-02 | -1.29280356 |
| CCL21 | 4.42E-02 | -1.29334376 |
| LDB2 | 4.83E-03 | -1.2960576 |
| PPM1E | 9.00E-04 | -1.29644236 |
| TACR2 | 1.56E-04 | -1.2979008 |
| TRIM52-AS1 | 2.19E-03 | -1.29792465 |
| ZFP36 | 3.40E-03 | -1.29819544 |
| EFCC1 | 1.08E-03 | -1.30197601 |
| CPEB3 | 1.57E-05 | -1.30320959 |
| GYG1 | 2.38E-03 | -1.30365533 |
| DIPK2B | 4.55E-03 | -1.30368544 |
| AVPR2 | 7.89E-03 | -1.30431522 |
| CISH | 3.20E-05 | -1.30446656 |
| CFAP126 | 4.07E-05 | -1.30464289 |
| BSN | 8.84E-03 | -1.30569076 |
| CCDC9B | 9.35E-04 | -1.30648824 |
| CLEC14A | 2.99E-03 | -1.30674667 |
| CDON | 4.74E-04 | -1.30888397 |
| CLEC10A | 1.11E-02 | -1.30891115 |
| TACC2 | 1.19E-04 | -1.30985704 |
| THSD4 | 1.13E-03 | -1.31008452 |
| MVB12B | 1.09E-04 | -1.31024582 |
| SP2-DT | 1.21E-03 | -1.3103813 |
| SLC6A17 | 3.23E-02 | -1.31185618 |
| MPDZ | 2.14E-02 | -1.31204357 |
| COLEC12 | 3.02E-02 | -1.31361178 |
| TMEM8B | 1.24E-02 | -1.31425184 |
| FAM78B | 1.74E-03 | -1.31684455 |
| KAT2B | 7.37E-06 | -1.31738757 |
| SEMA6A | 6.60E-04 | -1.3180865 |
| P2RX2 | 5.55E-04 | -1.31860261 |
| RNVU1-19 | 1.64E-02 | -1.3186063 |
| UGP2 | 1.92E-05 | -1.3186107 |
| CACNA2D2 | 2.38E-03 | -1.31889113 |
| PPM1N | 1.76E-02 | -1.31920312 |
| PARD6A | 6.94E-03 | -1.32018733 |
| SERP2 | 2.66E-02 | -1.3210164 |
| ST6GALNAC3 | 4.51E-03 | -1.3213866 |
| RNF165 | 6.17E-03 | -1.32169186 |
| HADH | 4.06E-04 | -1.32180228 |
| SGCB | 4.67E-03 | -1.32183122 |
| GPR62 | 5.63E-04 | -1.32193347 |
| SCUBE2 | 1.58E-02 | -1.32196278 |
| OSBPL6 | 2.07E-03 | -1.32303207 |
| CYP1B1 | 1.59E-02 | -1.32319982 |
| MLYCD | 1.35E-04 | -1.32520136 |
| TDRD1 | 3.71E-02 | -1.32551449 |
| GPR143 | 6.13E-03 | -1.325522 |
| ANKRD34A | 3.00E-04 | -1.32609695 |
| SCN3B | 1.09E-02 | -1.32662577 |
| DYSF | 7.81E-03 | -1.32664394 |
| PARVB | 2.27E-03 | -1.32755309 |
| SUSD4 | 1.31E-02 | -1.33007068 |
| AGFG2 | 2.25E-02 | -1.33020549 |
| PPP1R36 | 4.97E-03 | -1.3311424 |
| PIP5K1B | 1.85E-02 | -1.33173203 |
| PPP2R3B | 7.25E-05 | -1.33279699 |
| PRKAG2-AS2 | 3.55E-02 | -1.33299454 |
| STUM | 3.66E-02 | -1.33332324 |
| C1orf115 | 2.07E-02 | -1.33356363 |
| SLC25A25 | 8.95E-04 | -1.33391197 |
| TRK-CTT2-2 | 2.81E-03 | -1.33546521 |
| ABHD11-AS1 | 6.85E-03 | -1.33567096 |
| BEX4 | 6.21E-03 | -1.33585904 |
| MIOS-DT | 7.73E-04 | -1.33726672 |
| RTL5 | 8.86E-03 | -1.33829489 |
| TSPAN7 | 2.49E-02 | -1.33834274 |
| SLITRK2 | 2.92E-03 | -1.34032771 |
| LINC01132 | 2.13E-06 | -1.34080924 |
| TRNL2 | 3.75E-04 | -1.34135459 |
| PDE2A-AS2 | 7.52E-03 | -1.34143395 |
| ASB8 | 1.59E-05 | -1.34316047 |
| ZNF844 | 2.73E-02 | -1.34404036 |
| NR4A2 | 2.75E-02 | -1.34440887 |
| KCNS3 | 3.38E-03 | -1.34651649 |
| ABO | 1.88E-02 | -1.3471548 |
| CORO2B | 1.53E-02 | -1.34778814 |
| PPM1L | 1.19E-04 | -1.34787768 |
| GATM | 3.04E-03 | -1.34811641 |
| ZEB1 | 6.89E-03 | -1.34849911 |
| SOD3 | 1.76E-02 | -1.35061767 |
| SOBP | 2.38E-02 | -1.3508082 |
| MYCN | 2.18E-02 | -1.35114638 |
| SYT17 | 4.50E-04 | -1.35198736 |
| ACO2 | 1.63E-05 | -1.35204991 |
| MAB21L2 | 1.22E-02 | -1.35294932 |
| SMIM10 | 2.28E-02 | -1.35336602 |
| ZFPM2 | 8.59E-03 | -1.35493713 |
| NUCB2 | 2.09E-04 | -1.35688239 |
| RILP | 2.77E-05 | -1.35783384 |
| FRY | 2.66E-02 | -1.3600157 |
| ROBO3 | 1.44E-02 | -1.36048273 |
| SERPINF1 | 1.92E-02 | -1.36070473 |
| P2RY14 | 9.14E-04 | -1.36217783 |
| HCG27 | 8.40E-03 | -1.3624893 |
| TECTA | 3.04E-03 | -1.36317512 |
| STXBP6 | 2.27E-03 | -1.36391903 |
| RGMA | 3.40E-04 | -1.36409871 |
| ANKFN1 | 2.84E-02 | -1.36549544 |
| ITGA9-AS1 | 2.98E-03 | -1.36749448 |
| EGLN1 | 3.99E-07 | -1.36911572 |
| SNAI3-AS1 | 1.50E-03 | -1.37060252 |
| CYP2U1-AS1 | 1.17E-02 | -1.37166137 |
| TAF12-DT | 1.23E-05 | -1.37177171 |
| CCDC28B | 6.35E-05 | -1.37264945 |
| PDZRN3 | 2.71E-03 | -1.37362891 |
| LHX6 | 4.44E-03 | -1.3741913 |
| ZNF676 | 3.19E-02 | -1.37574306 |
| OSER1-DT | 2.51E-04 | -1.37593181 |
| RNU12 | 3.10E-04 | -1.37675482 |
| TNS2 | 5.49E-03 | -1.37701543 |
| WDFY3-AS2 | 1.08E-04 | -1.3777726 |
| PPP1R3F | 7.69E-06 | -1.37778848 |
| RAB17 | 2.14E-02 | -1.3795867 |
| HS3ST1 | 2.20E-03 | -1.37961941 |
| DMTN | 8.87E-03 | -1.38032333 |
| SH2D1B | 4.85E-02 | -1.38206412 |
| SLC25A12 | 1.57E-05 | -1.38367674 |
| FGF14 | 2.91E-02 | -1.383775 |
| GAS1RR | 2.15E-02 | -1.38384159 |
| RPS6KL1 | 1.75E-05 | -1.38412146 |
| CATSPER3 | 1.66E-06 | -1.38664887 |
| SNED1 | 7.64E-03 | -1.38761527 |
| UQCRFS1 | 4.33E-06 | -1.3879936 |
| TNS2-AS1 | 7.62E-03 | -1.38807962 |
| SYNE1 | 5.77E-03 | -1.3901658 |
| ZNF667-AS1 | 1.76E-02 | -1.39120556 |
| TMEM266 | 5.29E-05 | -1.39150528 |
| MIR99AHG | 6.89E-03 | -1.3921605 |
| DGLUCY | 2.27E-05 | -1.393466 |
| PITX2 | 7.64E-03 | -1.39363448 |
| ALDOA | 6.77E-05 | -1.39482592 |
| PDE5A | 1.67E-05 | -1.39534513 |
| THSD4-AS2 | 3.16E-02 | -1.3953794 |
| POU2AF3 | 1.93E-03 | -1.39546611 |
| PBX1-AS1 | 8.15E-03 | -1.39625249 |
| CDC42EP5 | 3.59E-03 | -1.39758318 |
| YBX3P1 | 5.20E-04 | -1.3988228 |
| AVPR1A | 2.10E-03 | -1.39882665 |
| ARMCX3 | 5.01E-03 | -1.39920535 |
| LINC01587 | 1.42E-03 | -1.39993487 |
| BMPER | 4.56E-02 | -1.40002943 |
| ACYP2 | 2.87E-05 | -1.40011465 |
| TBX1 | 5.26E-04 | -1.40045882 |
| TNFRSF19 | 1.95E-02 | -1.40070678 |
| SYNGR1 | 2.03E-05 | -1.40121887 |
| RELN | 2.85E-02 | -1.40336121 |
| GIMAP1 | 3.48E-03 | -1.40347748 |
| ACADS | 6.26E-04 | -1.40532701 |
| RNU4-2 | 1.31E-04 | -1.40716215 |
| MACC1-DT | 3.06E-02 | -1.40844667 |
| MUC6 | 1.36E-02 | -1.41015799 |
| RAB44 | 2.48E-02 | -1.410815 |
| SLC26A2 | 6.88E-03 | -1.41293929 |
| FCER1A | 1.37E-02 | -1.41346031 |
| EHD3 | 3.39E-04 | -1.4152221 |
| CD99L2 | 5.64E-04 | -1.41545914 |
| RNU11 | 5.59E-05 | -1.41838185 |
| MAN1C1 | 3.69E-03 | -1.41950628 |
| PDE4D | 1.94E-03 | -1.41969772 |
| LINC01354 | 1.69E-02 | -1.42089465 |
| MTARC1 | 8.87E-03 | -1.42169545 |
| HHIP | 4.48E-02 | -1.42183801 |
| ZNF626 | 5.01E-03 | -1.42246446 |
| MTMR8 | 7.74E-03 | -1.42275749 |
| ATP6V0E2 | 1.76E-02 | -1.4235868 |
| EYA1 | 2.35E-02 | -1.42440722 |
| DIO2 | 7.65E-04 | -1.42441253 |
| TMEM74 | 6.17E-03 | -1.42460814 |
| USP44 | 5.61E-03 | -1.42509263 |
| SIRT4 | 3.61E-04 | -1.42526468 |
| SOX17 | 2.19E-02 | -1.42646382 |
| RNF112 | 5.65E-03 | -1.42789574 |
| ATF3 | 2.55E-02 | -1.42892758 |
| EMCN | 1.76E-02 | -1.42938666 |
| CLIC6 | 2.96E-02 | -1.43071924 |
| FAM47E | 1.82E-02 | -1.4309927 |
| DEPP1 | 9.00E-03 | -1.43099637 |
| ZNF677 | 1.17E-02 | -1.43343434 |
| LTBP4 | 6.83E-05 | -1.43437805 |
| FAT4 | 8.87E-03 | -1.43604102 |
| ATP6V0E2-AS1 | 6.28E-03 | -1.43730031 |
| STAG3 | 5.49E-04 | -1.43761908 |
| IL11RA | 7.20E-05 | -1.43803802 |
| SCRG1 | 6.04E-03 | -1.43828165 |
| ILDR2 | 1.44E-02 | -1.43996146 |
| ABCC9 | 4.30E-02 | -1.44063428 |
| CASTOR3P | 5.85E-04 | -1.4407664 |
| SUCLG2-DT | 1.81E-03 | -1.44283632 |
| LCN10 | 2.17E-02 | -1.4432141 |
| FOXO6 | 5.19E-03 | -1.44367837 |
| CPED1 | 2.01E-02 | -1.44373215 |
| SVIL-AS1 | 3.38E-05 | -1.44523512 |
| TRIM7-AS1 | 6.66E-04 | -1.44687846 |
| CLEC1A | 1.30E-04 | -1.4488475 |
| OXER1 | 1.19E-03 | -1.44905962 |
| ICA1L | 6.23E-04 | -1.4491602 |
| CCM2L | 1.02E-03 | -1.45054578 |
| CNNM3 | 3.76E-06 | -1.45061555 |
| NECAB1 | 3.74E-03 | -1.45451828 |
| TENT5C | 2.13E-02 | -1.45824365 |
| CLDN7 | 2.16E-02 | -1.45952699 |
| PGPEP1 | 3.52E-03 | -1.46269216 |
| SEMA3G | 1.32E-02 | -1.46298625 |
| PDE4B | 1.61E-02 | -1.46390746 |
| ANXA1 | 1.32E-02 | -1.4652512 |
| CRAT | 1.26E-02 | -1.46533751 |
| ITIH4 | 3.05E-02 | -1.46900449 |
| USH2A | 3.12E-03 | -1.46907008 |
| GULP1 | 7.36E-04 | -1.46940129 |
| CPT1B | 5.83E-04 | -1.46946768 |
| ZNF385B | 4.06E-03 | -1.46948813 |
| SBSPON | 1.74E-03 | -1.47007377 |
| STK33 | 3.81E-02 | -1.47147507 |
| LINC02975 | 6.64E-03 | -1.47182363 |
| TIGIT | 1.48E-03 | -1.47294767 |
| CCL2 | 5.80E-03 | -1.4731553 |
| TMEM220-AS1 | 2.92E-03 | -1.47387242 |
| FAM110B | 1.96E-02 | -1.47448944 |
| SHISA6 | 3.66E-02 | -1.47488349 |
| RNASE4 | 2.81E-02 | -1.47553886 |
| GUSBP5 | 1.07E-02 | -1.4827732 |
| NDUFS1 | 7.54E-08 | -1.48359707 |
| ROBO2 | 4.94E-02 | -1.484858 |
| THSD7A | 8.85E-05 | -1.48541755 |
| PACSIN3 | 2.70E-05 | -1.48625132 |
| LINC01238 | 5.28E-03 | -1.48795935 |
| TRPM5 | 3.13E-03 | -1.48921212 |
| NR2F1 | 2.88E-03 | -1.49072869 |
| CLDN5 | 3.16E-03 | -1.49121809 |
| TPPP2 | 9.95E-03 | -1.49316741 |
| KIAA1671-AS1 | 1.83E-05 | -1.49786774 |
| DAGLA | 1.34E-03 | -1.49882263 |
| NXPH3 | 8.59E-03 | -1.49944348 |
| LEAP2 | 1.22E-03 | -1.50051327 |
| FAM149A | 1.91E-02 | -1.50467197 |
| F8 | 7.66E-03 | -1.5052093 |
| HADHB | 5.25E-07 | -1.5060873 |
| PDGFRA | 6.71E-03 | -1.50629637 |
| ALDH5A1 | 4.06E-04 | -1.5063385 |
| LINC02981 | 3.65E-03 | -1.5064202 |
| SLC35F1 | 2.00E-02 | -1.50813059 |
| SNORA31B | 5.82E-08 | -1.51023557 |
| LCN6 | 2.93E-02 | -1.51070013 |
| PRKAG2-AS1 | 6.81E-03 | -1.5120144 |
| BMERB1 | 7.84E-03 | -1.51279022 |
| BTG2 | 2.07E-04 | -1.51477514 |
| RRAD | 9.38E-03 | -1.51622024 |
| STRADB | 2.38E-06 | -1.51943592 |
| INMT | 3.38E-02 | -1.52147165 |
| SIM2 | 4.73E-02 | -1.52185782 |
| ACSM5 | 3.89E-02 | -1.52213092 |
| ANXA6 | 4.67E-03 | -1.52366172 |
| IDH2 | 8.96E-05 | -1.52394331 |
| RAMP2 | 1.22E-03 | -1.52509811 |
| TRY-GTA5-5 | 2.54E-02 | -1.52517035 |
| RECK | 9.74E-04 | -1.52591709 |
| AIF1L | 5.52E-03 | -1.52684443 |
| CRTAC1 | 4.55E-03 | -1.52809809 |
| PDE1C | 7.80E-03 | -1.52886458 |
| RYR3 | 6.67E-03 | -1.52889947 |
| AMIGO1 | 3.31E-03 | -1.52959865 |
| ATP1A3 | 2.46E-02 | -1.52982988 |
| IDH2-DT | 4.36E-04 | -1.53016659 |
| FENDRR | 1.96E-03 | -1.53022526 |
| GPBAR1 | 3.34E-04 | -1.53099772 |
| TNS1 | 5.93E-03 | -1.53243028 |
| FAM78A | 4.42E-04 | -1.53542497 |
| FAM174B | 3.25E-03 | -1.53838723 |
| DAAM2-AS1 | 2.63E-03 | -1.53839133 |
| PLCB4 | 2.79E-02 | -1.53992312 |
| MAMSTR | 6.66E-04 | -1.54023442 |
| INS-IGF2 | 3.04E-03 | -1.5410307 |
| IL17D | 2.48E-03 | -1.54527896 |
| CYP27A1 | 3.64E-03 | -1.54593108 |
| IGF2 | 2.69E-03 | -1.54652745 |
| ZBTB18 | 3.17E-05 | -1.54748999 |
| LRRC4C | 6.69E-03 | -1.54782902 |
| PACRG | 3.99E-03 | -1.54853551 |
| YBX3 | 6.35E-06 | -1.55019482 |
| SH2B2 | 4.58E-04 | -1.55173441 |
| DIO3 | 2.13E-02 | -1.55205273 |
| PCBD2 | 2.50E-09 | -1.55333438 |
| RUNDC3B | 7.53E-08 | -1.5556465 |
| NEXN-AS1 | 4.21E-03 | -1.55635744 |
| PLPP3 | 1.15E-03 | -1.55669672 |
| IGFBP5 | 2.74E-02 | -1.55678924 |
| TPM1 | 1.52E-02 | -1.55694145 |
| UPB1 | 1.92E-03 | -1.55782398 |
| PCNT | 1.38E-07 | -1.55814056 |
| KCNK12 | 1.61E-03 | -1.55836984 |
| CYSTM1 | 3.91E-04 | -1.55966306 |
| PPL | 1.64E-02 | -1.56178855 |
| DKK2 | 3.82E-04 | -1.56206012 |
| C1orf162 | 8.52E-06 | -1.56437703 |
| CX3CR1 | 2.12E-03 | -1.56569336 |
| ACADM | 3.79E-08 | -1.56722167 |
| CHRNB1 | 4.59E-04 | -1.56933636 |
| CCDC141 | 2.80E-03 | -1.57234226 |
| KL | 1.71E-03 | -1.57336017 |
| CPAMD8 | 6.06E-03 | -1.57385068 |
| MAB21L1 | 2.38E-02 | -1.57515705 |
| ZBTB20 | 7.25E-04 | -1.57631774 |
| MUC1 | 1.60E-02 | -1.5783231 |
| LGI4 | 1.10E-03 | -1.5786685 |
| PPP1R12B | 6.48E-03 | -1.58015931 |
| FGF13 | 1.15E-02 | -1.58050964 |
| NPR1 | 5.84E-03 | -1.58057723 |
| GPR17 | 9.03E-04 | -1.5806853 |
| SYNE3 | 2.00E-03 | -1.58113892 |
| CACNA2D3-AS1 | 3.44E-03 | -1.58199481 |
| KLF9-DT | 1.46E-03 | -1.58326159 |
| IQCN | 6.21E-03 | -1.58492843 |
| TRIM7 | 6.02E-04 | -1.58507414 |
| DMGDH | 1.87E-02 | -1.58714177 |
| RASD1 | 1.42E-02 | -1.587372 |
| ALPK2 | 2.31E-02 | -1.58760287 |
| P2RX5 | 5.53E-03 | -1.58905304 |
| KCNJ2 | 3.20E-07 | -1.59025901 |
| SMIM10L2A | 1.26E-03 | -1.59520777 |
| ECHDC3 | 9.65E-03 | -1.59614848 |
| PCDH9 | 2.90E-02 | -1.59681552 |
| AMY2B | 1.22E-03 | -1.5969969 |
| ALKAL2 | 2.72E-03 | -1.59885883 |
| SNORD64 | 2.27E-03 | -1.6001145 |
| ZNF423 | 5.03E-03 | -1.6016423 |
| L3MBTL4 | 1.75E-03 | -1.60370477 |
| CCDC110 | 4.02E-02 | -1.60432459 |
| DAPK2 | 1.25E-04 | -1.61071905 |
| FOS | 1.37E-03 | -1.61133581 |
| CXCR2 | 2.01E-02 | -1.61261115 |
| IL32 | 4.57E-03 | -1.6133087 |
| AQP1 | 8.53E-04 | -1.61458141 |
| TRI-AAT2-1 | 2.03E-03 | -1.61512864 |
| PGR | 2.32E-02 | -1.61543381 |
| ZDHHC11 | 4.78E-05 | -1.6155611 |
| CPXM2 | 4.44E-03 | -1.61560431 |
| MIR3918 | 6.32E-06 | -1.61817971 |
| RERG | 1.63E-03 | -1.61844955 |
| ZNF385D | 2.55E-02 | -1.6186113 |
| ZDHHC15 | 3.54E-03 | -1.61980486 |
| FMN2 | 2.75E-02 | -1.62090236 |
| ABCB1 | 2.04E-05 | -1.62164583 |
| PDE2A | 6.97E-04 | -1.62195737 |
| SLC25A42 | 5.92E-06 | -1.62271439 |
| BMS1P14 | 4.62E-04 | -1.62318875 |
| TSPYL5 | 3.03E-03 | -1.62400582 |
| CCDC69 | 2.37E-04 | -1.6245443 |
| JDP2-AS1 | 1.79E-04 | -1.62834929 |
| HSPB8 | 2.55E-04 | -1.62836943 |
| PWAR5 | 1.11E-03 | -1.62852839 |
| MTLN | 3.84E-04 | -1.62891404 |
| CXCL12 | 1.04E-02 | -1.62934014 |
| CYP4X1 | 3.67E-03 | -1.63035471 |
| MYLK4 | 3.43E-05 | -1.63104115 |
| ROR1-AS1 | 8.70E-03 | -1.63263364 |
| RGS11 | 1.58E-03 | -1.63300211 |
| SYNPO | 1.25E-03 | -1.63908808 |
| TCEAL7 | 1.48E-02 | -1.64086313 |
| FAM221A | 2.25E-03 | -1.64200663 |
| C3orf18 | 2.10E-04 | -1.64213259 |
| BOC | 2.70E-05 | -1.64230435 |
| CYP2J2 | 9.85E-04 | -1.64340794 |
| OSR1 | 3.26E-02 | -1.64354038 |
| VSIG2 | 1.84E-02 | -1.64479868 |
| SEMA6A-AS2 | 7.59E-05 | -1.64502694 |
| TACR1 | 3.59E-04 | -1.64557502 |
| DENND5B | 1.34E-03 | -1.64613887 |
| RGS5 | 1.22E-05 | -1.64868289 |
| ITM2A | 9.61E-04 | -1.64965975 |
| TPRG1-AS2 | 3.22E-02 | -1.6512926 |
| TLE2 | 1.21E-02 | -1.65451251 |
| SVIP | 2.55E-03 | -1.65748638 |
| DCN | 3.36E-03 | -1.66202392 |
| COL4A4 | 6.39E-03 | -1.66290296 |
| PWARSN | 9.85E-04 | -1.66300823 |
| NRN1 | 1.70E-03 | -1.66350478 |
| EPB41L4A | 1.05E-04 | -1.6677916 |
| NKAIN1 | 5.36E-03 | -1.66791119 |
| ADAM33 | 3.99E-03 | -1.66866427 |
| ATOH8 | 1.58E-02 | -1.66894992 |
| LINC01088 | 2.82E-02 | -1.66947593 |
| ACAA2 | 3.62E-04 | -1.67099714 |
| CLU | 7.46E-03 | -1.67105912 |
| LONRF3 | 3.12E-03 | -1.67208165 |
| ROR1 | 3.30E-04 | -1.67223192 |
| GADD45B | 7.37E-04 | -1.6728704 |
| LRRC37A4P | 8.41E-04 | -1.67295237 |
| TUB | 2.86E-03 | -1.68114182 |
| REEP2 | 1.76E-04 | -1.68497996 |
| NAALADL2 | 3.48E-03 | -1.68599152 |
| ST6GALNAC1 | 9.74E-03 | -1.68684524 |
| ATRNL1 | 6.85E-04 | -1.68699735 |
| SLC10A1 | 1.00E-03 | -1.68877195 |
| RASA4 | 1.65E-04 | -1.68911034 |
| LINC02900 | 1.01E-03 | -1.68937468 |
| MAP3K20 | 1.01E-04 | -1.69081162 |
| NT5M | 1.45E-05 | -1.69151051 |
| ABCA3 | 1.31E-03 | -1.69207544 |
| BTC | 2.92E-03 | -1.69303099 |
| AS3MT | 1.64E-03 | -1.69331381 |
| GNG7 | 1.25E-02 | -1.69409958 |
| KLHDC9 | 1.21E-03 | -1.69463106 |
| GGTA1 | 4.12E-03 | -1.69480842 |
| ANKRD20A4-ANKRD20A20P | 1.71E-03 | -1.69489458 |
| FMO1 | 1.52E-03 | -1.69605766 |
| FGGY-DT | 9.05E-04 | -1.69764804 |
| APBB1 | 1.53E-03 | -1.69787183 |
| CAB39L | 2.65E-05 | -1.69833203 |
| ITGA9 | 1.31E-04 | -1.70074954 |
| RBPMS2 | 4.86E-04 | -1.70124231 |
| EIF4E3 | 9.70E-08 | -1.70143135 |
| MEOX2 | 1.49E-02 | -1.70281492 |
| PAX3 | 4.17E-02 | -1.70427281 |
| BARX2 | 1.74E-03 | -1.70547811 |
| SYP | 2.42E-05 | -1.71002359 |
| LSMEM1 | 9.47E-05 | -1.71399095 |
| MAMDC2-AS1 | 6.70E-05 | -1.71498959 |
| ATP1B2 | 1.27E-03 | -1.71786221 |
| C3 | 9.70E-03 | -1.71819472 |
| TRQ-CTG6-1 | 1.90E-03 | -1.71843296 |
| C2CD4B | 1.96E-02 | -1.71923332 |
| CBX7 | 1.02E-04 | -1.7207002 |
| CCDC85A | 1.83E-03 | -1.72106283 |
| CACNA1D | 2.92E-04 | -1.72376973 |
| PDZRN4 | 3.51E-02 | -1.72400861 |
| ADRA2B | 7.91E-03 | -1.72521293 |
| C16orf96 | 1.60E-08 | -1.72525244 |
| TRNE | 2.51E-05 | -1.73023735 |
| NNT | 9.96E-06 | -1.73284397 |
| LMO2 | 9.80E-08 | -1.73333895 |
| PHKA1 | 2.26E-05 | -1.73576043 |
| CLYBL | 2.38E-06 | -1.74040754 |
| KLF15 | 4.23E-02 | -1.7411371 |
| ZFP2 | 1.04E-03 | -1.74181387 |
| SFRP1 | 2.54E-02 | -1.74316637 |
| LRRN1 | 5.35E-03 | -1.74588002 |
| CD34 | 1.76E-04 | -1.7473508 |
| TRI-AAT5-1 | 1.04E-02 | -1.74771561 |
| MACROD1 | 1.28E-05 | -1.74840409 |
| AADAC | 3.90E-02 | -1.75016949 |
| TPM3 | 8.93E-07 | -1.75019526 |
| PAIP2B | 1.71E-08 | -1.75360454 |
| BEND5 | 1.73E-03 | -1.75737633 |
| LYZ | 1.30E-02 | -1.75817596 |
| NRK | 3.28E-02 | -1.75892804 |
| LRRC66 | 1.06E-03 | -1.76032125 |
| VIT | 2.53E-02 | -1.76043252 |
| KCNQ4 | 8.70E-04 | -1.7612515 |
| CACNA2D1-AS1 | 6.84E-05 | -1.76207596 |
| CASTOR2 | 1.88E-04 | -1.76367507 |
| CYTL1 | 1.56E-04 | -1.76403241 |
| MAMDC2 | 2.93E-06 | -1.76522694 |
| B4GALT6 | 2.72E-04 | -1.7673436 |
| RASA4B | 1.18E-04 | -1.76877411 |
| LRRN2 | 1.60E-03 | -1.76956025 |
| PAQR8 | 3.34E-05 | -1.7696052 |
| MYH7B | 2.20E-06 | -1.77062884 |
| N4BP3 | 3.62E-05 | -1.77411029 |
| SNORD108 | 1.54E-03 | -1.77448856 |
| EBF1 | 1.22E-05 | -1.77558672 |
| NR4A1 | 7.68E-03 | -1.77846908 |
| PLCL2 | 2.29E-04 | -1.77877811 |
| CES3 | 4.49E-03 | -1.78002609 |
| PLA2G4C | 6.95E-04 | -1.78029704 |
| CNNM1 | 2.99E-03 | -1.78128397 |
| FXYD6 | 1.51E-03 | -1.78204763 |
| TRIM7-AS2 | 2.75E-06 | -1.78613641 |
| SLC14A1 | 5.67E-04 | -1.78821812 |
| MEGF10 | 5.45E-03 | -1.79865932 |
| DOCK3 | 2.22E-06 | -1.8005785 |
| IGSF11 | 1.81E-03 | -1.80248811 |
| STAB2 | 2.02E-05 | -1.8025203 |
| NEXN | 2.06E-04 | -1.8030439 |
| DIRAS1 | 9.13E-03 | -1.80482565 |
| RCSD1 | 2.54E-05 | -1.8070222 |
| FAM13C | 1.91E-03 | -1.80751958 |
| ZIM2 | 5.27E-03 | -1.80913219 |
| SLAIN1 | 2.36E-03 | -1.80939889 |
| ACACB | 1.04E-02 | -1.81039276 |
| METTL7B | 3.11E-02 | -1.81135769 |
| PRKAR2B | 1.84E-04 | -1.81437936 |
| OTOGL | 1.91E-03 | -1.81549412 |
| RADIL | 3.99E-04 | -1.81591271 |
| ATP6V0A4 | 3.57E-02 | -1.81810075 |
| PRELP | 1.58E-02 | -1.81855102 |
| CAPN6 | 2.83E-02 | -1.81860161 |
| FMO2 | 1.32E-02 | -1.81892604 |
| LRRC7 | 5.06E-04 | -1.81945928 |
| CADM3-AS1 | 6.05E-03 | -1.81950952 |
| TOB1-AS1 | 5.05E-05 | -1.81992264 |
| PHYHIP | 1.04E-03 | -1.82060096 |
| MUSK | 2.18E-03 | -1.8207306 |
| ARHGAP20 | 1.69E-04 | -1.82211848 |
| NRADDP | 3.18E-04 | -1.82431832 |
| F13A1 | 2.20E-04 | -1.82439877 |
| HGF | 1.81E-05 | -1.82458179 |
| DCC | 2.69E-03 | -1.82560898 |
| NKAIN3-IT1 | 4.35E-02 | -1.82582575 |
| GCSHP3 | 8.59E-08 | -1.82641421 |
| ACADL | 4.46E-02 | -1.82955477 |
| ECM1 | 1.35E-02 | -1.8304139 |
| ATP8A1 | 6.62E-04 | -1.83183086 |
| LMO1 | 6.78E-03 | -1.83314538 |
| FRAS1 | 8.31E-04 | -1.83351069 |
| MYH11 | 9.68E-03 | -1.83528337 |
| PAX9 | 1.44E-04 | -1.83855768 |
| SMARCD3 | 4.74E-05 | -1.84023432 |
| PODN | 1.45E-02 | -1.841394 |
| ANGPT4 | 2.15E-03 | -1.84185547 |
| RPAP3-DT | 4.28E-04 | -1.84353894 |
| GOLGA8M | 2.00E-07 | -1.843734 |
| ADORA1 | 6.32E-04 | -1.84480402 |
| SORBS2 | 7.82E-03 | -1.84507491 |
| USP2 | 1.49E-04 | -1.84550613 |
| ANGPTL1 | 7.82E-03 | -1.84562123 |
| TXNIP | 8.85E-05 | -1.84635325 |
| CLGN | 8.98E-03 | -1.84635825 |
| ADGRD1-AS1 | 1.71E-02 | -1.84752229 |
| TRPV6 | 2.53E-03 | -1.84890917 |
| MAP2K6 | 8.20E-04 | -1.85023619 |
| SLC17A7 | 6.79E-04 | -1.85325007 |
| DUSP5 | 2.43E-03 | -1.85549228 |
| METTL24 | 3.29E-03 | -1.85908151 |
| CLDN11 | 1.06E-03 | -1.8603209 |
| JAM2 | 1.35E-04 | -1.86201258 |
| SVEP1 | 3.57E-03 | -1.86225891 |
| ACOX2 | 1.62E-03 | -1.86559956 |
| PLA2G4C-AS1 | 3.63E-04 | -1.8658758 |
| KCNK3 | 3.74E-03 | -1.8694464 |
| ERLNC1 | 1.39E-04 | -1.8717581 |
| FOSB | 1.10E-02 | -1.87230972 |
| SLC7A2 | 1.10E-02 | -1.87258108 |
| NELL1 | 1.64E-02 | -1.87258824 |
| LEXM | 1.10E-02 | -1.87318592 |
| CDH19 | 1.19E-02 | -1.87382293 |
| B3GAT1 | 4.40E-03 | -1.87401296 |
| SH3BGRL2 | 7.79E-04 | -1.87651391 |
| CPEB1 | 5.52E-06 | -1.87655167 |
| CCN5 | 4.24E-02 | -1.87824338 |
| DM1-AS | 1.88E-05 | -1.88181343 |
| FYCO1 | 6.62E-07 | -1.88348239 |
| CYP2E1 | 1.73E-03 | -1.88384214 |
| MPC1 | 1.46E-11 | -1.88485721 |
| TGFBR3 | 1.82E-03 | -1.88571066 |
| TRMT9B | 4.27E-04 | -1.88574389 |
| CYSLTR1 | 4.09E-05 | -1.88816092 |
| TRND | 1.45E-05 | -1.88848284 |
| GPX3 | 2.23E-03 | -1.88850693 |
| LYL1 | 3.06E-11 | -1.89175956 |
| CLTCL1 | 9.29E-06 | -1.8919308 |
| LINC01315 | 9.35E-04 | -1.8932336 |
| MIR6886 | 3.25E-03 | -1.89365969 |
| ARNT2 | 1.89E-05 | -1.89866154 |
| SLC25A23 | 2.18E-10 | -1.89887772 |
| RAI2 | 3.00E-04 | -1.90274625 |
| LIMCH1 | 2.79E-05 | -1.90592748 |
| TRNK | 4.33E-06 | -1.90898656 |
| ENPP4 | 3.40E-03 | -1.90912258 |
| PPFIA2 | 3.09E-04 | -1.91001266 |
| NES | 1.93E-04 | -1.9107168 |
| SNED1-AS1 | 5.57E-04 | -1.9111121 |
| PALM | 1.07E-04 | -1.91269702 |
| TMEM100 | 1.04E-02 | -1.91397512 |
| CYP4Z1 | 5.22E-04 | -1.91464978 |
| TMEM170B | 3.17E-04 | -1.91466811 |
| DDO | 5.17E-04 | -1.91467348 |
| PAPPA2 | 2.48E-05 | -1.9147092 |
| PGM5 | 1.02E-02 | -1.91650325 |
| ITGA7 | 1.10E-02 | -1.9286242 |
| SLC1A7 | 2.11E-02 | -1.92922222 |
| CACNB4 | 4.97E-03 | -1.93041049 |
| ACAT1 | 1.04E-08 | -1.93047244 |
| PEG3-AS1 | 3.75E-03 | -1.93086678 |
| MAP3K7CL | 4.85E-05 | -1.93094914 |
| SV2B | 3.64E-04 | -1.93109178 |
| ZBTB16 | 2.96E-02 | -1.93156715 |
| SFRP5 | 5.95E-03 | -1.93205913 |
| GLRB | 1.59E-02 | -1.93235693 |
| ACVR2B-AS1 | 7.64E-05 | -1.93280042 |
| CADM3 | 2.13E-03 | -1.93304693 |
| CUTC | 3.99E-07 | -1.93319195 |
| ACSL6 | 8.35E-05 | -1.93393198 |
| ADGRF1 | 1.74E-02 | -1.93518227 |
| SLC29A2 | 1.14E-06 | -1.93621405 |
| TJP3 | 1.01E-02 | -1.93731487 |
| IKZF2 | 5.16E-06 | -1.93766401 |
| FRMD3 | 7.89E-05 | -1.94063826 |
| SLC16A7 | 1.20E-04 | -1.94179532 |
| RAMP2-AS1 | 1.58E-02 | -1.94477748 |
| EGR1 | 9.47E-05 | -1.94674449 |
| MIR4697HG | 4.33E-03 | -1.94773134 |
| TRG-GCC1-5 | 1.10E-03 | -1.94783097 |
| ANKRD20A1 | 2.60E-04 | -1.94880493 |
| COL28A1 | 6.72E-05 | -1.95218019 |
| PWWP3B | 5.72E-03 | -1.95265009 |
| RNF150 | 1.95E-03 | -1.95376054 |
| ACSL3-AS1 | 1.31E-04 | -1.95644716 |
| TPTEP1 | 2.59E-03 | -1.9589277 |
| ZNF727 | 2.48E-03 | -1.96262587 |
| MFAP4 | 6.60E-03 | -1.96330178 |
| NPHP1 | 1.09E-04 | -1.96414191 |
| IL34 | 5.17E-06 | -1.96464013 |
| USP6 | 2.65E-06 | -1.96608142 |
| PTCH2 | 2.11E-05 | -1.96769027 |
| FGF18 | 4.43E-04 | -1.96919049 |
| LINC02456 | 1.78E-05 | -1.97524072 |
| GALNT16 | 6.18E-03 | -1.9758938 |
| KCNA6 | 3.47E-03 | -1.97747342 |
| GPRC5B | 1.24E-04 | -1.97858853 |
| ITGA8 | 7.80E-06 | -1.97901237 |
| CYP2C19 | 1.46E-02 | -1.9798127 |
| FAM107A | 1.91E-02 | -1.98017422 |
| PLXNA4 | 3.31E-06 | -1.98147157 |
| GPIHBP1 | 2.23E-03 | -1.98183766 |
| DIO2-AS1 | 1.12E-07 | -1.98320148 |
| PRKCQ-AS1 | 5.08E-05 | -1.98321178 |
| SLC25A21 | 1.02E-05 | -1.98392747 |
| PDGFD | 1.15E-02 | -1.98533304 |
| TRP-AGG2-6 | 4.47E-03 | -1.98536627 |
| STIMATE-MUSTN1 | 1.01E-04 | -1.98859284 |
| MT1G | 8.30E-04 | -1.98997897 |
| ENTREP1 | 4.72E-05 | -1.99540252 |
| GRIK3 | 3.14E-02 | -1.99623852 |
| CHRNA1 | 1.41E-02 | -1.99748613 |
| PDZRN3-AS1 | 5.24E-04 | -1.9980841 |
| PEG3 | 2.94E-03 | -2.00134316 |
| WNK2 | 1.23E-02 | -2.00395688 |
| PRG4 | 1.42E-04 | -2.0040077 |
| CECR2 | 7.05E-04 | -2.00416556 |
| ABCA10 | 1.31E-05 | -2.00465625 |
| ACADSB | 3.01E-08 | -2.00489119 |
| COL4A3 | 2.03E-03 | -2.00596151 |
| PPARGC1A | 5.51E-04 | -2.00630085 |
| ITIH3 | 4.15E-04 | -2.00847134 |
| PTGFR | 4.64E-03 | -2.01178857 |
| SLC34A3 | 1.51E-03 | -2.01190237 |
| SLC19A3 | 2.03E-03 | -2.01527757 |
| C2orf88 | 1.69E-06 | -2.02031184 |
| NFIX | 1.41E-09 | -2.02041113 |
| KCNN3 | 9.20E-06 | -2.02147572 |
| NAT8L | 1.28E-03 | -2.02166395 |
| ANKRD20A2P | 2.22E-04 | -2.02581168 |
| MYH15 | 3.98E-05 | -2.02963223 |
| PRR15L | 4.84E-03 | -2.02964373 |
| LDHD | 6.32E-05 | -2.03069871 |
| MIR5087 | 5.95E-05 | -2.03169616 |
| GPC3 | 3.67E-03 | -2.03216851 |
| ADAMTSL3 | 7.10E-03 | -2.03487884 |
| CRHR2 | 1.03E-07 | -2.03523966 |
| ETNK2 | 7.45E-05 | -2.03529459 |
| SIX2 | 3.81E-03 | -2.03641228 |
| FEM1A | 4.15E-07 | -2.03654162 |
| ZNF835 | 1.96E-03 | -2.0393641 |
| SNAI3 | 1.45E-06 | -2.04050872 |
| TMEM132B | 4.63E-07 | -2.0415791 |
| BMP5 | 4.63E-02 | -2.04384832 |
| ART4 | 2.14E-05 | -2.04485443 |
| CYP2C9 | 8.48E-03 | -2.04510335 |
| FAM110D | 5.02E-05 | -2.0459968 |
| DMPK | 6.84E-06 | -2.04805049 |
| GALNT17 | 7.88E-04 | -2.04813827 |
| TULP2 | 5.04E-03 | -2.04967534 |
| TRNW | 6.77E-05 | -2.05024669 |
| BRINP1 | 1.82E-02 | -2.05040136 |
| DKK4 | 5.84E-03 | -2.05069756 |
| CHCHD10 | 1.38E-07 | -2.05149374 |
| SGCD | 3.74E-03 | -2.05201236 |
| KCNQ5 | 3.76E-05 | -2.0544462 |
| SOGA3 | 2.23E-04 | -2.05696581 |
| RRAGD | 2.00E-05 | -2.06038436 |
| PLCL1 | 3.45E-05 | -2.06351005 |
| SIX1 | 2.94E-04 | -2.06483867 |
| MYL6B | 1.11E-07 | -2.06709747 |
| NCMAP | 1.62E-02 | -2.06781026 |
| MYRIP | 3.64E-04 | -2.06837416 |
| XPNPEP2 | 9.10E-03 | -2.07127565 |
| ACKR1 | 5.66E-03 | -2.07156866 |
| CR1 | 1.53E-03 | -2.0739278 |
| SNTB1 | 1.57E-06 | -2.07646791 |
| LINC01140 | 9.01E-07 | -2.07667579 |
| MPV17L | 3.36E-03 | -2.07887089 |
| BMX | 2.82E-06 | -2.07918224 |
| TLCD4 | 1.71E-03 | -2.0801298 |
| EPM2A | 5.74E-06 | -2.08085728 |
| LPIN1 | 3.08E-12 | -2.08127412 |
| PDE11A | 2.18E-02 | -2.08146679 |
| ENDOU | 1.27E-02 | -2.08488366 |
| ADAMTS9-AS2 | 5.56E-05 | -2.08492483 |
| HBB | 1.85E-04 | -2.08654449 |
| LINC00702 | 1.35E-03 | -2.08859284 |
| CTTNBP2 | 3.86E-04 | -2.09009951 |
| ALDH1A1 | 3.21E-03 | -2.09172177 |
| HOGA1 | 6.62E-08 | -2.09295717 |
| HP | 4.15E-03 | -2.09343517 |
| RBP7 | 1.04E-06 | -2.09480503 |
| ABI3BP | 2.41E-03 | -2.09722436 |
| CEACAM1 | 1.05E-03 | -2.09983013 |
| IRX6 | 4.81E-03 | -2.10134773 |
| SLC16A6 | 1.02E-05 | -2.10247517 |
| GPT | 2.14E-05 | -2.10297516 |
| CIDEA | 1.13E-03 | -2.10561081 |
| ADGRG2 | 1.95E-03 | -2.10600412 |
| CNR1 | 1.31E-04 | -2.10905001 |
| PTPN5 | 9.80E-04 | -2.11089298 |
| NPY1R | 3.00E-02 | -2.1111189 |
| AGL | 3.10E-07 | -2.11240639 |
| ICA1 | 2.93E-05 | -2.11414796 |
| ZBTB47 | 3.18E-05 | -2.11474014 |
| PRPH2 | 6.78E-05 | -2.11499412 |
| MYH13 | 1.40E-03 | -2.11856368 |
| OVCH1 | 2.52E-05 | -2.12052668 |
| LINC01395 | 5.81E-04 | -2.1218896 |
| MUC4 | 3.12E-02 | -2.12632909 |
| FREM1 | 5.47E-07 | -2.12679635 |
| COL19A1 | 7.45E-03 | -2.12848967 |
| WIPF3 | 3.98E-04 | -2.12870651 |
| ZDHHC11B | 3.05E-06 | -2.12969614 |
| EYA2 | 3.47E-05 | -2.13025992 |
| MIR9718 | 2.98E-04 | -2.13043086 |
| SLC9A4 | 2.16E-02 | -2.1328411 |
| GYPC | 1.27E-06 | -2.13653214 |
| EYA4 | 1.44E-03 | -2.13669645 |
| PLEKHA6 | 1.23E-05 | -2.14295356 |
| CGNL1 | 4.74E-04 | -2.14430894 |
| DOK7 | 1.71E-04 | -2.14447907 |
| PYGO1 | 6.47E-04 | -2.1451334 |
| MMRN1 | 2.78E-03 | -2.15239592 |
| NR3C2 | 6.60E-04 | -2.15312974 |
| CFL2 | 5.73E-05 | -2.15380835 |
| CACNA2D1 | 4.83E-04 | -2.15609592 |
| TRV-CAC1-5 | 2.38E-04 | -2.15639413 |
| SLC47A1 | 3.20E-04 | -2.16078366 |
| EBF2 | 2.82E-06 | -2.16148029 |
| SERPINB2 | 1.54E-02 | -2.16253287 |
| ABCA8 | 4.24E-03 | -2.16603598 |
| GYS2 | 1.23E-02 | -2.16666437 |
| ALDH1L1 | 1.31E-02 | -2.17092335 |
| DLG2 | 1.19E-07 | -2.17699868 |
| SNTA1 | 1.75E-05 | -2.17703449 |
| ACTG2 | 3.88E-03 | -2.18231106 |
| TAS1R1 | 2.07E-07 | -2.18343786 |
| GOT1 | 1.92E-10 | -2.18396075 |
| ITIH6 | 8.52E-04 | -2.18446071 |
| MTUS2 | 3.99E-07 | -2.18590463 |
| DCX | 2.56E-03 | -2.18924157 |
| RETREG1 | 1.49E-05 | -2.19607392 |
| NTRK3 | 3.59E-03 | -2.1961924 |
| COBL | 1.29E-03 | -2.19832685 |
| PMP2 | 3.77E-07 | -2.19863491 |
| CMBL | 8.49E-05 | -2.2021564 |
| CACNA2D3 | 1.39E-07 | -2.20371264 |
| ANKRD20A3P | 3.56E-05 | -2.20729548 |
| KCNT2 | 2.47E-06 | -2.20943657 |
| CYP4F12 | 5.11E-04 | -2.21152127 |
| SVIL | 1.79E-08 | -2.21226976 |
| SCARA5 | 4.09E-03 | -2.21264502 |
| ALDH1A2 | 1.65E-02 | -2.21424418 |
| CEL | 9.06E-05 | -2.21844038 |
| ABLIM2 | 2.84E-07 | -2.2191704 |
| FYB2 | 7.78E-04 | -2.2196548 |
| MPZ | 5.92E-04 | -2.22657357 |
| THBS4-AS1 | 2.28E-03 | -2.22717539 |
| FTCDNL1 | 1.40E-06 | -2.2323752 |
| HPGD | 2.64E-04 | -2.23617792 |
| EMX2OS | 6.30E-04 | -2.23668138 |
| SYT8 | 2.17E-03 | -2.23896332 |
| IGFBP-AS1 | 2.33E-05 | -2.24098169 |
| CYP4B1 | 1.58E-02 | -2.2468605 |
| MAB21L4 | 2.07E-02 | -2.24930764 |
| HMGCLL1 | 2.47E-02 | -2.25084169 |
| RORC | 3.95E-03 | -2.25457953 |
| PLAAT3 | 1.83E-04 | -2.25613495 |
| RIC3 | 1.38E-06 | -2.26003097 |
| APOD | 4.87E-03 | -2.26096963 |
| NEXMIF | 3.18E-03 | -2.26637661 |
| LIFR | 2.82E-04 | -2.2686277 |
| PTN | 1.66E-04 | -2.27146683 |
| MAPK12 | 1.45E-05 | -2.27179122 |
| THBS4 | 5.31E-03 | -2.27658414 |
| RGN | 4.63E-04 | -2.27713614 |
| PLAC9 | 4.37E-06 | -2.28046012 |
| SELENBP1 | 3.08E-04 | -2.28055677 |
| AK1 | 4.38E-07 | -2.28220194 |
| HLF | 3.14E-05 | -2.28921749 |
| KRT33A | 9.01E-03 | -2.29050062 |
| TF | 1.82E-02 | -2.29421191 |
| GKAP1 | 9.18E-05 | -2.29644035 |
| TRPM3 | 1.02E-05 | -2.29675487 |
| PLAC8 | 6.10E-04 | -2.302238 |
| CYP21A2 | 5.77E-04 | -2.30345263 |
| MIR6717 | 1.72E-06 | -2.31150775 |
| ASB16 | 4.07E-07 | -2.31476698 |
| MEF2C-AS1 | 1.74E-04 | -2.31534879 |
| SPARCL1 | 1.63E-07 | -2.31947418 |
| ASB14 | 7.56E-11 | -2.32330131 |
| AKAP6 | 4.43E-05 | -2.32421431 |
| LRP1B | 1.05E-02 | -2.32620242 |
| DGCR6 | 2.82E-06 | -2.33056103 |
| CRYAB | 1.28E-05 | -2.33208181 |
| GADD45G | 3.24E-06 | -2.33290827 |
| ASPA | 1.14E-03 | -2.33801116 |
| FAM3B | 1.46E-02 | -2.33801298 |
| BTNL9 | 6.16E-07 | -2.34226373 |
| SPINK5 | 1.45E-02 | -2.34800789 |
| ADSS1 | 5.48E-04 | -2.34903668 |
| DTNA | 5.20E-04 | -2.35044639 |
| PGM1 | 1.84E-08 | -2.35449046 |
| CORO6 | 5.70E-05 | -2.36489213 |
| CYP3A5 | 1.35E-05 | -2.37134738 |
| ACTC1 | 1.87E-02 | -2.37226789 |
| SCN2B | 1.11E-05 | -2.37232181 |
| MYOM3-AS1 | 2.20E-05 | -2.37359313 |
| LINC02767 | 8.51E-05 | -2.3739273 |
| ADGRB3 | 4.48E-06 | -2.37977983 |
| PFKFB1 | 1.59E-05 | -2.383284 |
| TRA-TGC4-1 | 3.94E-04 | -2.38848156 |
| RIMKLA | 6.58E-04 | -2.38928966 |
| CABP5 | 4.78E-05 | -2.39152703 |
| CYP21A1P | 1.35E-04 | -2.39435025 |
| OCA2 | 7.75E-04 | -2.39485117 |
| SLITRK4 | 4.51E-03 | -2.39517371 |
| NIPSNAP3B | 9.64E-06 | -2.40176661 |
| ANK1 | 1.07E-04 | -2.40347675 |
| ADH4 | 5.18E-06 | -2.40363477 |
| ZNF853 | 7.13E-05 | -2.40481523 |
| SYNPO2 | 3.49E-03 | -2.41048199 |
| MLXIPL | 8.30E-03 | -2.41146254 |
| ENTPD8 | 1.25E-04 | -2.41305457 |
| GAS2 | 4.58E-06 | -2.41585197 |
| GET1-SH3BGR | 2.11E-07 | -2.41860741 |
| PLAAT1 | 3.30E-04 | -2.41868185 |
| FGF7 | 8.40E-05 | -2.41902322 |
| ANK2 | 5.94E-04 | -2.41983068 |
| MYH3 | 2.56E-04 | -2.42344209 |
| PPP1R1B | 2.71E-03 | -2.42784137 |
| HPSE2 | 7.13E-04 | -2.42794294 |
| GFRA1 | 1.02E-03 | -2.42811145 |
| C1QTNF7 | 5.56E-05 | -2.42870385 |
| KCNJ2-AS1 | 1.61E-07 | -2.43104249 |
| EPDR1 | 3.69E-04 | -2.43365278 |
| NDRG2 | 1.73E-06 | -2.43385533 |
| ZNF536 | 2.49E-06 | -2.4340924 |
| RCAN2 | 9.01E-05 | -2.4348468 |
| DDN | 4.37E-03 | -2.43517032 |
| SLC2A5 | 3.20E-07 | -2.43522805 |
| CYP17A1 | 7.59E-06 | -2.43741071 |
| NEU4 | 2.00E-03 | -2.43894096 |
| FAM166B | 2.44E-05 | -2.43929094 |
| NOVA1 | 1.45E-03 | -2.43999523 |
| WSCD2 | 1.01E-03 | -2.4446602 |
| CES1 | 1.59E-03 | -2.44553187 |
| LRRN4CL | 4.17E-06 | -2.46115415 |
| EPHB1 | 2.39E-07 | -2.47128975 |
| GSTM5 | 3.79E-04 | -2.4715389 |
| TOGARAM2 | 2.19E-06 | -2.47406505 |
| CNTFR | 4.57E-03 | -2.47559686 |
| BMS1P23 | 2.59E-08 | -2.4771616 |
| TMEM238L | 2.09E-03 | -2.47766485 |
| INSYN1 | 1.44E-05 | -2.47928216 |
| COL14A1 | 1.59E-08 | -2.48722579 |
| MITF | 9.10E-07 | -2.48882019 |
| MAPK4 | 1.71E-03 | -2.48932022 |
| IGF1 | 1.07E-05 | -2.49056048 |
| TCEA3 | 8.12E-07 | -2.49156854 |
| NEK10 | 1.01E-08 | -2.49245632 |
| ERBB4 | 2.03E-02 | -2.49854413 |
| PMEL | 6.81E-05 | -2.50162201 |
| NDNF | 3.73E-05 | -2.50235745 |
| LRRC20 | 1.09E-06 | -2.51185386 |
| PLIN4 | 5.43E-03 | -2.51751865 |
| LRRTM3 | 6.57E-03 | -2.51872241 |
| UBE2QL1 | 3.31E-04 | -2.51872706 |
| CCL14 | 1.88E-03 | -2.52208724 |
| AMELX | 8.10E-08 | -2.52292897 |
| FRZB | 1.23E-04 | -2.54140193 |
| HBA2 | 3.43E-05 | -2.5479584 |
| ALOX12 | 4.92E-05 | -2.55093076 |
| NRXN1 | 2.45E-07 | -2.55237023 |
| ALOX12-AS1 | 1.71E-06 | -2.56404293 |
| CCL15-CCL14 | 1.29E-03 | -2.56496716 |
| ADGRD1 | 2.50E-04 | -2.56536311 |
| PLIN5 | 3.13E-04 | -2.56736406 |
| ZNF106 | 1.78E-09 | -2.5696432 |
| CAVIN2 | 4.12E-10 | -2.56972208 |
| CNKSR2 | 3.57E-04 | -2.57017095 |
| HBA1 | 2.96E-05 | -2.57051947 |
| KCNK2 | 2.86E-06 | -2.57278682 |
| RAMP1 | 3.82E-06 | -2.5736955 |
| F10 | 1.51E-04 | -2.57439692 |
| TMEM132C | 7.93E-03 | -2.58781164 |
| ABCA6 | 3.27E-07 | -2.58823405 |
| PCOLCE2 | 1.21E-03 | -2.59039276 |
| AR | 9.51E-05 | -2.59211259 |
| IGSF10 | 1.17E-06 | -2.5928187 |
| ESRRG | 8.00E-03 | -2.5940803 |
| IGDCC4 | 1.95E-05 | -2.60410425 |
| GPD1L | 1.05E-13 | -2.60857917 |
| HOTS | 2.48E-05 | -2.61037946 |
| SPEG | 5.36E-06 | -2.61844514 |
| TIMP4 | 2.78E-04 | -2.62223642 |
| TMEM143 | 5.83E-10 | -2.62436476 |
| PRKN | 1.41E-06 | -2.62485699 |
| MIR675 | 8.30E-05 | -2.62651452 |
| ITGB1BP2 | 3.86E-07 | -2.62746778 |
| CDO1 | 2.63E-04 | -2.63635961 |
| SPTBN4 | 4.87E-07 | -2.64301009 |
| VIPR2 | 6.21E-04 | -2.66441858 |
| DYRK1B | 3.31E-08 | -2.66486185 |
| PPFIA4 | 6.15E-07 | -2.66523953 |
| AGT | 1.44E-04 | -2.67298204 |
| WSCD1 | 1.29E-05 | -2.67371479 |
| FAIM2 | 1.44E-05 | -2.67505342 |
| GPR27 | 1.64E-08 | -2.67669987 |
| AQP7 | 1.66E-03 | -2.67816373 |
| TMEM233 | 2.13E-06 | -2.67853532 |
| KIAA0408 | 3.23E-05 | -2.67984416 |
| SBK1 | 5.20E-04 | -2.68186101 |
| GALNTL6 | 2.71E-04 | -2.68754904 |
| GRIN2A | 6.02E-07 | -2.69027348 |
| LONRF2 | 3.18E-03 | -2.69447222 |
| CLCN4 | 6.65E-06 | -2.70062999 |
| H19 | 1.71E-05 | -2.70936361 |
| ADCYAP1R1 | 2.48E-05 | -2.70983178 |
| AOC3 | 9.43E-08 | -2.71349626 |
| PHYH | 1.63E-06 | -2.72776139 |
| PRSS44P | 7.22E-04 | -2.7302203 |
| RTN2 | 3.07E-09 | -2.73025299 |
| FHL3 | 1.69E-08 | -2.73220928 |
| FAM180B | 2.45E-03 | -2.73254898 |
| PRRT4 | 1.75E-08 | -2.73380404 |
| CHDH | 8.09E-06 | -2.73650591 |
| HSPB3 | 2.61E-05 | -2.73862841 |
| PRUNE2 | 3.37E-04 | -2.73955099 |
| LINC02884 | 1.47E-04 | -2.74083699 |
| SORCS1 | 2.77E-05 | -2.74579137 |
| PKNOX2 | 6.86E-07 | -2.74714498 |
| STBD1 | 4.06E-07 | -2.75085244 |
| PLA2G2A | 4.09E-03 | -2.75279279 |
| HMCN2 | 2.89E-04 | -2.75362947 |
| PCDH20 | 2.47E-04 | -2.76182694 |
| SHISA4 | 1.17E-05 | -2.77492695 |
| GAMT | 7.72E-07 | -2.77535542 |
| FAM47E-STBD1 | 3.52E-07 | -2.77584098 |
| PTGDS | 2.46E-10 | -2.78272371 |
| ADHFE1 | 8.46E-07 | -2.7884479 |
| CDH20 | 4.08E-07 | -2.78857702 |
| ARHGAP6 | 9.88E-11 | -2.78923355 |
| CASQ2 | 5.11E-04 | -2.79318154 |
| SCIN | 1.53E-04 | -2.79417672 |
| DOK5 | 2.06E-04 | -2.79700207 |
| RBM20 | 3.85E-12 | -2.80064491 |
| DMD | 3.62E-05 | -2.80078198 |
| LINC01697 | 3.45E-07 | -2.80861934 |
| CFAP46 | 2.22E-07 | -2.82097336 |
| JPH2 | 2.31E-05 | -2.82167271 |
| KCNN2 | 1.25E-06 | -2.82789808 |
| P2RX6 | 2.66E-08 | -2.82883455 |
| CHRNA10 | 7.51E-10 | -2.83392326 |
| DEPTOR | 6.64E-07 | -2.83809618 |
| CDNF | 6.05E-09 | -2.83930917 |
| GLB1L2 | 1.38E-07 | -2.84002713 |
| PFKM | 2.17E-09 | -2.84026305 |
| ASTN1 | 3.52E-03 | -2.84465661 |
| FHL5 | 9.97E-08 | -2.84843736 |
| EMP1 | 1.35E-07 | -2.84906854 |
| KRT13 | 7.31E-03 | -2.85007602 |
| COL6A6 | 6.85E-07 | -2.85036823 |
| SOX10 | 2.55E-06 | -2.85109786 |
| PTP4A3 | 1.73E-07 | -2.86203945 |
| FILIP1 | 8.50E-06 | -2.86377548 |
| CAP2 | 3.89E-09 | -2.87258307 |
| RSPO1 | 9.53E-08 | -2.88276353 |
| PLEKHB1 | 7.20E-07 | -2.89010376 |
| NOG | 1.27E-04 | -2.89361847 |
| CH25H | 6.69E-07 | -2.8999193 |
| YBX2 | 1.31E-05 | -2.90025828 |
| ABCA9 | 1.11E-09 | -2.9066846 |
| CADM2 | 2.27E-03 | -2.91233999 |
| LRRC39 | 2.91E-09 | -2.91390659 |
| SLC38A3 | 6.13E-05 | -2.92477177 |
| MUSTN1 | 9.76E-05 | -2.92755492 |
| JPH1 | 3.79E-08 | -2.93000332 |
| AOX1 | 4.17E-06 | -2.93158687 |
| SPOCK3 | 1.35E-04 | -2.9316356 |
| PRKCQ | 5.79E-09 | -2.93412738 |
| PCARE | 8.96E-08 | -2.94436603 |
| MAOB | 1.76E-07 | -2.95513449 |
| DPF3 | 2.80E-09 | -2.95765529 |
| SEMA3E | 4.94E-03 | -2.96185636 |
| PDE4C | 7.13E-06 | -2.96710045 |
| MEF2C | 3.90E-07 | -2.9684665 |
| LANCL1-AS1 | 7.05E-08 | -2.98196063 |
| MMP27 | 1.36E-06 | -2.98260118 |
| TESC | 4.43E-06 | -2.98523 |
| BHMT2 | 3.14E-09 | -2.99037483 |
| SAMD5 | 2.00E-08 | -2.99164446 |
| RXRG | 1.39E-06 | -2.99279677 |
| CLCA4-AS1 | 7.36E-06 | -2.99482141 |
| SLC6A1 | 3.83E-09 | -3.00202542 |
| FMO9P | 1.03E-04 | -3.00398281 |
| PKHD1L1 | 4.39E-06 | -3.00517028 |
| CFAP61 | 4.58E-07 | -3.01072841 |
| KLHDC8A | 2.21E-04 | -3.01092949 |
| PTGES3L | 8.73E-09 | -3.02233088 |
| PDE4DIPP2 | 1.51E-09 | -3.03045635 |
| C7 | 1.35E-03 | -3.03807185 |
| XKR4 | 5.57E-08 | -3.04608859 |
| PDK4 | 3.15E-04 | -3.05917972 |
| TMEM182 | 6.26E-10 | -3.06069999 |
| RAPSN | 4.54E-08 | -3.06203597 |
| COQ10A | 7.65E-10 | -3.06754549 |
| CHRND | 3.29E-04 | -3.06931815 |
| CLEC3B | 1.19E-06 | -3.07101309 |
| AGTR1 | 4.62E-04 | -3.07324607 |
| PLP1 | 1.82E-08 | -3.08272769 |
| KCNJ12 | 4.84E-06 | -3.09190111 |
| TNXA | 2.57E-05 | -3.0958058 |
| PLCXD3 | 1.43E-03 | -3.09767855 |
| CFD | 1.41E-06 | -3.09840585 |
| COLCA1 | 6.85E-10 | -3.10480389 |
| SLC6A4 | 1.12E-06 | -3.11020571 |
| SYNM | 5.73E-06 | -3.11031939 |
| MEF2C-AS2 | 2.00E-07 | -3.11034046 |
| WDR49 | 5.77E-12 | -3.11484693 |
| HIF3A | 3.19E-05 | -3.11984924 |
| MYOCD | 1.11E-07 | -3.12010657 |
| CACNB1 | 1.45E-08 | -3.14232708 |
| MYH1 | 3.71E-03 | -3.15395082 |
| NRG2 | 9.53E-08 | -3.15651106 |
| DYNAP | 3.47E-02 | -3.17042595 |
| USP13 | 3.63E-10 | -3.17460587 |
| WNK4 | 2.87E-08 | -3.18531676 |
| PNMT | 4.59E-06 | -3.19650961 |
| MT1M | 1.15E-06 | -3.20478616 |
| C6 | 6.37E-04 | -3.21033677 |
| LINC01091 | 1.69E-10 | -3.21719048 |
| PRKAG3 | 5.30E-06 | -3.22214972 |
| KCNA7 | 2.84E-06 | -3.22607584 |
| LVRN | 8.06E-07 | -3.23128881 |
| SLCO5A1 | 3.89E-09 | -3.23694129 |
| MYOPARR | 5.02E-05 | -3.23881266 |
| TNXB | 3.04E-06 | -3.24462526 |
| TMEM178B | 7.17E-06 | -3.26232146 |
| FTLP10 | 2.27E-03 | -3.27097635 |
| KLHL30 | 2.10E-06 | -3.27832641 |
| CHRDL1 | 2.49E-04 | -3.27999696 |
| SERPINA5 | 3.65E-06 | -3.2828656 |
| LINC01372 | 3.95E-09 | -3.28591124 |
| PGAM2 | 2.29E-10 | -3.30223083 |
| NCAM1 | 1.53E-06 | -3.31750285 |
| CAPN3 | 1.05E-11 | -3.31880669 |
| MT1A | 4.04E-05 | -3.33033638 |
| MYOG | 2.69E-04 | -3.33089259 |
| GMPR | 3.86E-07 | -3.33682581 |
| KRT78 | 2.19E-03 | -3.3451665 |
| CYP11A1 | 4.65E-07 | -3.35597889 |
| ALPK3 | 1.32E-09 | -3.35693087 |
| HMGCS2 | 6.16E-03 | -3.36044668 |
| SMTNL1 | 3.18E-08 | -3.36166057 |
| SLC4A4 | 4.26E-06 | -3.373161 |
| CDH15 | 7.16E-07 | -3.37350861 |
| NOS1 | 3.52E-06 | -3.37385424 |
| ADCY2 | 1.75E-05 | -3.37739012 |
| BIN1 | 2.20E-10 | -3.38272403 |
| PDLIM3 | 4.62E-06 | -3.38967281 |
| CRNN | 4.99E-03 | -3.39246333 |
| SPTB | 1.95E-11 | -3.39593388 |
| RNF157-AS1 | 1.66E-10 | -3.39774466 |
| SRPK3 | 2.84E-08 | -3.40208221 |
| CAPN14 | 1.47E-04 | -3.40969159 |
| SLC7A14 | 5.87E-07 | -3.42459814 |
| ADAMTS19 | 6.73E-07 | -3.42901126 |
| PTPRQ | 9.71E-14 | -3.45132421 |
| TMPRSS11BNL | 3.66E-03 | -3.451741 |
| LYVE1 | 6.77E-10 | -3.45651765 |
| LRRC38 | 9.55E-05 | -3.45831425 |
| MYOM3 | 3.60E-10 | -3.4589201 |
| TBX15 | 4.89E-07 | -3.46843264 |
| SNX31 | 3.17E-07 | -3.47007463 |
| PI16 | 1.01E-04 | -3.48743671 |
| CARNS1 | 4.87E-11 | -3.49396003 |
| RNF157 | 3.37E-11 | -3.4991924 |
| PPP1R3C | 3.61E-10 | -3.50702642 |
| ANKRD1 | 1.73E-04 | -3.51693776 |
| GREM2 | 7.09E-07 | -3.52702653 |
| C9orf152 | 3.17E-05 | -3.52923742 |
| ESRRB | 3.41E-08 | -3.53403128 |
| CLCA4 | 1.17E-04 | -3.54250189 |
| GRB14 | 9.90E-10 | -3.54375614 |
| SEMA6C | 6.35E-11 | -3.54535539 |
| NEURL1 | 6.84E-13 | -3.55657879 |
| DDIT4L | 1.69E-04 | -3.56323769 |
| TMOD1 | 2.37E-11 | -3.56645509 |
| EPHA7 | 2.92E-04 | -3.59108405 |
| PCSK2 | 2.04E-08 | -3.5936603 |
| MLIP | 1.65E-10 | -3.59475039 |
| RGS9BP | 8.53E-08 | -3.59885031 |
| ADRA1A | 1.22E-06 | -3.5988785 |
| SLC25A34 | 1.04E-12 | -3.60144148 |
| KCNT1 | 1.48E-08 | -3.60565712 |
| GDF10 | 9.76E-07 | -3.62077723 |
| GPD1 | 4.23E-05 | -3.62234911 |
| FSD2 | 2.30E-11 | -3.63964707 |
| EGF | 2.80E-09 | -3.64400009 |
| EPHA6 | 1.84E-09 | -3.65639239 |
| CILP | 1.71E-06 | -3.6671009 |
| SIM1 | 6.17E-05 | -3.6687042 |
| TUBA8 | 2.27E-12 | -3.6785804 |
| LTF | 2.14E-04 | -3.68445269 |
| CRISP3 | 2.16E-03 | -3.6927054 |
| VEGFD | 5.06E-13 | -3.72622972 |
| HSPB2 | 2.80E-09 | -3.72692157 |
| PPP1R27 | 7.84E-06 | -3.73026105 |
| MAFA | 3.45E-08 | -3.73559731 |
| PCAT7 | 3.70E-06 | -3.76099875 |
| ASB2 | 1.36E-10 | -3.7795566 |
| LINC03007 | 1.17E-04 | -3.78384569 |
| PDE4DIP | 2.94E-12 | -3.78454941 |
| FABP3 | 4.82E-13 | -3.79602106 |
| AMOT | 1.33E-10 | -3.80170534 |
| SLC8A3 | 1.91E-09 | -3.80711038 |
| CAMK2A | 1.32E-10 | -3.80851455 |
| MAPT | 2.09E-11 | -3.81142561 |
| OBSCN-AS1 | 9.58E-13 | -3.81866792 |
| SCN1B | 1.11E-09 | -3.84340157 |
| POPDC2 | 2.18E-10 | -3.86739505 |
| SEC14L5 | 8.64E-12 | -3.87832378 |
| CAVIN4 | 8.22E-08 | -3.88662897 |
| MYOD1 | 5.13E-05 | -3.89403223 |
| ASB5 | 7.62E-06 | -3.8950171 |
| MYOZ2 | 1.36E-05 | -3.90445573 |
| IP6K3 | 4.23E-07 | -3.90770399 |
| SBK3 | 1.52E-09 | -3.911853 |
| PAX7 | 5.03E-05 | -3.91604746 |
| MIR133A1HG | 1.04E-12 | -3.91917442 |
| RBM24 | 8.43E-08 | -3.93822511 |
| ACHE | 6.04E-09 | -3.94468313 |
| ATP1B4 | 2.96E-04 | -3.94675142 |
| CAND2 | 2.39E-11 | -3.9530915 |
| KRT4 | 5.17E-04 | -3.95529636 |
| TPM2 | 1.25E-10 | -3.96043521 |
| JSRP1 | 1.32E-06 | -3.96445895 |
| B3GALT1 | 1.35E-07 | -3.96703953 |
| ATP2B2 | 2.62E-06 | -3.97976858 |
| PERM1 | 2.81E-14 | -3.98173358 |
| PTGIS | 9.41E-09 | -3.98746283 |
| DPT | 6.76E-10 | -3.98998511 |
| PDE6A | 4.01E-12 | -3.99545938 |
| PRH1 | 6.81E-07 | -4.01191832 |
| UCP3 | 7.76E-12 | -4.01538294 |
| PRKAA2 | 2.67E-08 | -4.01760648 |
| CACNG1 | 9.53E-08 | -4.0251194 |
| PLPP7 | 6.88E-09 | -4.0480542 |
| TMPRSS11B | 2.05E-03 | -4.0582227 |
| S100A1 | 7.90E-10 | -4.06144423 |
| LRRC14B | 1.03E-07 | -4.06149701 |
| BEST3 | 3.39E-06 | -4.10075706 |
| COQ8A | 7.59E-14 | -4.10208285 |
| PADI2 | 5.38E-10 | -4.11317057 |
| CTNNA3 | 9.48E-07 | -4.12702655 |
| CKMT2 | 1.94E-13 | -4.12746081 |
| TRDN-AS1 | 5.25E-07 | -4.14499422 |
| SLC27A6 | 2.44E-08 | -4.158103 |
| ADPRHL1 | 8.07E-12 | -4.16706532 |
| ADH1B | 1.29E-04 | -4.18524641 |
| FXYD1 | 1.66E-09 | -4.18842635 |
| CLCN1 | 1.72E-12 | -4.19731194 |
| TRIM55 | 3.95E-09 | -4.20464161 |
| COX7A1 | 1.24E-14 | -4.20566576 |
| PRH1-PRR4 | 6.78E-06 | -4.23812598 |
| HCG22 | 5.96E-07 | -4.26158269 |
| SLC25A4 | 9.71E-14 | -4.26431777 |
| CSRP3 | 1.79E-05 | -4.26658759 |
| OBSCN | 5.17E-14 | -4.27095392 |
| SGCA | 2.81E-08 | -4.27831283 |
| ADIPOQ-AS1 | 4.87E-03 | -4.27833976 |
| FLNC | 4.25E-09 | -4.28050717 |
| MAL | 6.21E-05 | -4.28419611 |
| DES | 3.88E-06 | -4.29055118 |
| HSPB7 | 6.29E-07 | -4.30073784 |
| SH3BGR | 6.13E-11 | -4.30572433 |
| HSPB6 | 5.37E-09 | -4.31900633 |
| KLHL31 | 1.74E-11 | -4.33001589 |
| TNNT3 | 6.85E-07 | -4.33567498 |
| DMBT1 | 3.13E-03 | -4.33888094 |
| MIR7855 | 4.82E-09 | -4.3417342 |
| TMEM38A | 9.63E-12 | -4.34536739 |
| SYNPO2L-AS1 | 4.11E-10 | -4.35210007 |
| FLNC-AS1 | 1.59E-09 | -4.3540542 |
| MSTN | 1.97E-07 | -4.35526715 |
| LINC02487 | 6.46E-05 | -4.36105465 |
| ADIPOQ | 2.99E-03 | -4.37449297 |
| MYH8 | 3.97E-07 | -4.39000663 |
| MYO18B | 4.18E-08 | -4.39222425 |
| RMST | 4.01E-06 | -4.41357933 |
| SYNPO2L | 1.46E-10 | -4.42337175 |
| YIPF7 | 1.73E-07 | -4.4378207 |
| FHL1 | 8.27E-11 | -4.44955741 |
| ARPP21 | 7.11E-07 | -4.46462081 |
| TRIM63 | 2.14E-08 | -4.46636152 |
| LINC01405 | 4.50E-07 | -4.467193 |
| PKIA | 2.46E-13 | -4.47891506 |
| STAC3 | 1.01E-10 | -4.48390745 |
| LAMB4 | 7.90E-10 | -4.50649611 |
| ANO5 | 2.87E-08 | -4.50828988 |
| MYOM2 | 1.72E-08 | -4.51874241 |
| PPP1R1A | 2.27E-06 | -4.52259306 |
| SMPX | 1.63E-06 | -4.5376263 |
| MYH4 | 1.31E-07 | -4.54099993 |
| TRIM72 | 2.16E-08 | -4.55336165 |
| CACNG6 | 5.26E-06 | -4.59562634 |
| CLIC5 | 3.25E-12 | -4.59691141 |
| ANKRD23 | 8.85E-11 | -4.59820124 |
| STYXL2 | 4.87E-07 | -4.60129585 |
| TXLNB | 2.64E-12 | -4.60415588 |
| MYHAS | 5.38E-12 | -4.60829847 |
| PAX1 | 1.49E-04 | -4.61541766 |
| ARHGAP36 | 1.50E-07 | -4.61681805 |
| MYF6 | 1.19E-05 | -4.61724787 |
| KCNJ11 | 1.29E-12 | -4.6509764 |
| CAMK2B | 5.38E-11 | -4.67367435 |
| SMTNL2 | 3.54E-09 | -4.67969868 |
| ATP1A2 | 5.82E-08 | -4.68941851 |
| ABRA | 2.53E-09 | -4.69954873 |
| MYLK2 | 7.79E-09 | -4.70226326 |
| ASB12 | 3.35E-10 | -4.71286757 |
| TRIM54 | 4.41E-08 | -4.71727134 |
| EXTL1 | 3.77E-09 | -4.7179006 |
| HRC | 2.75E-09 | -4.71910117 |
| TNNI1 | 2.81E-10 | -4.72267521 |
| LMOD3 | 1.43E-08 | -4.73192042 |
| RBFOX1 | 2.85E-08 | -4.75418754 |
| TNNT1 | 6.35E-11 | -4.75527339 |
| APOBEC2 | 4.04E-12 | -4.76807908 |
| SCN4A | 7.85E-07 | -4.77054346 |
| AGBL1 | 1.40E-09 | -4.77204464 |
| CAV3 | 2.67E-08 | -4.77728867 |
| NPY6R | 2.30E-11 | -4.80993856 |
| KY | 3.30E-13 | -4.81082053 |
| SGCG | 1.76E-08 | -4.83787143 |
| TMOD4 | 2.07E-11 | -4.84150228 |
| RYR1 | 2.25E-11 | -4.85447743 |
| NT5C1A | 2.77E-07 | -4.85538064 |
| ASB4 | 2.36E-10 | -4.85540657 |
| KLHL38 | 1.57E-10 | -4.86235676 |
| SLN | 9.10E-07 | -4.8694585 |
| MYOM1 | 1.36E-10 | -4.87947994 |
| MYPN | 2.64E-08 | -4.88435924 |
| MUC21 | 2.64E-05 | -4.89581456 |
| TMEM52 | 6.79E-12 | -4.89819791 |
| XIRP2 | 3.52E-06 | -4.90042778 |
| HHATL | 4.77E-06 | -4.9089193 |
| TNNI2 | 2.58E-09 | -4.91666652 |
| KLHL33 | 9.07E-10 | -4.93332843 |
| DUSP26 | 2.03E-07 | -4.94760784 |
| ACTN2 | 1.95E-07 | -4.94874295 |
| DCT | 1.76E-07 | -4.94972671 |
| CA3-AS1 | 1.25E-13 | -4.95123641 |
| LRRC2 | 3.35E-11 | -4.95348995 |
| MYBPC2 | 7.74E-08 | -4.95635265 |
| SLC2A4 | 5.10E-10 | -4.97803361 |
| TRDN | 4.08E-07 | -4.98255295 |
| NMRK2 | 1.21E-06 | -4.98609384 |
| ANKRD20A11P | 3.42E-08 | -4.99644658 |
| C4orf54 | 6.77E-10 | -5.00202905 |
| MYADML2 | 3.15E-09 | -5.02127875 |
| NEB | 3.20E-09 | -5.02135341 |
| UNC45B | 2.51E-08 | -5.04634564 |
| TTN-AS1 | 1.46E-10 | -5.05183938 |
| SYPL2 | 1.17E-09 | -5.05534527 |
| TNNC1 | 5.07E-08 | -5.06659015 |
| C10orf71 | 9.87E-07 | -5.06755001 |
| HABP2 | 9.58E-13 | -5.08230482 |
| SRL | 1.32E-10 | -5.08568262 |
| LMOD2 | 4.18E-08 | -5.09055432 |
| CACNA1S | 1.51E-07 | -5.10395113 |
| MYOT | 4.01E-12 | -5.10530371 |
| FITM1 | 9.71E-14 | -5.10711004 |
| TTN | 2.58E-09 | -5.10935872 |
| PADI1 | 3.30E-07 | -5.11292283 |
| CMYA5 | 2.09E-11 | -5.13116842 |
| FAM240C | 5.37E-13 | -5.13901008 |
| AMPD1 | 3.98E-09 | -5.14626715 |
| DUSP13 | 6.35E-11 | -5.15924102 |
| VGLL2 | 1.21E-07 | -5.17529828 |
| KBTBD12 | 2.28E-12 | -5.21163416 |
| TCAP | 2.67E-08 | -5.21462226 |
| TYRP1 | 1.16E-09 | -5.21703207 |
| CYP4F29P | 1.96E-08 | -5.22175488 |
| CYP4F35P | 7.97E-10 | -5.22567174 |
| EEF1A2 | 3.30E-10 | -5.23935566 |
| MYL1 | 6.22E-08 | -5.24080563 |
| FNDC5 | 6.92E-16 | -5.25508132 |
| KRT36 | 7.63E-09 | -5.27605863 |
| ART3 | 1.92E-12 | -5.29754363 |
| KLHL41 | 1.39E-09 | -5.37003611 |
| MIR1-1HG | 1.76E-08 | -5.37727536 |
| ART1 | 1.82E-15 | -5.39239779 |
| ANKRD2 | 1.95E-13 | -5.43021399 |
| LDB3 | 6.52E-12 | -5.45622037 |
| STRIT1 | 2.21E-08 | -5.47429104 |
| KLHL40 | 1.01E-06 | -5.48469784 |
| MYOZ3 | 6.32E-12 | -5.49315162 |
| ASB10 | 7.90E-10 | -5.57592154 |
| MYL2 | 1.73E-08 | -5.58408314 |
| MYH2 | 1.79E-08 | -5.58869612 |
| METTL21EP | 4.00E-13 | -5.59004733 |
| CA3 | 1.74E-11 | -5.59704365 |
| NRAP | 1.66E-08 | -5.6178862 |
| RPL3L | 6.78E-14 | -5.62303998 |
| MYLK3 | 5.37E-13 | -5.63224515 |
| MYL11 | 4.36E-09 | -5.63277379 |
| ACTA1 | 8.77E-09 | -5.66447841 |
| HJV | 6.23E-09 | -5.69104006 |
| ANKRD20A5P | 1.08E-13 | -5.71015144 |
| COX6A2 | 1.33E-08 | -5.71564396 |
| SMYD1 | 1.22E-09 | -5.73276196 |
| MYBPC1 | 1.40E-09 | -5.75298678 |
| DLK1 | 5.46E-10 | -5.79492654 |
| TNNC2 | 4.91E-11 | -5.79670109 |
| PEBP4 | 6.35E-11 | -5.80755212 |
| CASQ1 | 1.09E-10 | -5.80780596 |
| MYOZ1 | 1.27E-10 | -5.82253497 |
| FBP2 | 1.05E-13 | -5.84020669 |
| MB | 2.84E-11 | -5.84919437 |
| ASB15 | 1.27E-10 | -5.86401779 |
| SLC36A2 | 1.92E-12 | -5.87601913 |
| XIRP1 | 1.10E-11 | -5.89887016 |
| PPP1R3A | 6.32E-08 | -5.90459222 |
| PRH2 | 2.95E-08 | -5.93206695 |
| ZG16B | 2.56E-06 | -5.93837913 |
| MYH6 | 1.53E-09 | -5.97708046 |
| ASB11 | 2.07E-11 | -6.01494414 |
| CKM | 4.73E-11 | -6.12715001 |
| ATP2A1-AS1 | 7.59E-14 | -6.19435122 |
| MYL3 | 6.84E-13 | -6.24973672 |
| ATP2A1 | 1.80E-13 | -6.2773877 |
| ENO3 | 2.32E-15 | -6.42090229 |
| MHRT | 7.56E-11 | -6.47239339 |
| MYH7 | 9.29E-12 | -6.56613336 |
| MYOC | 2.03E-07 | -6.57746656 |
| PPDPFL | 2.66E-11 | -6.61212354 |
| FBXO40 | 7.59E-14 | -6.61444987 |
| PYGM | 2.30E-14 | -6.77585928 |
| DHRS7C | 1.20E-12 | -7.58813281 |

DEG, differentially expressed genes; FDR, false discovery rate; FC, fold change
