# Supplementary figures and images for: Malignant Transformation of Normal Oral Tissue to Dysplasia and Early Oral Squamous Cell Carcinoma: An In Silico Transcriptomics Approach
Source: Anal Cell Pathol (Amst). 2024 Sep 18;2024:6260651. doi: 10.1155/2024/6260651 (PMC11458300; doi:10.1155/2024/6260651)

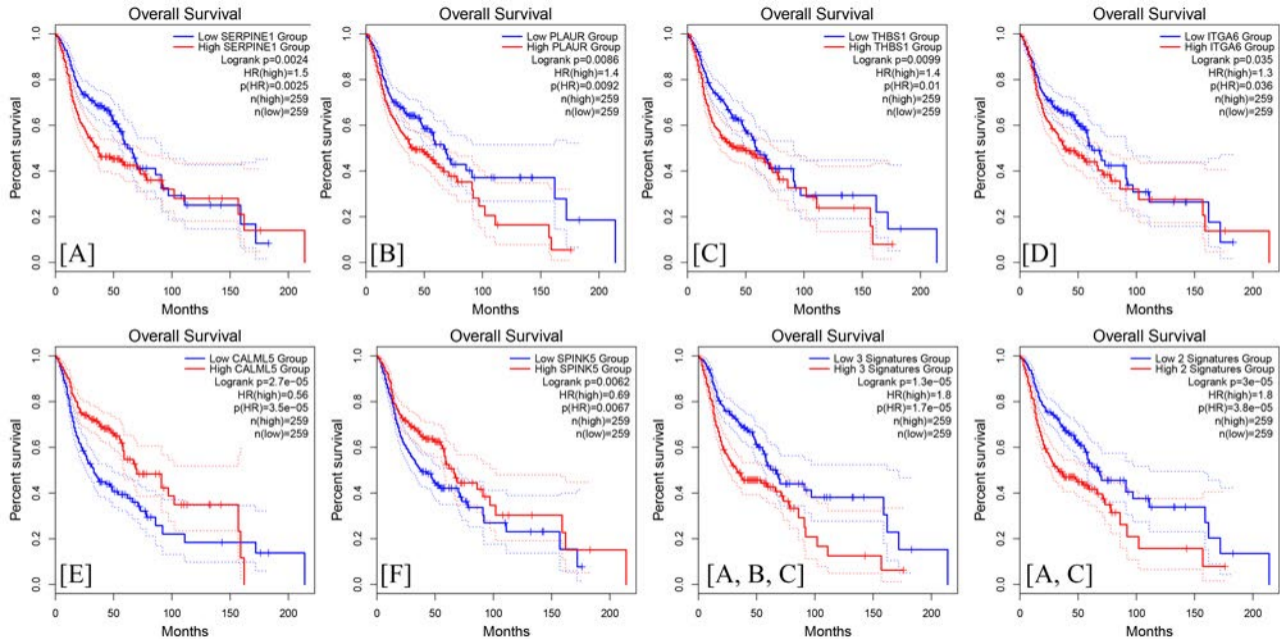

Supplement: Supporting Information S2: — Figure 1. Kaplan–Meier plots demonstrating the correlation between the expression of (A) SERPINE1, (B) PLAUR, (C) THBS1, (D) ITGA6, (E) CALML5, (F) SPINK5 and survival rate in HNSCC. Red and blue lines exhibit upregulated and downregulated genes, respectively. The x-axis and y-axis show the survival time of HNSCC patients and the percent of survival, respectively. The 95% confidence interval is shown with dotted lines. HNSCC, head and neck squamous cell carcinoma; HR, hazard ratio. [file 6260651.f2.pdf]
